# VisCap: inference and visualization of germ-line copy-number variants from targeted clinical sequencing data

**DOI:** 10.1038/gim.2015.156

**Published:** 2015-12-17

**Authors:** Trevor J. Pugh, Sami S. Amr, Mark J. Bowser, Sivakumar Gowrisankar, Elizabeth Hynes, Lisa M. Mahanta, Heidi L. Rehm, Birgit Funke, Matthew S. Lebo

**Affiliations:** 1 Princess Margaret Cancer Centre, University Health Network, Toronto, Ontario, Canada; 2 Department of Medical Biophysics, University of Toronto, Toronto, Ontario, Canada; 3 Laboratory for Molecular Medicine, Partners HealthCare Personalized Medicine, Boston, Massachusetts, USA; 4 Department of Pathology, Brigham and Women's Hospital and Massachusetts General Hospital, Boston, Massachusetts, USA; 5 Department of Pathology, Harvard Medical School, Boston, Massachusetts, USA

**Keywords:** copy-number variation, germ-line, molecular genetics, targeted clinical sequencing, visualization, VisCap

## Abstract

**Purpose:**

To develop and validate VisCap, a software program targeted to clinical laboratories for inference and visualization of germ-line copy-number variants (CNVs) from targeted next-generation sequencing data.

*Genet Med* **18** 7, 712–719.

**Methods:**

VisCap calculates the fraction of overall sequence coverage assigned to genomic intervals and computes log2 ratios of these values to the median of reference samples profiled using the same test configuration. Candidate CNVs are called when log2 ratios exceed user-defined thresholds.

*Genet Med* **18** 7, 712–719.

**Results:**

We optimized VisCap using 14 cases with known CNVs, followed by prospective analysis of 1,104 cases referred for diagnostic DNA sequencing. To verify calls in the prospective cohort, we used droplet digital polymerase chain reaction (PCR) to confirm 10/27 candidate CNVs and 72/72 copy-neutral genomic regions scored by VisCap. We also used a genome-wide bead array to confirm the absence of CNV calls across panels applied to 10 cases. To improve specificity, we instituted a visual scoring system that enabled experienced reviewers to differentiate true-positive from false-positive calls with minimal impact on laboratory workflow.

*Genet Med* **18** 7, 712–719.

**Conclusions:**

VisCap is a sensitive method for inferring CNVs from targeted sequence data from targeted gene panels. Visual scoring of data underlying CNV calls is a critical step to reduce false-positive calls for follow-up testing.

*Genet Med* **18** 7, 712–719.

## Introduction

Targeted DNA sequencing of disease-associated genes has long been a mainstay of clinical genetic testing to uncover causative sequence variants. Implementation of next-generation sequencing (NGS) platforms in clinical laboratories has expanded the genomic footprint of these tests,^1^ and panels testing tens to thousands of genes simultaneously are now offered by academic^2,3,4^ and commercial centers.^5,6,7^ Initially deployed for detection of single-nucleotide variants and small insertions or deletions, many of these panels target genes for which copy-number variation is also an important source of pathogenic genome variation.

Our laboratory initially deployed targeted NGS-based tests for causative sequence variants underlying multiple cardiomyopathies^2,3^ (Pan Cardiomyopathy v1, 1,016 exons in 46 genes; v2, 1,095 exons in 51 genes) and nonsyndromic hearing loss^4^ (OtoGenome, v1, 1,236 exons in 71 genes; v2, 1,231 exons in 70 genes). These panels were originally launched as pooled bait sets that included probes targeting genes associated with Marfan syndrome (90 exons in 4 genes). However, these are diseases to which germ-line copy-number variants (CNVs) are also important contributors, and additional gene-specific copy-number assessments have historically been performed in parallel, resulting in increased cost and overall test complexity.

Several software tools have been developed to infer copy-number alterations from exome- and genome-scale NGS data.^8,9,10,11,12,13^ These methods often use complex data-normalization methods that reduce sensitivity for exon-level copy-number alterations and provide highly segmented copy-number regions, resulting in high false-positive rates.^14^ Proof-of-principle studies describing panel-based inference of CNVs have also been reported,^15,16^ but these methods do not offer clear communication of exon-level data underlying these calls or native support to tune data visualization outputs to reflect data thresholds derived empirically by user-specific clinical validation experiments. Therefore, we set out to develop a method to infer constitutional (i.e., germ-line) copy-number alterations from targeted, clinical NGS data, with particular focus on data visualization and quality control suitable for deployment in clinical laboratories.

VisCap is a CNV-detection and -visualization tool that compares the relative depth of read coverage across arbitrary sets of genome coordinates (e.g., exons) targeted in a set of DNA samples using the same laboratory workflow. In addition to data normalization and identification of candidate CNVs, VisCap provides graphical outputs to enable quality control and manual review in the context of exon-level data supporting and surrounding each CNV call. Our tool was tailored for use in clinical molecular genetic laboratories with a focus on usability, clear communication of results in the context of clinically validated data thresholds, common annotation of CNVs between text and graphical outputs, and flexible, panel-specific annotation to enable downstream CNV interpretation.

## Materials and Methods

### Generation of clinical sequencing data

Targeted DNA sequencing data were generated and analyzed as previous described.^2^ Briefly, DNA fragments were sheared to ~150–200 bp and barcoded adapters were ligated to facilitate multiplexed capture and sequencing. Batches of 7–10 DNA samples were pooled, and regions of interest were isolated by in-solution capture using custom RNA baits (Agilent SureSelect). These baits correspond to previously described Pan Cardiomyopathy^2,3^ and OtoGenome^4^ panels as well as genes associated with Marfan and related syndromes. Captured fragments were purified and 50-bp paired-end sequencing reads were generated on a single lane of a HiSeq 2000 or 2500 instrument. Sequencing reads were aligned using bwa version 0.5.8c^17^ and realigned around insertions and deletions using the Genome Analysis Toolkit^18^ version 1.0.4705 (GATK) IndelRealigner. Quality scores were recalibrated using GATK version 1.0.4705 BaseRecalibrator and the total depth of coverage across each genomic interval was calculated using the GATK version 1.0.4705 DepthOfCoverage tool.

### VisCap software code availability, dependencies, and inputs

VisCap is a publicly available (**Supplementary File S1** online, https://github.com/pughlab/viscap) CNV-detection and -visualization tool written in R (http://www.r-project.org) for analysis of targeted NGS data derived from hybrid-capture experiments. VisCap version 0.8 and R version 2.15.1 were used for the analyses in this report, as were dependent R libraries “gplots” version 2.11.0, “zoo” version 1.7–11, and “cluster” version 1.15.2. This program can be executed using the Unix command line or through a Windows graphical interface dependent on the R “winDialog()” command. As input, VisCap reads a directory containing interval summary files generated by the GATK DepthOfCoverage tool. For each sample, these files contain a summary of the total coverage of each genome region interval listed in a reference interval list. For the panels described in this report, each genomic interval corresponded to a single exon, although in practice these regions may be of any length and genomic location acceptable by the GATK DepthOfCoverage tool. A description of each output file is provided in **Supplementary Table S1** online.

### Normalization and visualization of sequence coverage data

**Figure 1:**
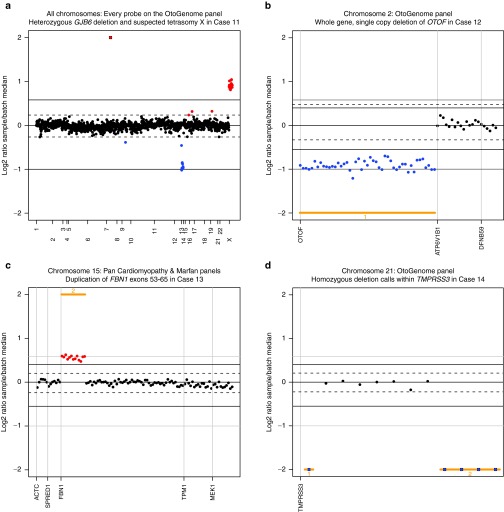
**Example of VisCap chromosome-level outputs**. Fractional depth of coverage values for each genomic interval (black dot) sequenced on a targeted panel plotted as log2 ratios against the median of a reference set of samples analyzed using the same panel and laboratory workflow. Copy-number variants supported by multiple consecutive exons with log2 ratios outside user-defined thresholds (solid black lines at −0.55 and 0.40, in this case) are color-coded: gains are red and losses are blue. An orange marker denotes the affected genomic segment with a number corresponding to the CNV identifier listed in the output text file. This plot is overlaid with guidelines depicting the two sets of user-defined thresholds used for copy-number variation (dashed = whiskers derived from the boxplot denoting the sample's overall data distribution; solid black = fixed, user-defined thresholds) and the theoretical log2 ratios (solid light gray) for single-copy gains and single-copy losses. Labels on the x-axis mark the first interval of each group of exons (commonly a gene name) as specified in the list of intervals provided to the program (e.g., TP53_Exon1 and TP53_Exon2 would be marked by a single TP53 label underneath exon 1). Panels **a–d** contain examples of VisCap output plots from four cases selected from Table 1. (**a**) Whole-genome view depicting log2 ratios from all intervals from a single patient run on a large gene panel. This individual had a *GJB6* deletion known from previous testing as well as an unexpected gain of chromosome X. The patient was phenotypically female and had median log2 ratio of X-chromosome probes twice that of other females, suggesting a potentially undiagnosed sex chromosome abnormality. (**b**) Single-chromosome view of data indicating a full gene, single-copy deletion of *OTOF* in a patient with a loss-of-function mutation on the remaining allele, illustrating compound heterozygosity resulting in nonsyndromic hearing loss. (**c**) Example of a multi-exon copy-number gain within *FBN1* leading to Marfan syndrome. (**d**) Example of two homozygous, noncontiguous exon-level deletion calls within *TMPRSS3* in a patient with nonsyndromic hearing loss. Family testing found that both of the patient's parents were heterozygous for both deletion calls, suggesting the deletions are on the same allele and may be indicative of a complex rearrangement.

The initial step in the VisCap program is to generate a matrix of all intervals captured and the fraction of total coverage assigned to these intervals. These are derived for each sample from DepthOfCoverage “sample_interval_summary” files using total coverage values in the column “{sample name}_total_cvg”. Next, the sample-specific fractional coverage of each region is divided by the median for that region across the entire batch, where the number of samples to be batched together can be any size that provides a representative median coverage across all target regions. In our clinical workflow, a batch refers to a set of 7–10 DNA samples captured in a single multiplexed pool and sequenced in a single flow-cell lane of an Illumina HiSeq 2000 or 2500 DNA sequencer. These batch median-normalized data are stored as a matrix of log2 ratios that is written to a text file. To facilitate visual review of the data for each sample, log2 ratios are plotted by relative genome order (i.e., rank order, not to scale on genome), and data points supporting CNVs are color-coded and linked to a unique identifier listed in the text output (**Figure 1**).

**Figure 2:**
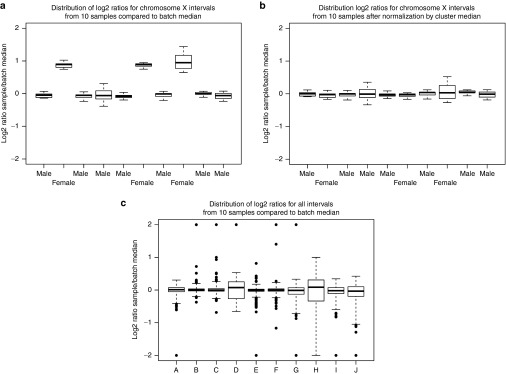
**Normalization of log2 ratios from probes on the X chromosome**. Distribution of log2 ratios from probes across all samples in a sequencing batch. Upper panels depict log2 ratios from probes on the X chromosome before (panel **a**) and after (panel **b**) inference and correction for sex composition within the sequencing batch used as a reference set. Lower panels depict log2 ratios from all probes from all samples run on a panel, including a sample that failed automated QC (panel **c**, case H) and was removed for an iterative run. Each boxplot depicts a 5-number summary dependent on the interquartile multiplier (x) set in the configuration file: 1) lower whisker is the lowest value to exceed the Q1 - x times the interquartile range; 2) lower hinge is the first quartile value (Q1); 3) middle line is the median; 4) upper hinge is the third quartile (Q3) value; 5) upper whisker is the lowest value to exceed Q3 + x times the interquartile range.

The X-chromosome requires further normalization because there are significant fractional coverage differences between males and females. Depending on the balance of males and females in the batch, males may display single-copy loss of chromosome X or females may display a single-copy gain. These patterns are evident from the presence of two clusters of boxplots depicting fractional coverage values across targets on the X-chromosome for each sample (**Figure 2**). These clusters are detected computationally by removing outlier probes and then partitioning all samples around two medoids, a more robust alternative to K-means clustering.^19^ To enable consistent visualization of CNVs from male and females in the same batch, the log2 ratios for each sample are normalized toward zero through subtraction or addition of the cluster median. To facilitate review of this procedure, boxplots of log2 ratios are generated before and after subtraction/addition of the cluster medians (**Figure 2a**,**b**, respectively). The program also outputs predicted sex for each case as an additional source of quality control (QC) data.

To derive thresholds for detection of copy-number gains and losses, boxplots are constructed using the R graphics “boxplot()” command. This provides a visual representation of the distribution of log2 ratios from each sample, as well as the five-number summary used for subsequent thresholding and quality control. The five-number summary includes upper whisker, Q3, median, Q1, and lower whisker. Upper (lower) whiskers are the greatest (lowest) values that fall within Q3 (Q1) plus a user-defined multiplier of the interquartile range (Q3–Q1). A sample fails quality control if either of the boxplot whiskers extends beyond the expected theoretical log2 ratio for a single-copy gain (0.58) or a single-copy loss (−1). To avoid skewing of the batch median used for normalization, failed samples are identified in the boxplot summary output and removed, triggering an iterative additional run with the remaining samples (**Figure 2c**). VisCap automatically repeats the analysis until all samples pass this automated QC. In our laboratory, an entire batch is failed if fewer than three samples pass QC. Files generated by each iteration are stored in separate output folders.

### Calling CNVs

The strategy to detect a gain or a loss is dependent on the distribution of log2 ratios for each sample as well as a set of user-defined thresholds. To be called, a copy-number gain must have log2 ratios that exceed (i) the user-defined gain threshold and (ii) the upper whisker of the boxplot representing the sample's data distribution. Copy-number losses must have consecutive probes below the loss threshold and the lower boxplot whisker. For our prospective study, these fixed thresholds were established based on the retrospective training set of 14 positive control samples (see Results, **Supplementary Figure S1** online), leading to a requirement for the minimum log2 ratio for gains of 0.40 and maximum log2 ratio for losses of −0.55. The boxplot IQR multiplier was set at 3.

### Confirmation of candidate CNVs by genome-wide bead array

To estimate our method's ability to identify both true regions of copy-number changes and copy-neutral regions, we analyzed a subset of 16 samples from the validation set using the 2.3 million–feature Illumina Human Omni2.5–8 BeadChip array at the Princess Margaret Genomics Centre, Toronto, Canada (http://www.pmgenomics.ca). Each sample was processed following the Illumina Infinium LCG assay protocol, hybridized to two BeadChips, stained as per Illumina protocol, and scanned on an Illumina iScan. The data files were quantified and normalized in the GenomeStudio version 2010.2 genotyping module using HumanOmni25-8v1-1_C.bpm manifest.

To call CNVs, data were exported from Genome Studio and uploaded into Biodiscovery Nexus v7.5 program. The significance threshold for segmentation was set at 1 × 10^−8^ and also required a minimum of three probes per segment and a maximum probe spacing of 1,000 between adjacent probes before breaking a segment. The log ratio thresholds for single-copy gain and single-copy loss were set at 0.13 and −0.23, respectively. Systematic GC wave correction was applied using Linear Correction with the HumanOmni2-5-8v1-1-C_hg19_illum_correction.txt file.

### Confirmation of candidate CNVs by droplet digital PCR

For more in-depth validation of VisCap, a minimum of one primer/TaqMan probe (Life Technologies, Carlsbad, CA) combination was designed to verify candidate CNVs called by VisCap. These oligonucleotides typically targeted an exon and were designed to avoid overlap with single-nucleotide polymorphisms and nonunique homologous regions when possible. Multiple probes were designed for larger or multigene CNVs. Droplet digital polymerase chain reaction (PCR) was performed using the Bio-Rad QuantaLife Droplet Digital PCR (ddPCR) device comparing the target of interest against a control probe targeting *RPP30*. To control for potential CNV of *RPP30*, we compared the copy number of *RPP30* against a second control, *AP3B1*. As a further control, all ddPCR assays were run simultaneously against DNA from the NA12878 cell line (Corriel). Results were analyzed using the QuantaSoft program and further normalized by dividing copy-number values by a sample-specific “Reference Correction Constant,” calculated as two-times the copy-number ratio of the *AP3B1* and *RPP30* reference genes. Samples with an *RPP30:AP3B1* ratio <0.9 or >1.2 were manually reviewed for quality or potential copy-number variation of reference genes. For each loci tested, a loss was called if the normalized value was <1.5, and a gain was called if the normalized value was >2.5.

## Results

Validation of this tool involved retrospective analysis of 14 cases with known CNVs detected by other clinical tests, as well as prospective analysis of 1,104 cases followed by confirmation of 27 candidate CNVs using ddPCR.^20^ The ddPCR-confirmed CNVs were used to estimate the positive predictive value of variant calls and to assess the added value of visual scoring of the graphical VisCap output.

### Retrospective analysis of 14 cases with known CNVs

To establish algorithmic thresholds to maximize the sensitivity and specificity of the VisCap program, we analyzed targeted DNA sequencing data from 10 hearing loss cases, 3 Marfan syndrome cases, and 1 hypertrophic cardiomyopathy case, with a total of 15 pathogenic CNVs known from previous testing (**Table 1**). This analysis uncovered 165 candidate CNVs at exon-level resolution, including all 15 pathogenic CNVs and 97 unique CNV calls. Of the unique CNVs, 95 were supported by data from only one or two exons and were often seen in multiple samples. We attributed many of these calls to specific baits with inconsistent capture performance. This inconsistency is probably due to extreme GC content because the small, recurrent CNV calls were enriched for exons with GC sequence <35 or >65% compared with the pathogenic and novel, larger CNVs (*P* = 0.002).

We reanalyzed all 14 cases after optimizing and implementing log2 ratio thresholds of 0.4 for gains and −0.55 for losses (see receiver-operating characteristic curve in **Supplementary Figure S1** online). These thresholds enabled detection of all pathogenic variants in our training set and reduced the number of CNV calls by more than 50%, from 165 to 77 (5.5 CNVs per sample on average; **Table 1**). Given the high sensitivity for pathogenic variants and relatively few additional CNVs for follow-up interpretation per sample, we next sought to test this configuration in a larger sample set.

### Prospective analysis of 1,104 cases and follow-up confirmation testing

To validate our VisCap configuration, we prospectively analyzed 1,104 cases analyzed in 113 batches using Pan Cardiomyopathy or OtoGenome panels (**Table 2**). Of these, 141 cases (12.8%) could not be scored; these consisted of 139 cases that were removed by the automated VisCap QC procedure (i.e., log2 ratio distributions were too broad to resolve single–copy number changes) and two cases that passed QC but were not analyzed due to failure of all other samples in the batch (minimum of three passing samples needed per batch). Across samples that passed QC, the median average target coverage was 724×, and 90% of cases had median target coverage between 329× and 1,684×.

From the 961 cases that passed QC, we inferred 3,005 candidate CNVs: 1,337 gains and 1,668 losses, with an average of 3.1 CNVs per sample, which is consistent with our training set. After removing single-exon CNV calls in exons frequently failing NGS analysis, 556 gains and 1,072 losses remained. Of these, 1,249 candidate CNVs corresponded to 105 unique calls seen in >1% of our cohort, likely copy-number polymorphisms or systematic artifacts; these calls were removed from further analysis. Consistent with the training cohort, the 105 recurrent calls were enriched for exons with GC content <35 or >65% compared with the remaining 378 candidate CNVs for follow-up (*P* = 10^−10^). The CNVs for follow-up were supported by 1–37 exons and represented 0.4 CNVs per case. Of note, panels with skewed GC content are more likely to benefit from removal of recurrent artifacts calls, as illustrated by the decrease in CNV calls on the Pan Cardiomyopathy panel (3.5 reduced to 0.4 CNVs/sample) that had a median GC content of 45% compared with 52% of the OtoGenome panel (0.3 reduced to 0.2 CNVs/sample).

To confirm these candidate CNVs, and to test whether bona fide CNVs were missed due to our VisCap thresholds, we reanalyzed six samples with CNVs called by VisCap and 10 samples with no candidate CNV calls using the 2.3 million–feature Illumina Human Omni2.5–8 BeadChip array. We confirmed five of six CNVs detected by VisCap and did not identify any CNVs within the targeted panel regions of the 10 negative samples (**Supplementary Table S2** online). The sixth CNV not detected by the microarray was confirmed by digital droplet PCR, representing a likely false negative from the array platform. For further confirmation, we selected 27 of 379 candidate CNVs plus 72 negative control regions with log2 ratios between −0.55 and 0.4 (our thresholds to issue a CNV call). To assess the ability of VisCap to discriminate potentially ambiguous CNV calls, we biased our selection of copy number–neutral regions toward those with values away from log2 ratio = 0 (i.e., copy number = 2) but still below our cutoffs for calling a CNV. No CNVs were detected in the 72 negative control regions by ddPCR. Of the 27 candidate CNVs, 10 were confirmed by ddPCR (**Table 3**), with high concordance between copy number inferred by VisCap and absolute copy number detected by ddPCR (*P* = 0.97).

The 17 calls that were not confirmed by ddPCR were enriched for smaller CNVs supported by three exons on average (range, 1–8 exons). However, we were not able to differentiate false-positive and true-positive calls based on size alone, because true positive CNVs were also found in this range. Therefore, we set out to assess whether visual scoring of data surrounding candidate CNV regions could be used as an effective method to identify false-positive calls and avoid unnecessary follow-up testing.

### Visual scoring of CNV calls

To determine whether visual scorers could differentiate the 10 true-positive from 17 false-positive calls, we trained four laboratory technicians to read and interpret VisCap plots (training material supplied as **Supplementary File S2** online), followed by testing using a set of practice variants (**Supplementary File S3** online). The technicians next scored the VisCap plots for the 27 candidate CNVs as either true-positive or false-positive calls without knowing the outcome of the ddPCR analysis (**Table 3**). All four technicians correctly identified all 10 verified CNVs. Although two of four technicians correctly categorized all false-positive calls, the other two technicians flagged 2/17 and 12/17 false positives for follow-up, highlighting the need for training and experienced review of these data. This illustrates the added value of human review of these data to reduce the overall false-positive rate while retaining high sensitivity.

## Discussion

We report here an open, flexible software program (and accompanying training documents) to detect and visualize germ-line CNVs from targeted DNA sequencing data. The software was specifically designed for implementation within a clinical laboratory, including laboratory-defined thresholds, static plots amenable for routine review, and standardized procedures and training documentation. This program has been validated in a clinical diagnostic laboratory and has been used for CNV analysis of >4,000 patients in our laboratory to date (1,118 described in this report), using multiple gene panel configurations, including Pan Cardiomyopathy and OtoGenome panels. Consistent with our prospective cohort, we anticipate that this method will scale well across a wide range of coverage levels (including low-pass sequencing for CNV detection only), as long as reference samples display limited batch-to-batch technical variability across target regions (hence our focus on enabling routine manual review of VisCap data). Although NGS data used for our study were generated from genomic fragments isolated by hybrid capture followed by sequencing on the Illumina HiSeq platforms, this method is amenable to alternative target isolation and sequencing platforms that generate depth-of-coverage data. As a proof of concept, we have previously applied this method to PCR amplicon sequencing data generated on the Ion Torrent platform (Life Technologies, Carlsbad, CA).^21^

In its current implementation, VisCap is highly sensitive (no known false negatives in our training set, 10 copy-neutral samples tested by genome-wide bead array, or 72 copy-neutral regions selected from our validation cohort) but has a relatively high false-positive rate (only 10/27 candidate CNVs targeted for verification were confirmed), a known issue in detecting small CNVs from targeted data.^14,16^ Additional intronic probes may aid in identifying small CNVs, albeit potentially at an increased cost. We have addressed the issue of a high false-positive rate through manual review of all CNV calls with relatively minor impact on our overall workflow and test turnaround time (4 CNVs/case run on a 46-gene panel, <1-min review per CNV). This approach will not scale well as gene panels continue to grow, and future improvements to the software will attempt to capture features that are important for manual review but not yet part of the CNV calling algorithm. Beyond confirmation of simple gains and losses, the value of manual data review is particularly valuable to uncover potential complex structural alterations such as *STRC* deletions in hearing loss.^4^ Therefore, visual interpretation of these data is currently a critical component of our clinical testing workflow. Although much of the informatics analysis is fully automated, we have demonstrated value in continued manual intervention and effective visualization for quality control and scoring of copy-number data generated by VisCap.

## Disclosure

This work was funded by internal operating funds of the Partners HealthCare Personalized Medicine. The Laboratory for Molecular Medicine is a nonprofit, fee-for-service laboratory that is a current or former employer of all authors and offers testing for cardiomyopathy, hearing loss, Marfan syndrome, and several other genetic disorders. The authors declare no other conflicts of interest.

## Figures and Tables

**Table 1:**
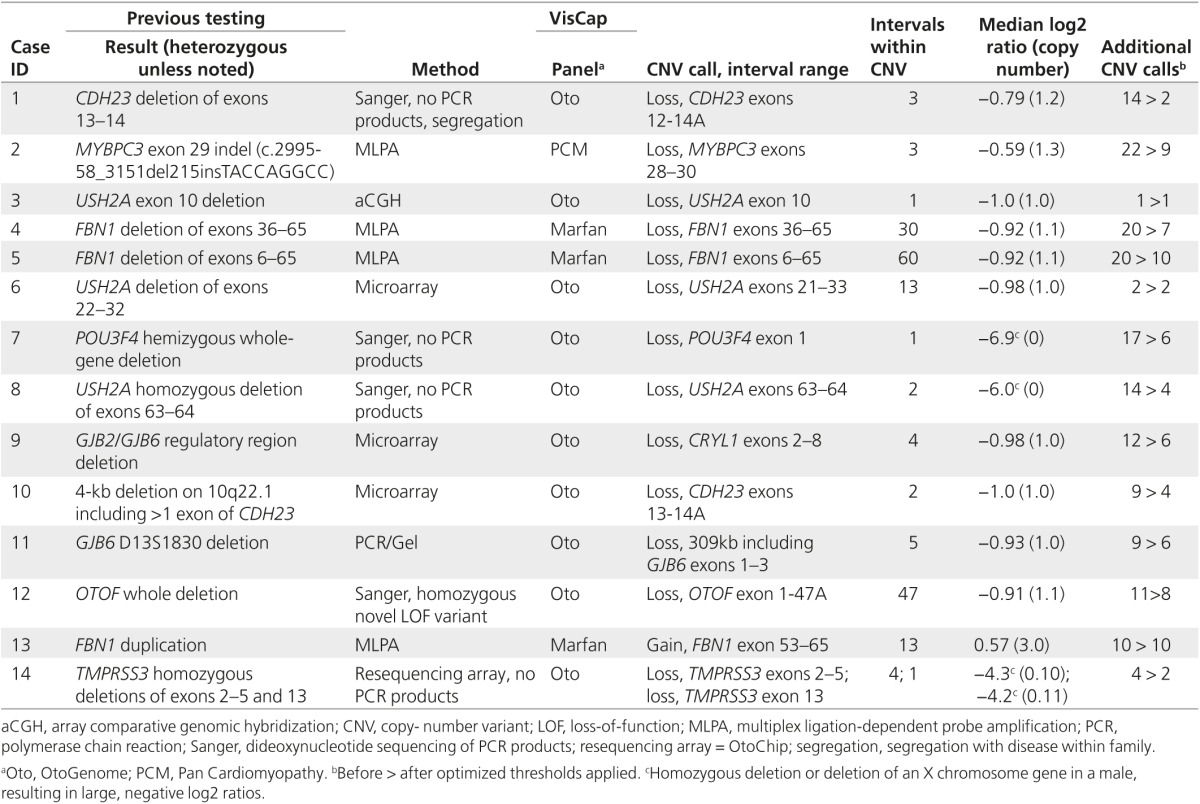
CNVs from 14 samples used for retrospective training of thresholds for VisCap algorithm

**Table 2:**
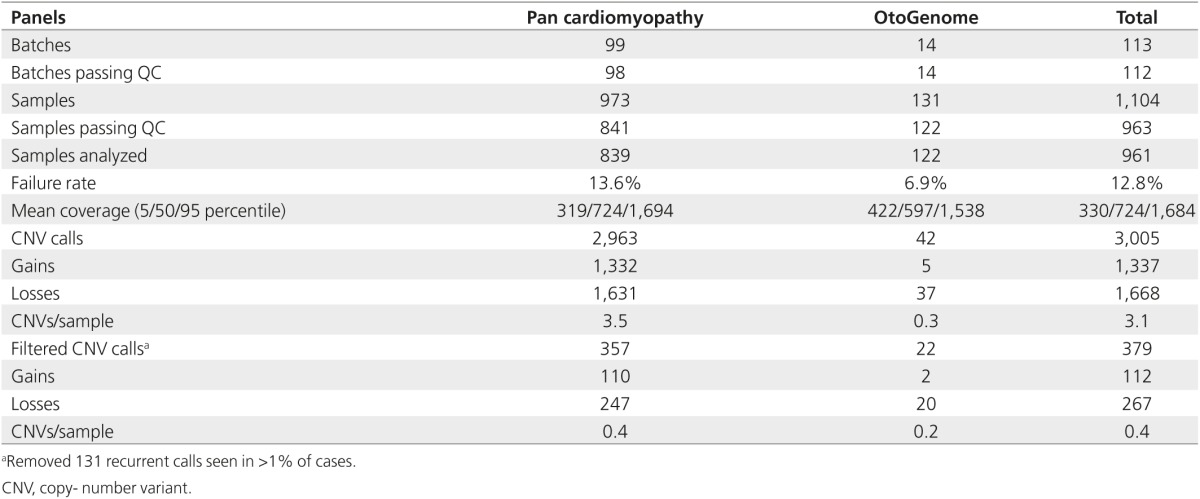
Summary of prospective validation cohort

**Table 3:**
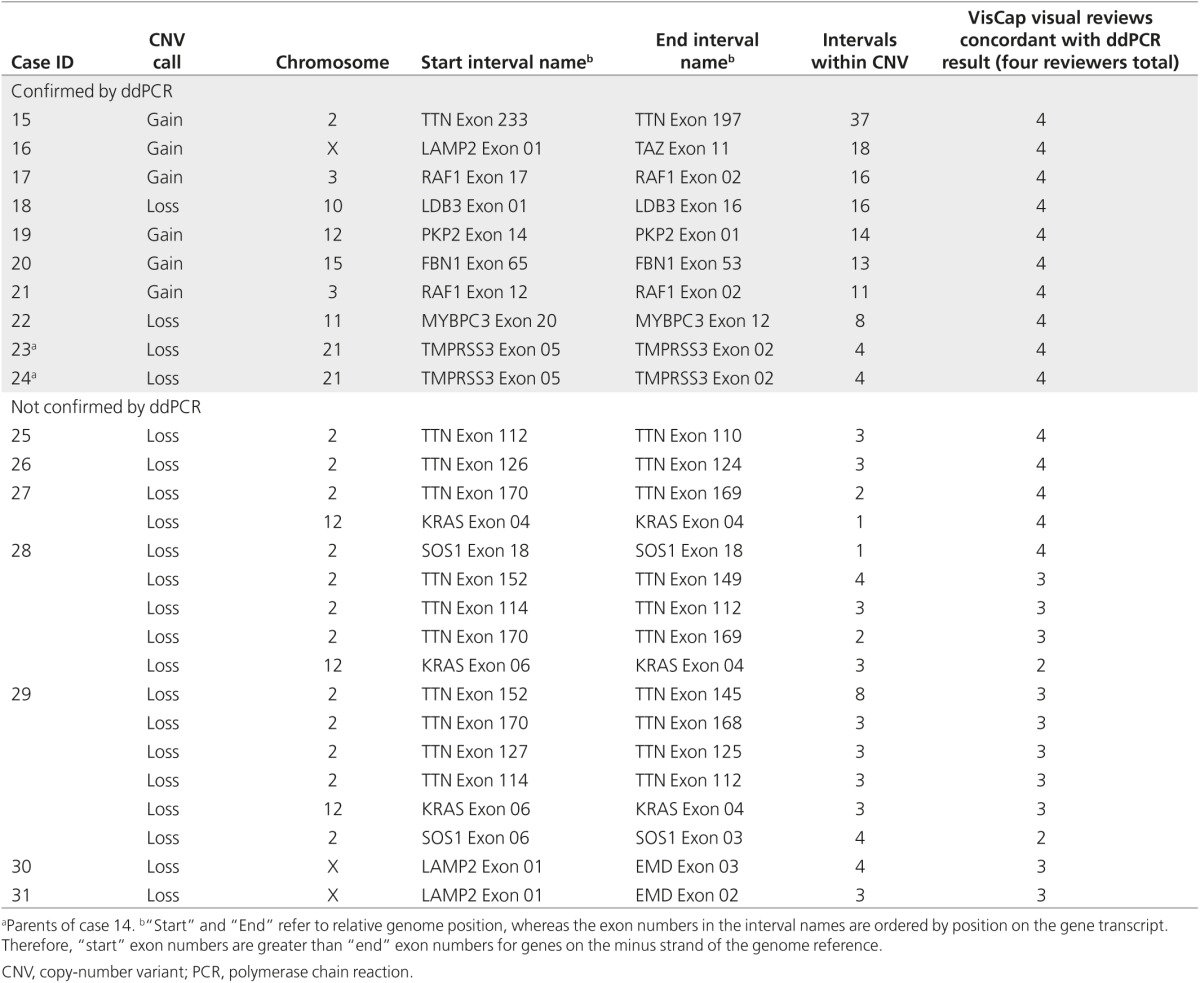
Candidate CNVs selected for confirmation by ddPCR and used for visual assessment training
