# Supplementary material for: VisCap: inference and visualization of germ-line copy-number variants from targeted clinical sequencing data
Source: Genet Med. 2015 Dec 17;18(7):712–9. doi: 10.1038/gim.2015.156 (PMC4940431; doi:10.1038/gim.2015.156)

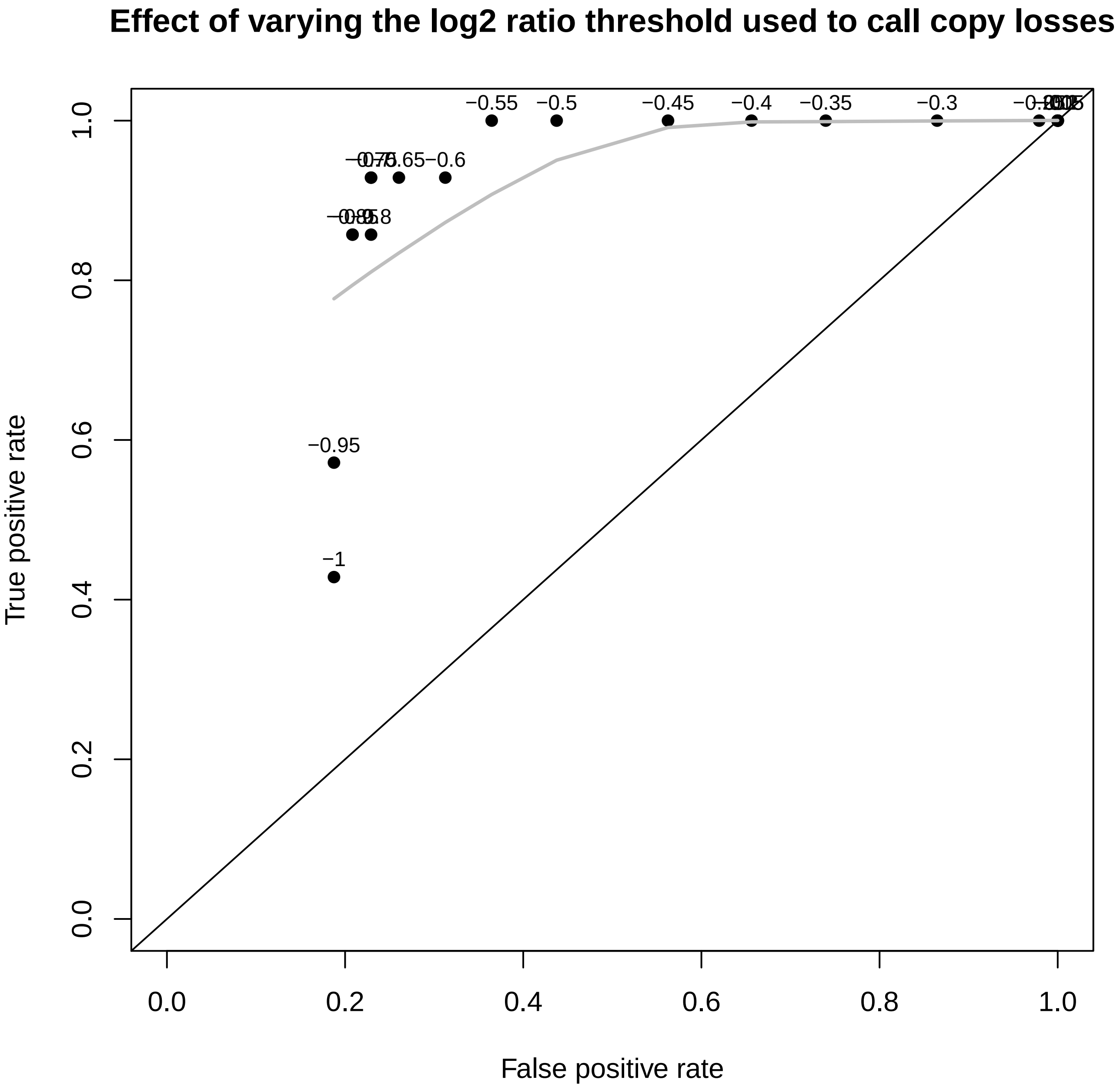

Effect of varying the log2 ratio threshold used to call copy losses
True positive rate
False positive rate
0.0
0.2
0.4
0.6
0.8
1.0
−0.55
−0.5
−0.45
−0.4
−0.35
−0.3
−0.6
−0.65
−0.95
−1

Effect of varying the log2 ratio threshold used to call copy gains

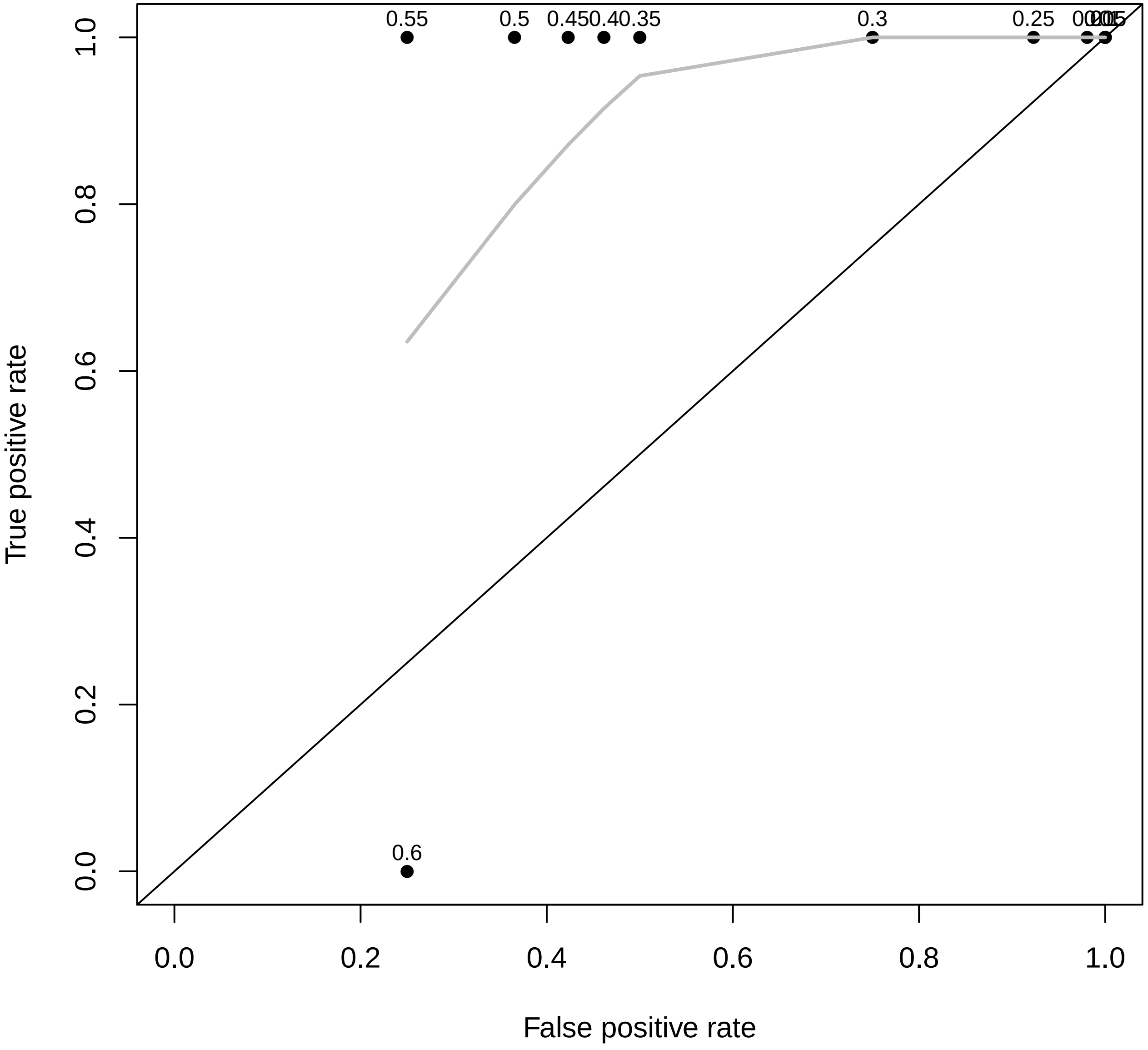

Supplement: Supplementary Information [file gim2015156x1.zip › Supplementary_Figure_1.pdf]
