# Supplementary material for: VisCap: inference and visualization of germ-line copy-number variants from targeted clinical sequencing data
Source: Genet Med. 2015 Dec 17;18(7):712–9. doi: 10.1038/gim.2015.156 (PMC4940431; doi:10.1038/gim.2015.156)
Supplement: Supplementary Information [file gim2015156x1.zip › Supplementary_File_2.pdf]

VisCap Visual Assessment Training

Version 02

Background

What is VisCap?

- Copy number detection and visualization tool
- Uses next-generation sequencing data (coverage)
- Compares sample coverage against median coverage of all other samples in same batch
  ($coverage_{sample}$ / $median\_coverage_{batch}$)
- Output displays the log2 ratio for each exon (y-axis) and the genomic position (x-axis)
- CNV is called when:
  1) Exon log2 ratio deviates significantly from other exons within the sample
  2) The exon log2 ratio passes fixed thresholds

Why do we need visual assessment of the data?

- VisCap is a sensitive caller giving numerous false positives
- Computational filters help to lower the number, but many false positives remain
- Most remaining (post-filter) false positive calls are easily discernible in VisCap output
- Visual assessment will act as an additional filter for the removal of false positives

Description of the VisCap Output

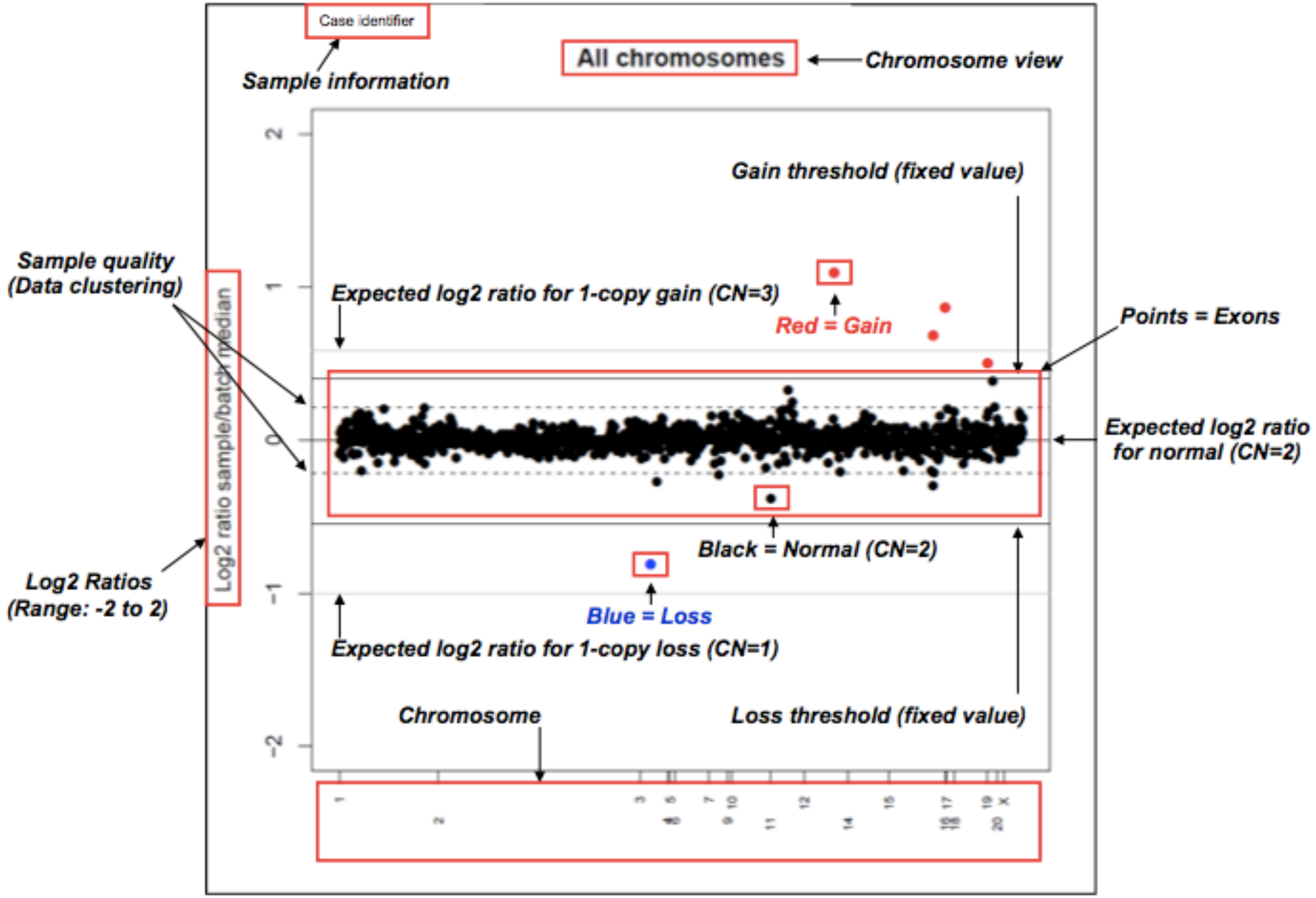

Description of the VisCap Output

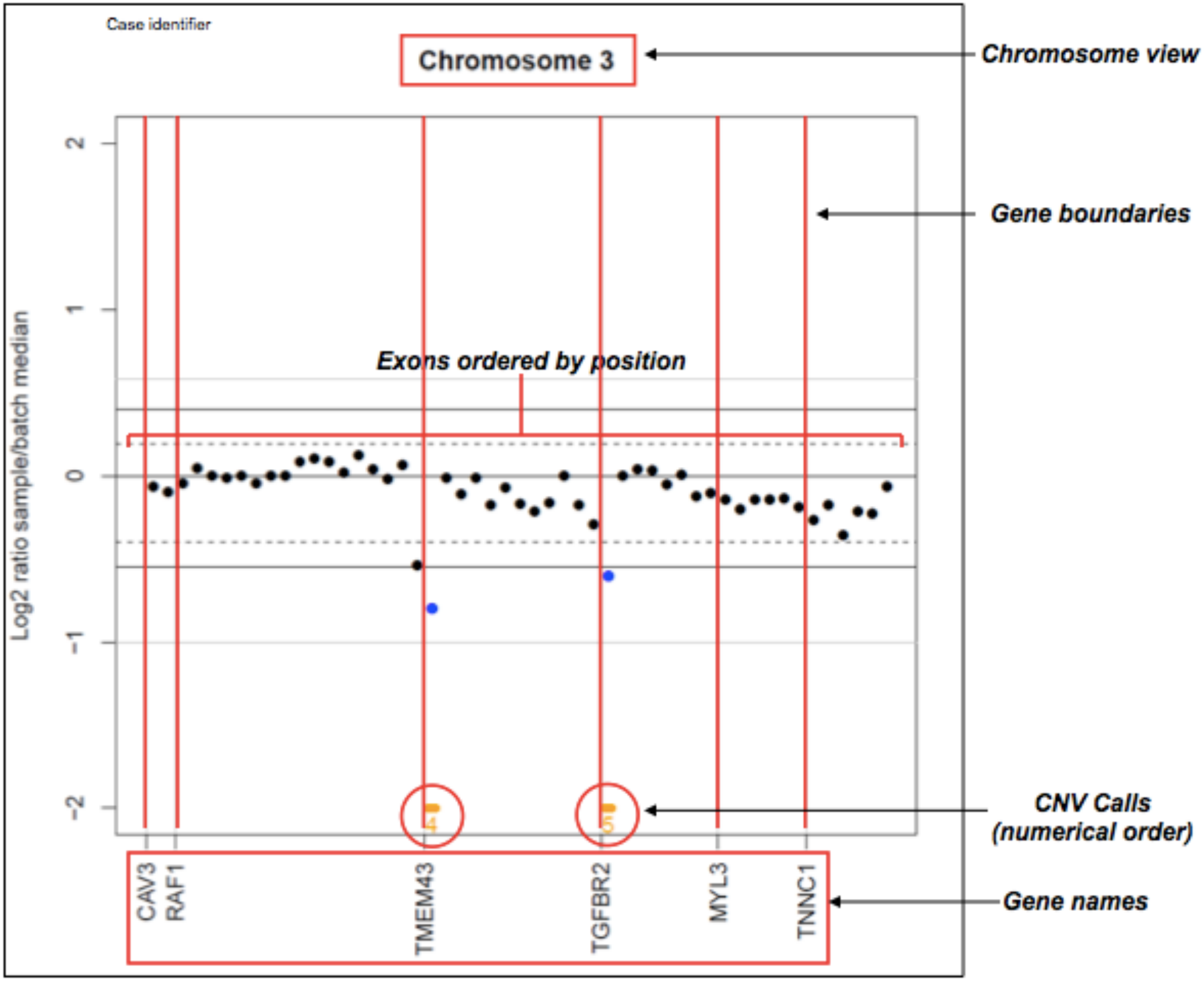

Visual Assessment Steps

Step #1 **Review page #1 (all chromosomes view) of VisCap output**

<u>Questions</u>

Overall data quality?
Where do the majority of points cluster?
What is the overall pattern of the data points?
How many CNVs are called?
Are there obvious CNVs based on expected log2 ratios?

Step #2 **Go to chromosome of interest to review CNV calls**

<u>Questions</u>

Overall data quality for chromosome and/or gene with called CNV?
What is the pattern of other points flanking the called CNV within the same gene?
How close are the called data points to the gain/loss thresholds?
If consecutive exons are called, how do they compare to one another?
How do the called exons compare to expected log2 ratios?

Visual Assessment Step #1 Examples

Step #1: Reviewing Page #1 (All chromosomes)

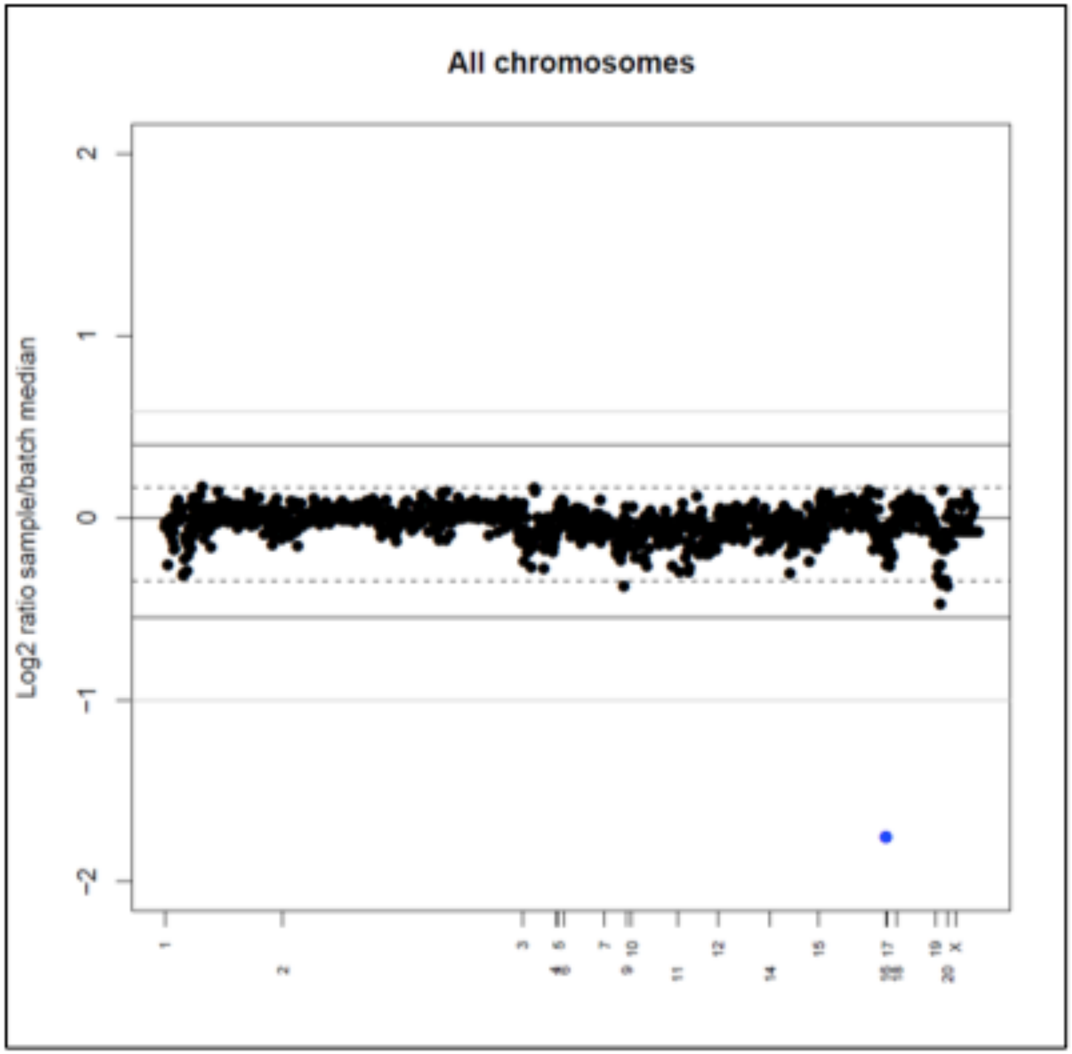

Overall data quality?
Data points are well clustered with little scattering observed
OK!

Where do the majority of points cluster?
Majority of points cluster around log2 ratio = 0, which indicates proper normalization of the sample
OK!

What is the overall pattern of data points?
Some increased scattering across regions, but the overall pattern is level
OK!

How many CNVs are called?
Only one CNV observed
OK!

Are there points close to (or beyond) expected log2 ratios for gains/losses?
Yes
OK!

Step #1 Assessment: **OK to proceed with visual assessment at single chromosome level**

Visual Assessment Step #1 Examples

Step #1: Reviewing Page #1 (All chromosomes)

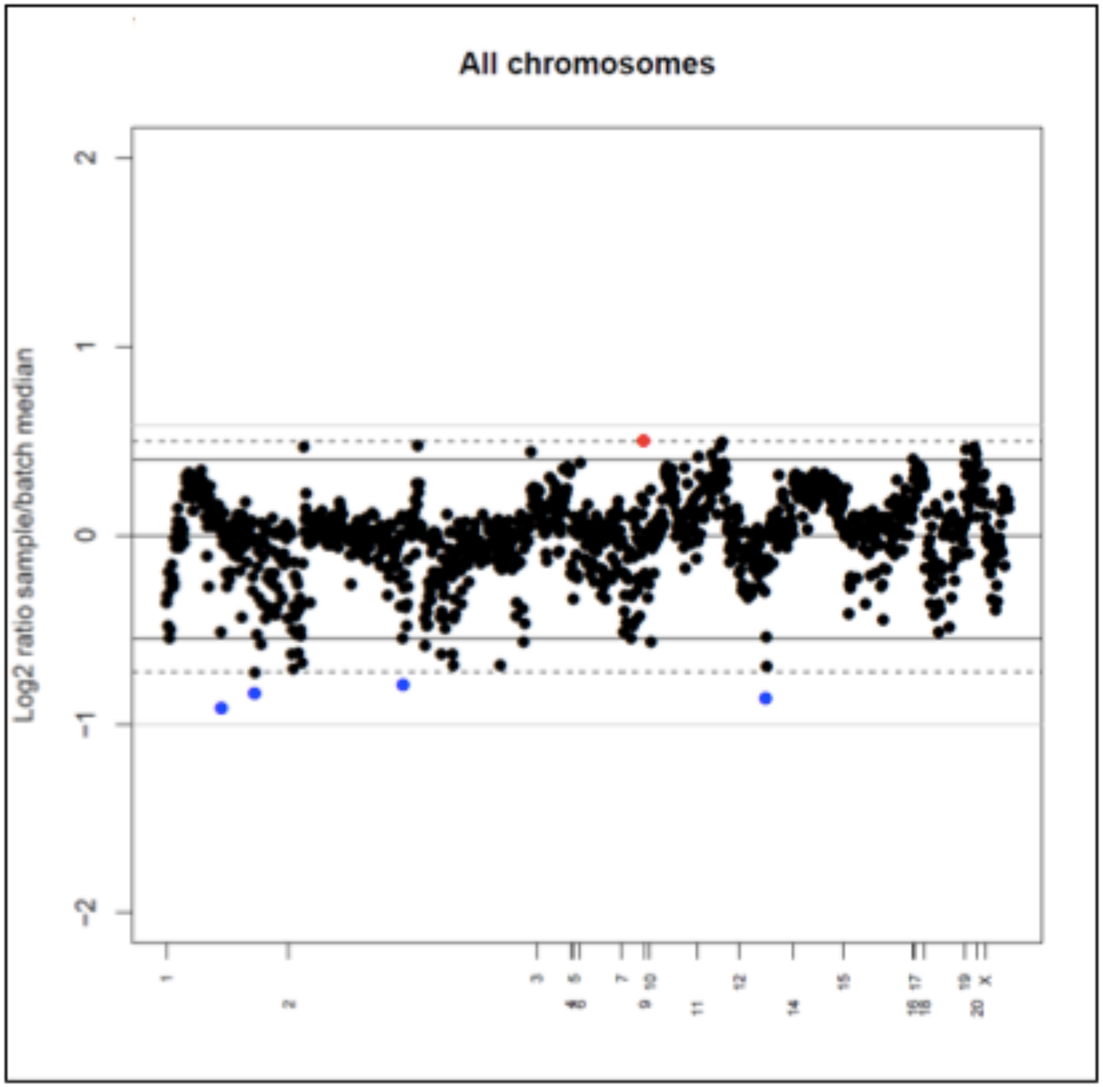

Overall data quality?
Data points show large amount of scattering
PROBLEM!

Where do the majority of points cluster?
Cannot tell if sample normalization is OK due to scattering
PROBLEM!

What is the overall pattern of data points?
Large amount of scattering across all regions with up & down pattern (not level)
PROBLEM!

How many CNVs are called?
Only 5 CNVs are called, but that is due to wide gain/loss thresholds
PROBLEM!

Are there points close to (or beyond) expected log2 ratios for gains/losses?
Yes, but unclear if these are true CNVs due to bad data quality (need to look at specific regions)
PROBLEM!

Step #1 Assessment: **Proceed with assessment at single chromosome level, but make note of poor sample quality**

Visual Assessment Step #2 Examples

Step #2: Reviewing Called CNV (Single chromosome view)

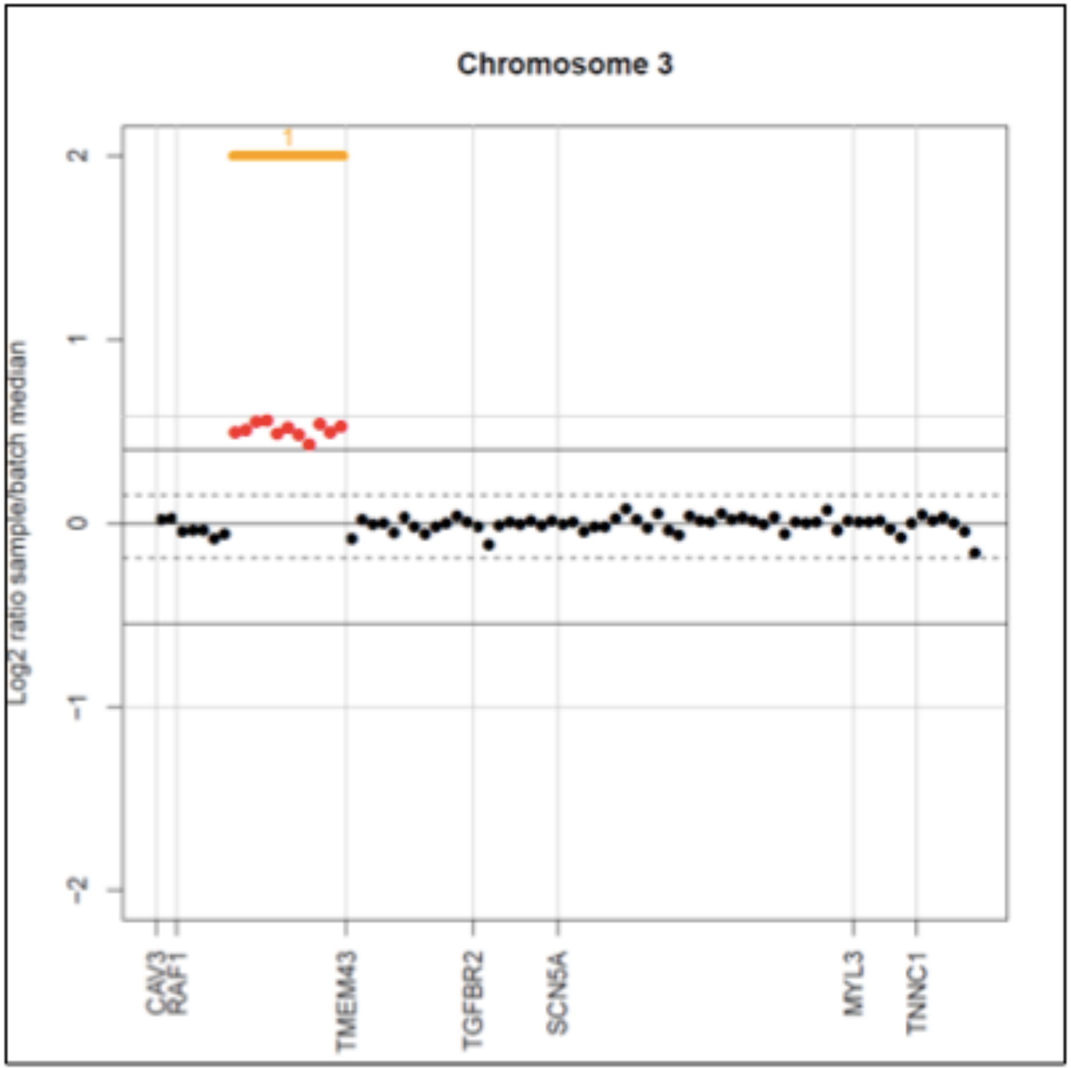

Overall data quality?
Data points are well clustered across chromosome with little scattering observed
OK!

What is the pattern of data points flanking the called CNV in the same gene?
All flanking data points cluster around log2 ratio = 0
OK!

How close are the called exons to the gain/loss thresholds?
All called exons are above the gain threshold
OK!

If CNV consists of consecutive exons, how do they compare to one another?
All called exons have very similar log2 ratios
OK!

How close are called exons to expected log2 ratios for gain/losses?
All called exons are close to a log2 ratio = 0.58 (light gray line), which suggests a 1-copy gain
OK!

Step #2 Assessment: **This CNV appears to be real and should receive additional evaluation and/or confirmation**

Visual Assessment Step #2 Examples

Step #2: Reviewing Called CNV (Single chromosome view)

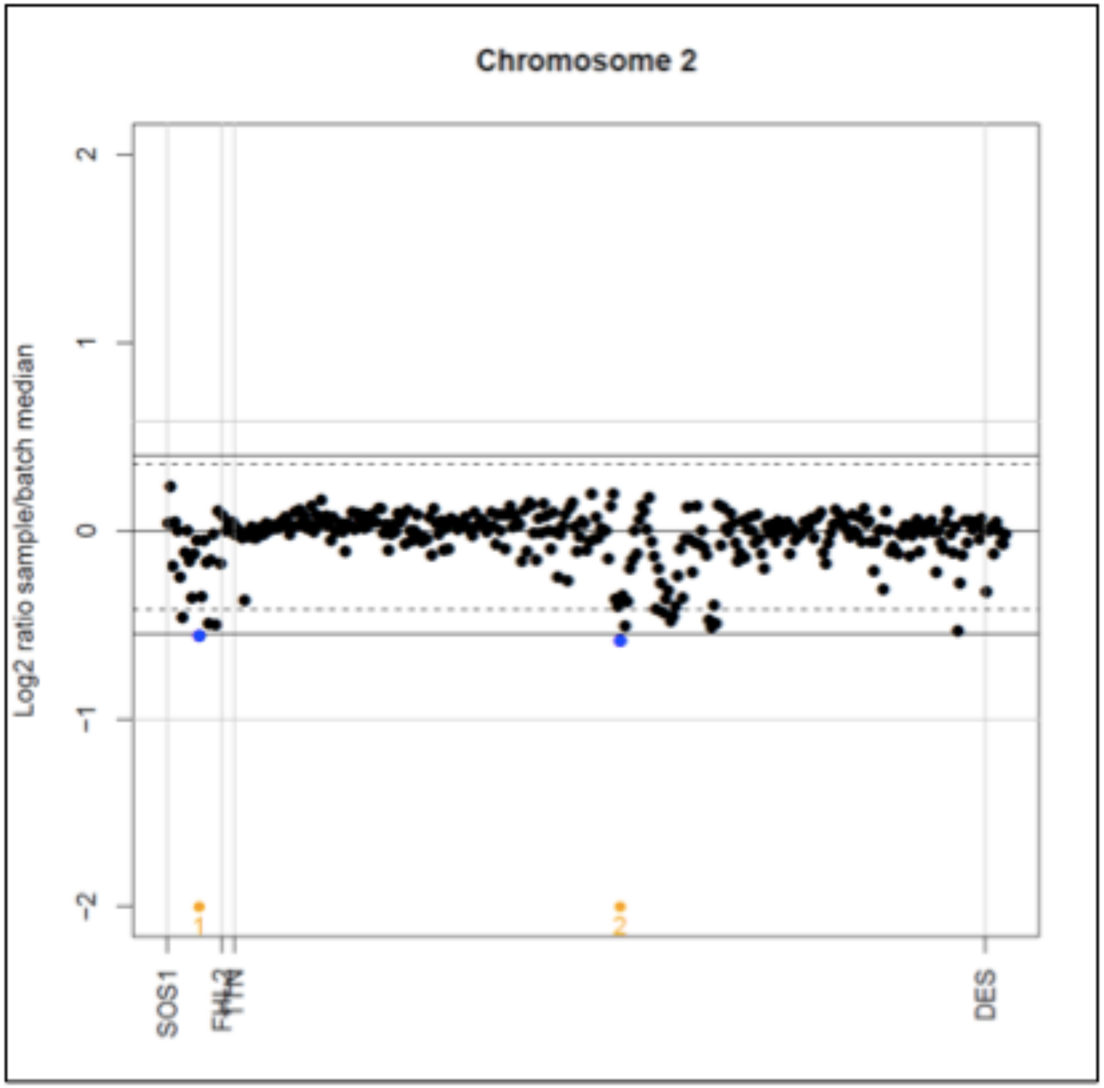

Overall data quality?
The majority of data points are well clustered in the chromosome with some scattering among different regions
OK!

What is the pattern of data points flanking the called CNV in the same gene?
Called exons are among a string of exons gradually sloping downward below loss threshold
PROBLEM!

How close are the called exons to the gain/loss thresholds?
Both called exons are only slightly below the loss threshold
PROBLEM!

If consecutive exons are called, how do they compare to one another?
No consecutive exon CNVs observed

How close are called exons to expected log2 ratios for gain/losses?
None of the called exons approach the expected log2 ratio for a true 1-copy loss
PROBLEM!

Step #2 Assessment: **These CNVs appear to be a false positives (not recommended for follow-up)**

Recognizing the Patterns

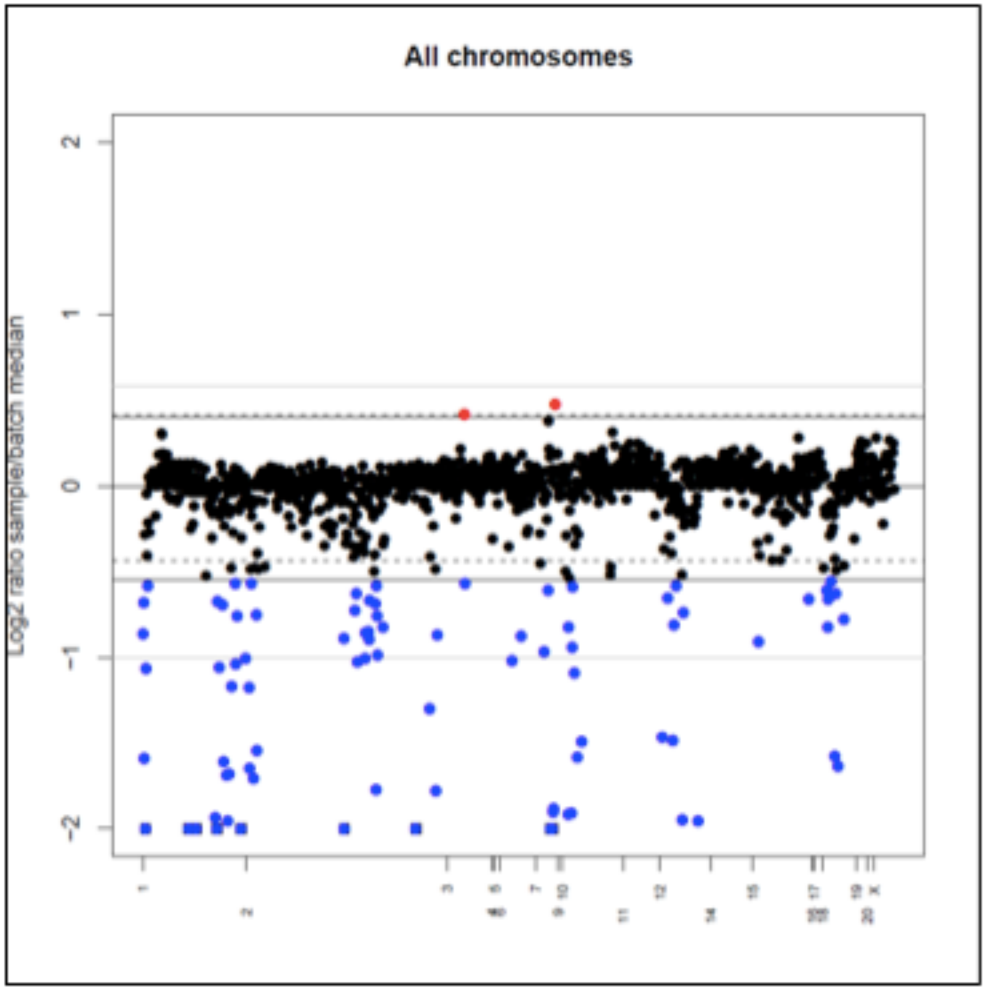

"Waterfall"

- Numerous losses called
- No clustering of consecutive exons around a similar log2 ratio
- Called exon log2 ratios spread out through range of -0.55 to -2
- These are frequently **FALSE POSITIVES**

Recognizing the Patterns

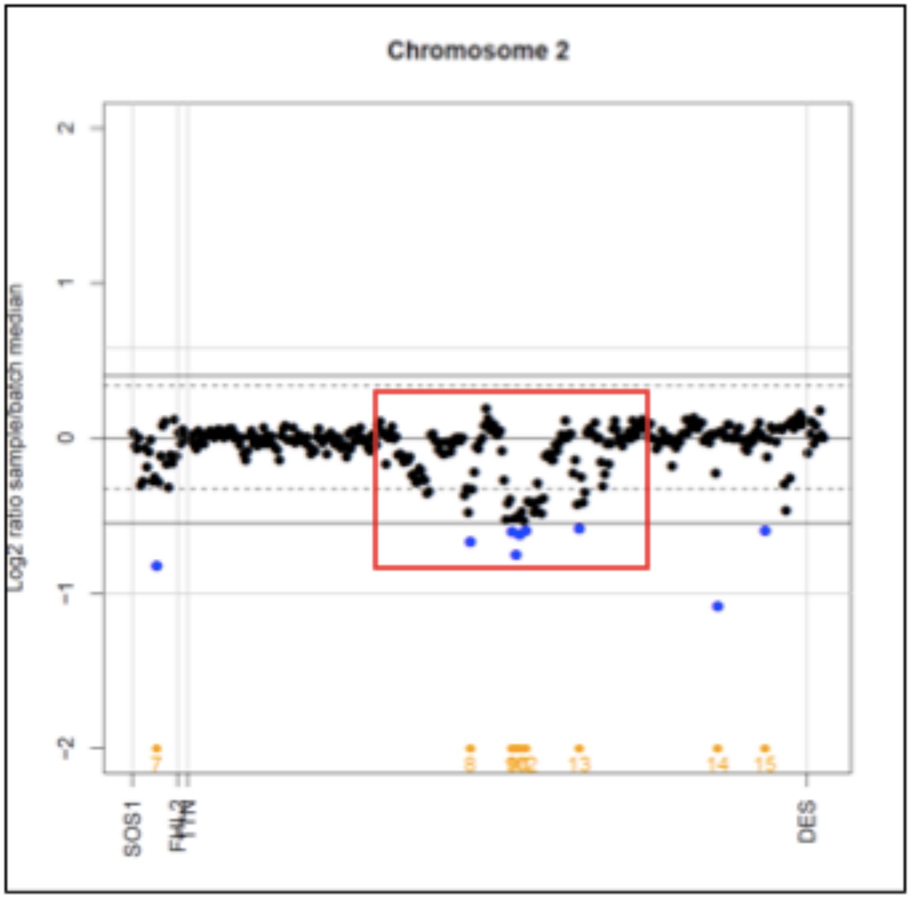

“Hills” & “Valleys”

- Called exons are part of steadily increasing or decreasing series of exons
- Called exons rise or fall slightly beyond gain/loss thresholds
- Usually do not approach expected log2 ratio values
- Consecutively called exons often have dissimilar log2 ratios
- Often seen within TTN gene on Chr2 (cardio panel) and ChrX (all panels)
- These are frequently **FALSE POSITIVES**

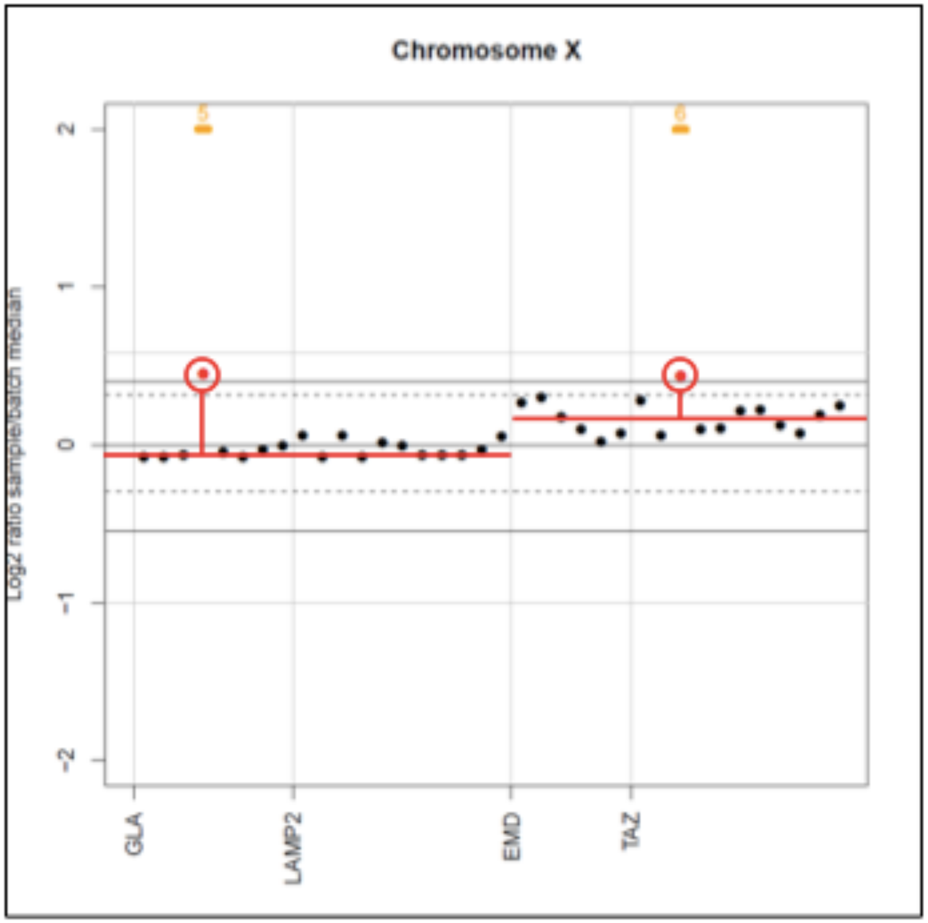

Entire Region Shifted Up/Down

- All exons of gene(s) shifted above or below baseline
- Called exons do not approach expected values for gain/loss
- Called exons are close to thresholds
- Mostly seen on the ChrX (all panels)
- Most often due to low-quality sample in batch or imbalance in male/female
- These are frequently **FALSE POSITIVES**

Recognizing the Patterns

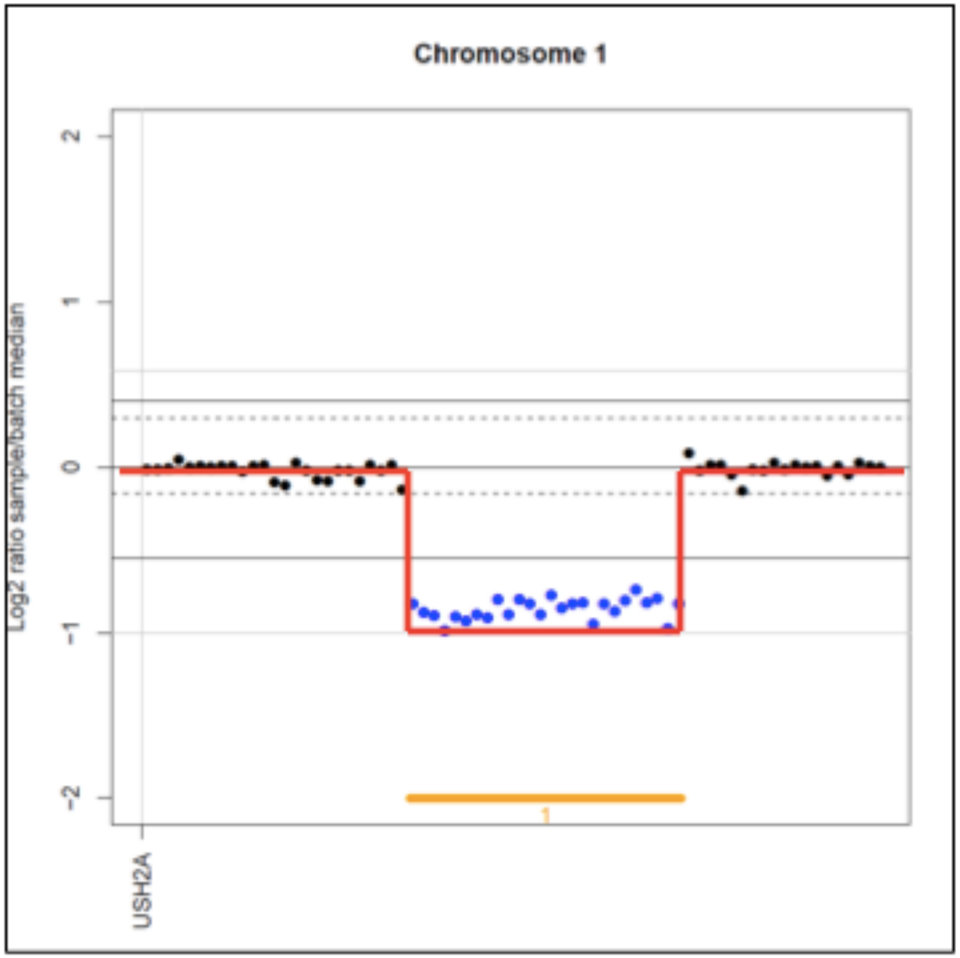

"Cliffs" & "Plateaus"

- Flanking uncalled exons are tightly clustered around log2 ratio = 0
- Called exons are far removed from flanking uncalled exons
- All consecutively called exons have similar log2 ratios
- Called exons are far beyond thresholds
- These are **TRUE POSITIVES**

Assessing CNVs spanning multiple genes

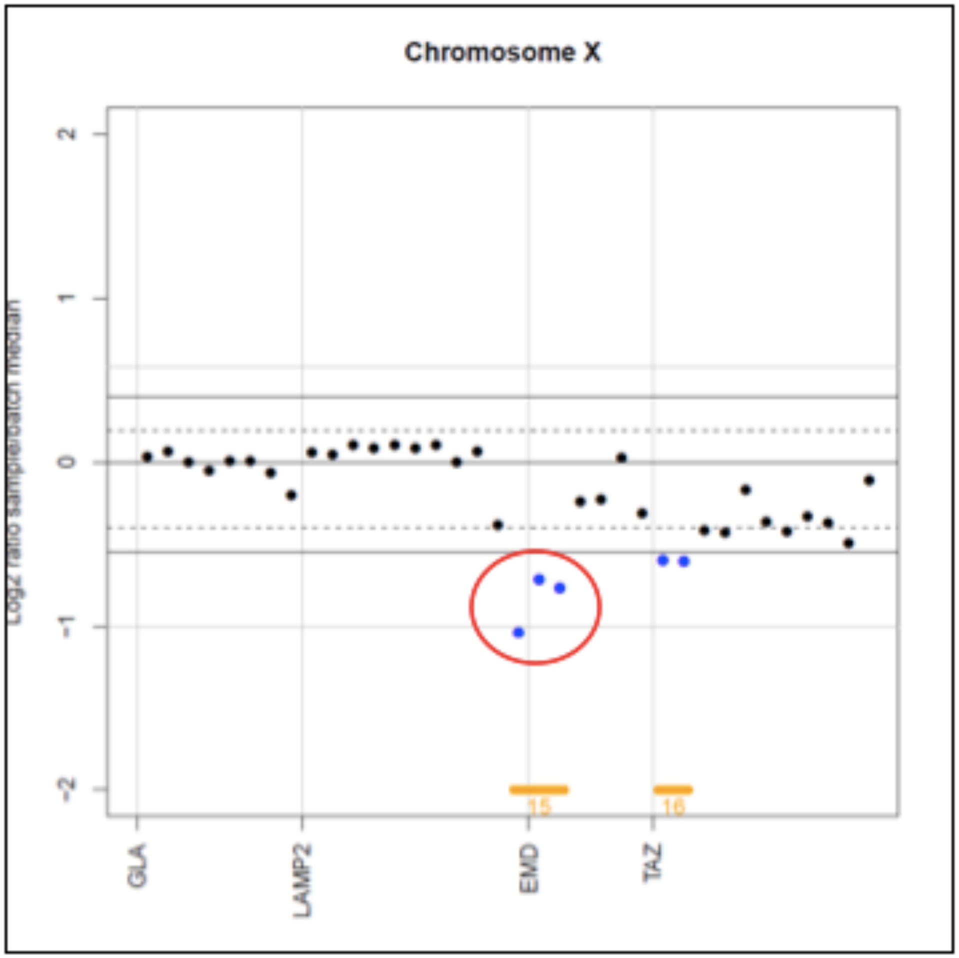

<u>PROBLEM</u>:
Part of CNV in Gene #1 appears real, but not the other exons in Gene #2
- If any portion of CNV appears to be real, suggest follow-up
- Specify on score sheet which exons appear real
- After NVA, decision will be made on which exon to assay by ddPCR

Using flanking exons to assess called CNV

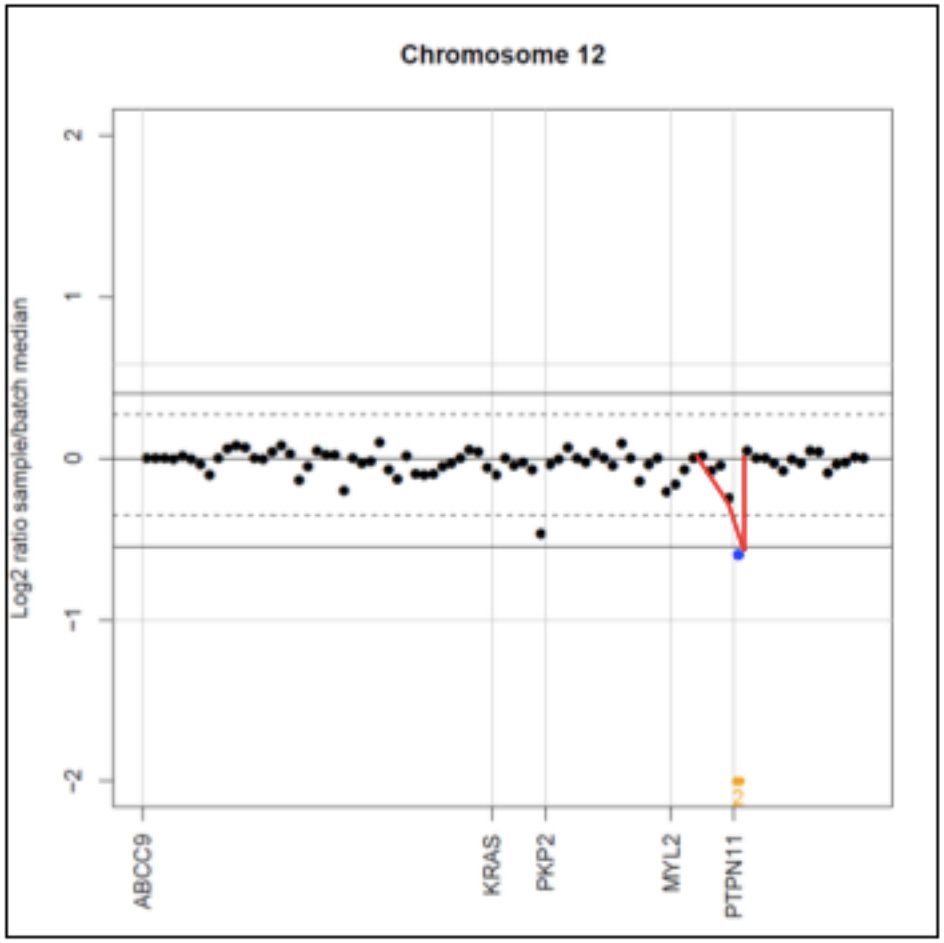

<u>PROBLEM</u>:
Exons flanking CNV on one side appear to show characteristic sloping effect often seen in false positive calls, but exons are in different gene

- Although the exons appear to be side-by-side in VisCap output, the physical distance between the genes could be quite large
- Should <u>not</u> make assumptions on data quality between such distantly positioned regions
- Only flanking exons <u>within same gene</u> of CNV call should be considered

Examples of False Positive Patterns

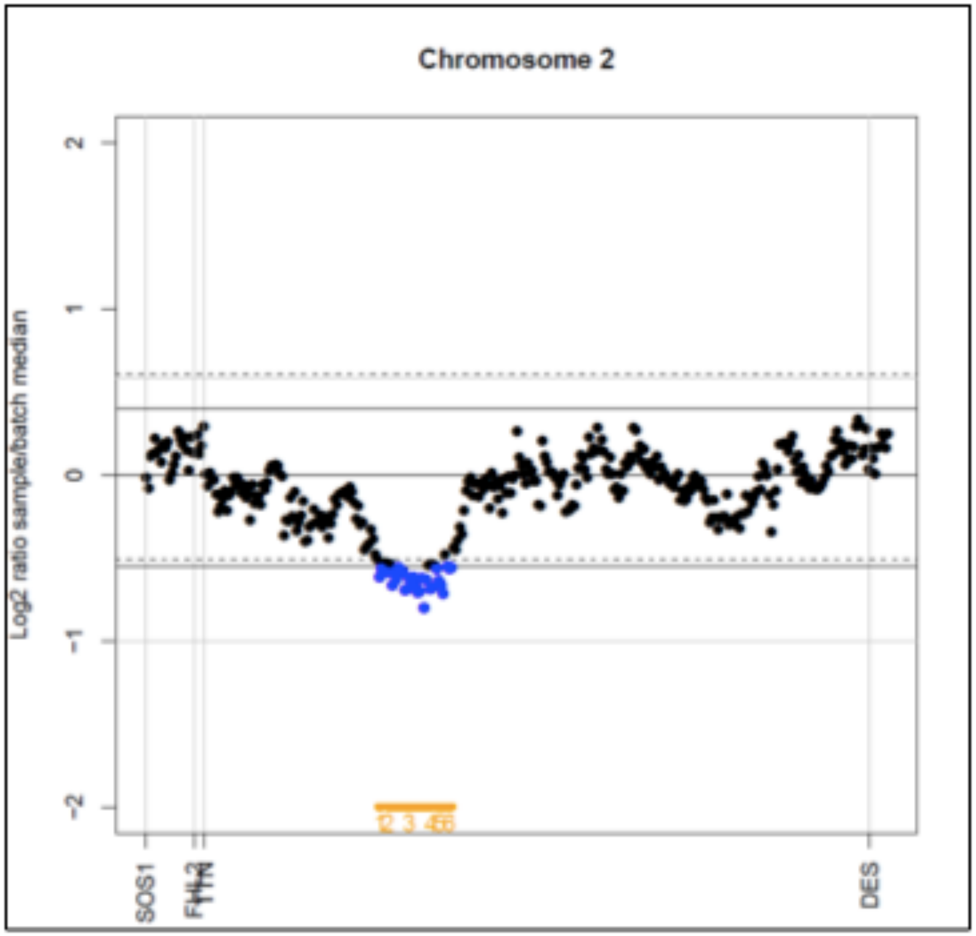

<u>PROBLEM</u>:

Data points show characteristic “hills and valleys” pattern.

- Although multiple consecutive exons are called, this is an obvious false positive
- There is poor separation between the called CNV and the flanking exons
- The called exons are not close the expected value of -1 for a 1-copy loss

Identifying Possible CNVs in Failed Samples

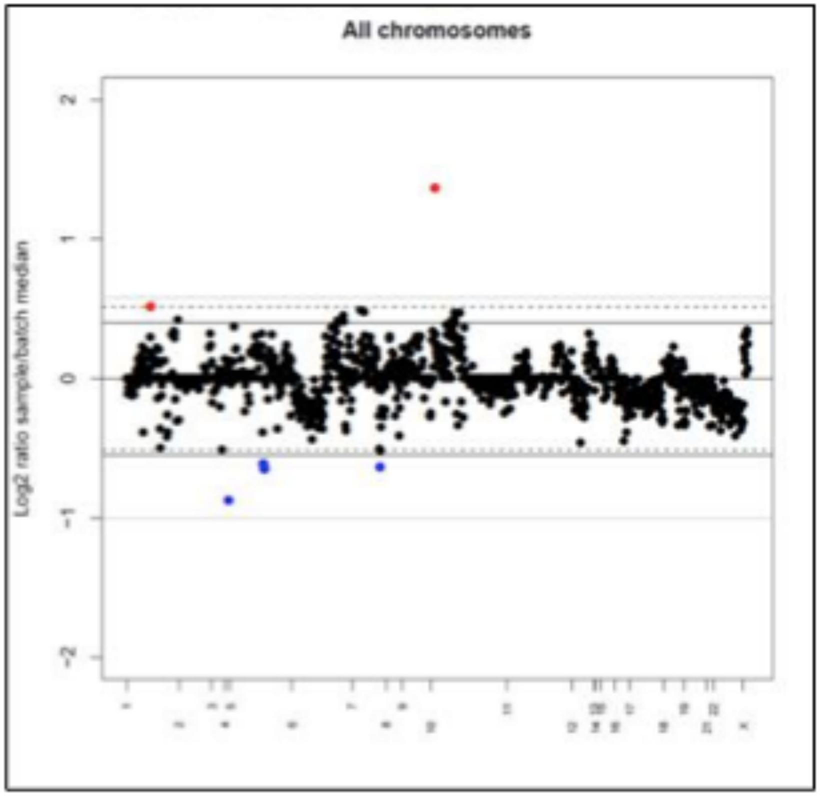

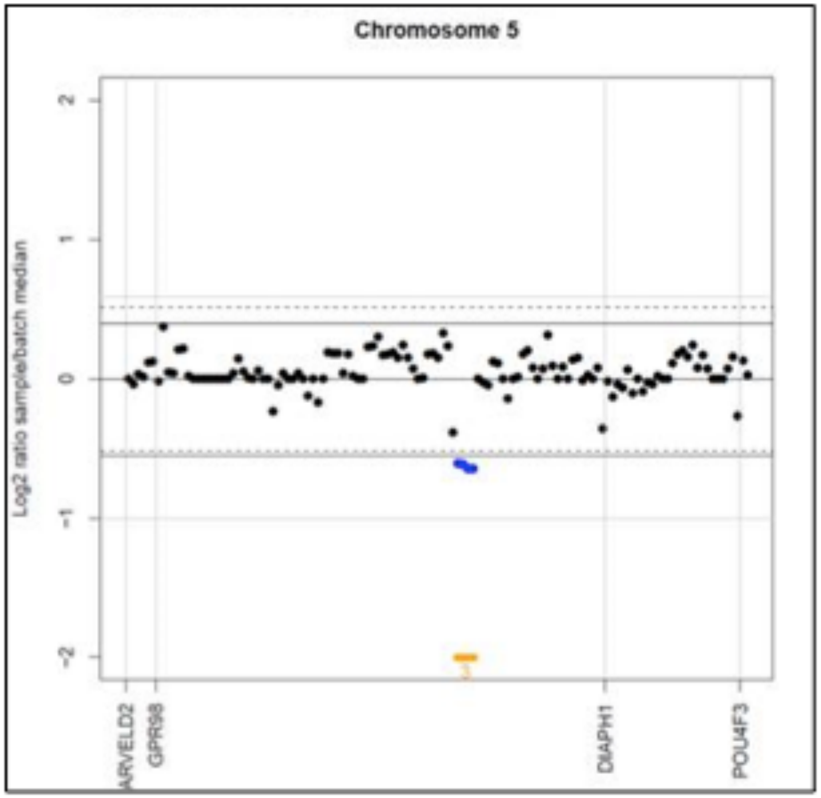

<u>PROBLEM</u>:
Sample passes VisCap QC, but reviewer decides to fail sample based on visual review. After quickly scanning the results, a 4 consecutive exon loss is observed.

- Normally, there is low confidence in any CNV call found in failed samples (no follow-up)
- In this case, the gene-level quality is good in GPR98, and a clean multi-exon call is seen
- Since we have much higher confidence in mult-exon calls within genes of good data quality, this would be situation where the reviewer should notify the rest of the team

Important Things to Remember:

- Review “All Chromosomes” (Page #1) to assess overall sample quality
- Pay attention to the patterns observed in the data (What is the trend?)
- Always compare the called exons to the expected log2 ratios
- If questionable, it’s better to include for follow-up than to remove
