# Supplementary material for: VisCap: inference and visualization of germ-line copy-number variants from targeted clinical sequencing data
Source: Genet Med. 2015 Dec 17;18(7):712–9. doi: 10.1038/gim.2015.156 (PMC4940431; doi:10.1038/gim.2015.156)
Supplement: Supplementary Information [file gim2015156x1.zip › Supplementary_File_3.zip!/Supplementary_File_2/Case_19.pdf]

Case 19

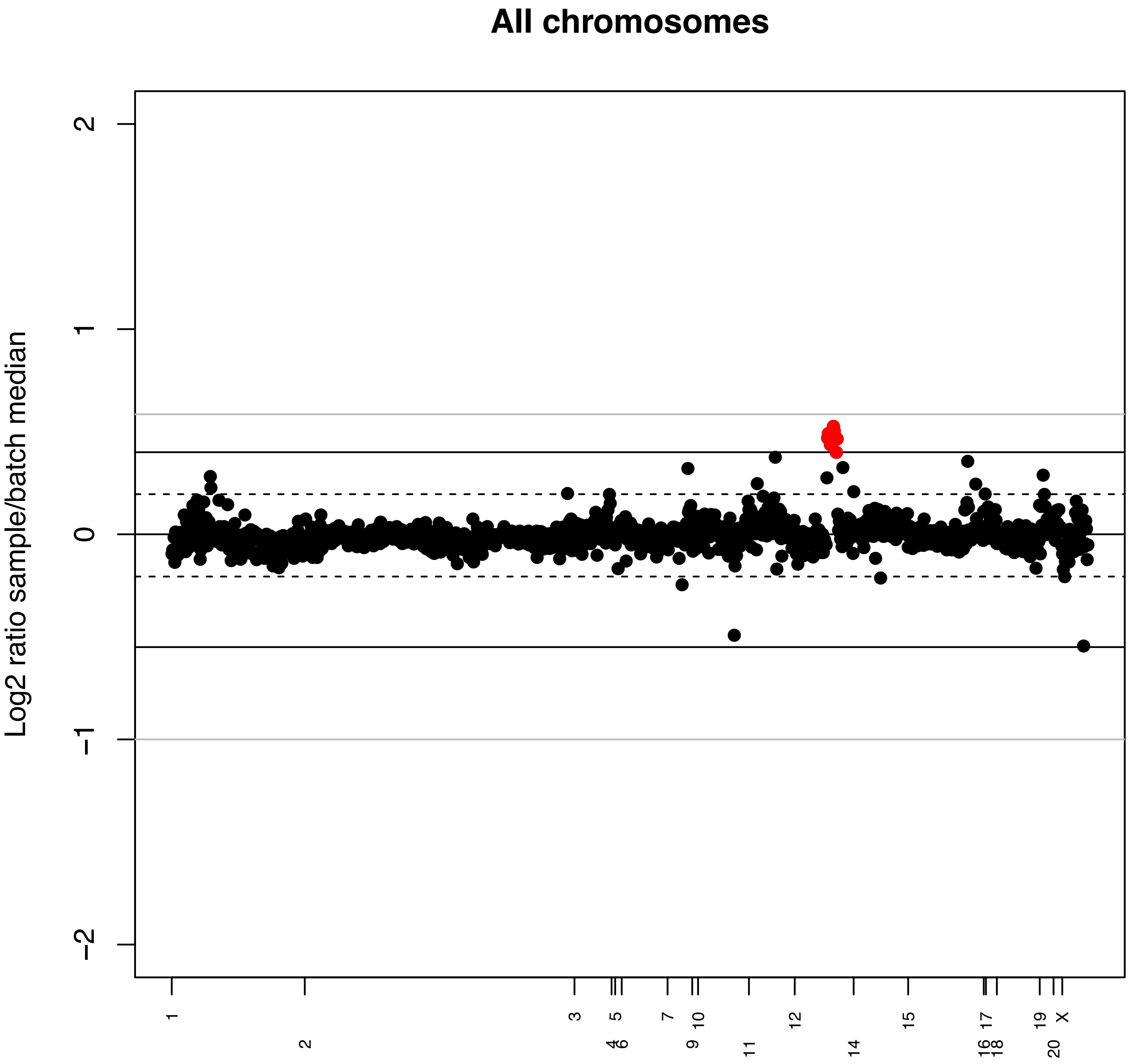

All chromosomes
Log2 ratio sample/batch median
2
1
0
−1
−2
1
2
3
4
5
6
7
9
10
11
12
14
15
16
17
18
19
20
X

Case 19

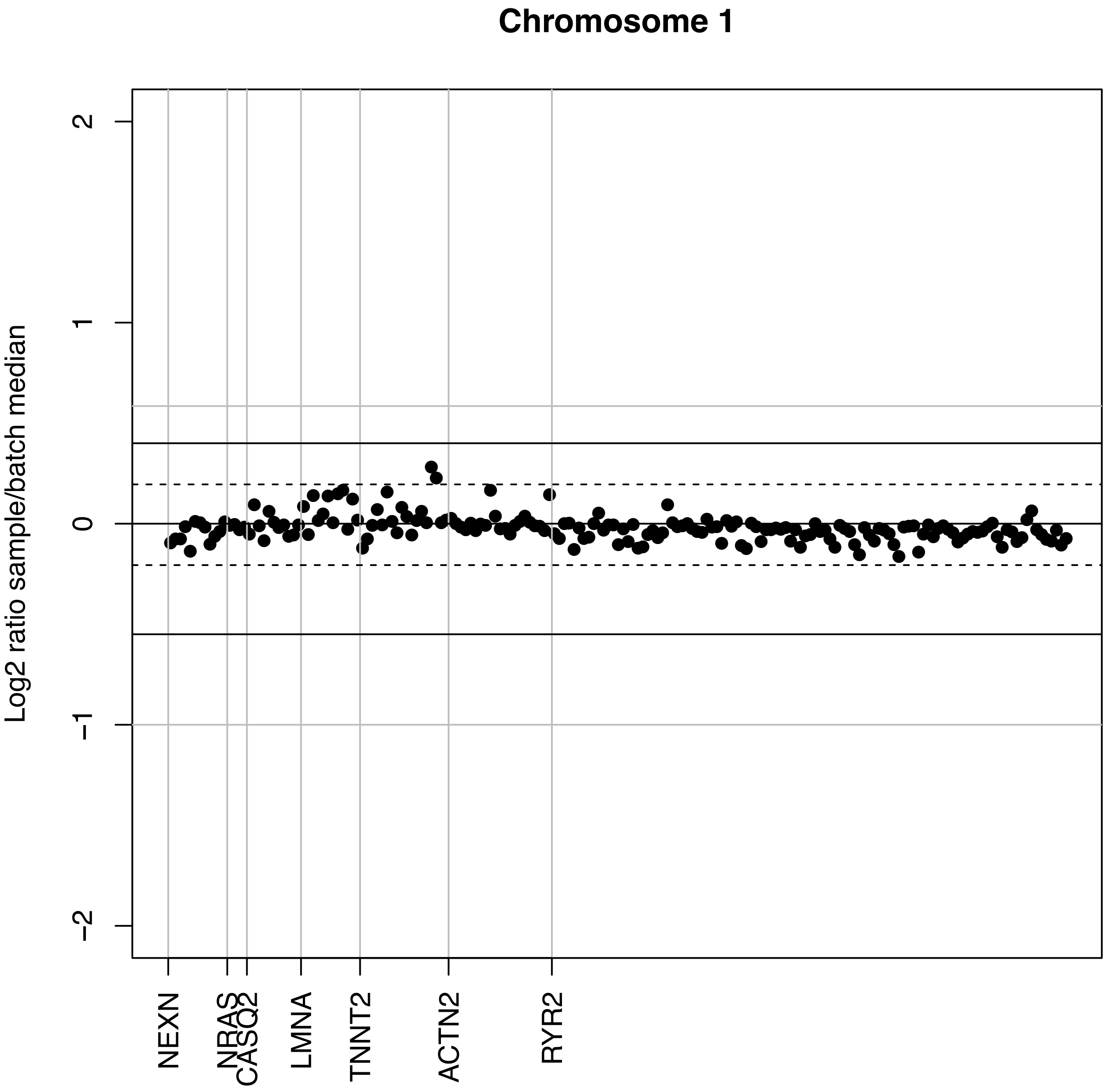

Chromosome 1
Log2 ratio sample/batch median
2
1
0
−1
−2
NEXN
NRAS
CASQ2
LMNA
TNNT2
ACTN2
RYR2

Case 19

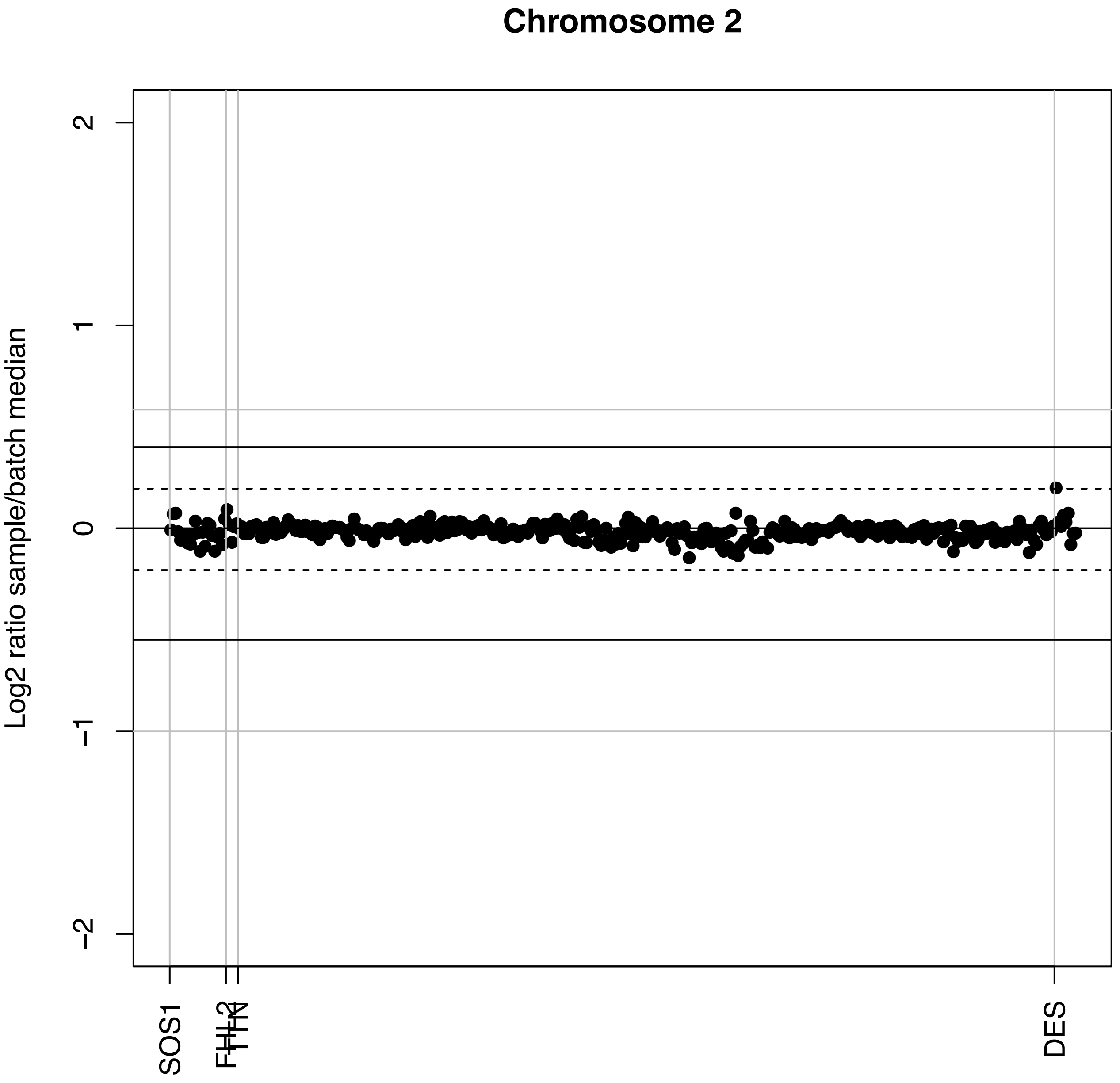

Chromosome 2
Log2 ratio sample/batch median
2
1
0
−1
−2
SOS1
FHL2
TTN
DES

Case 19

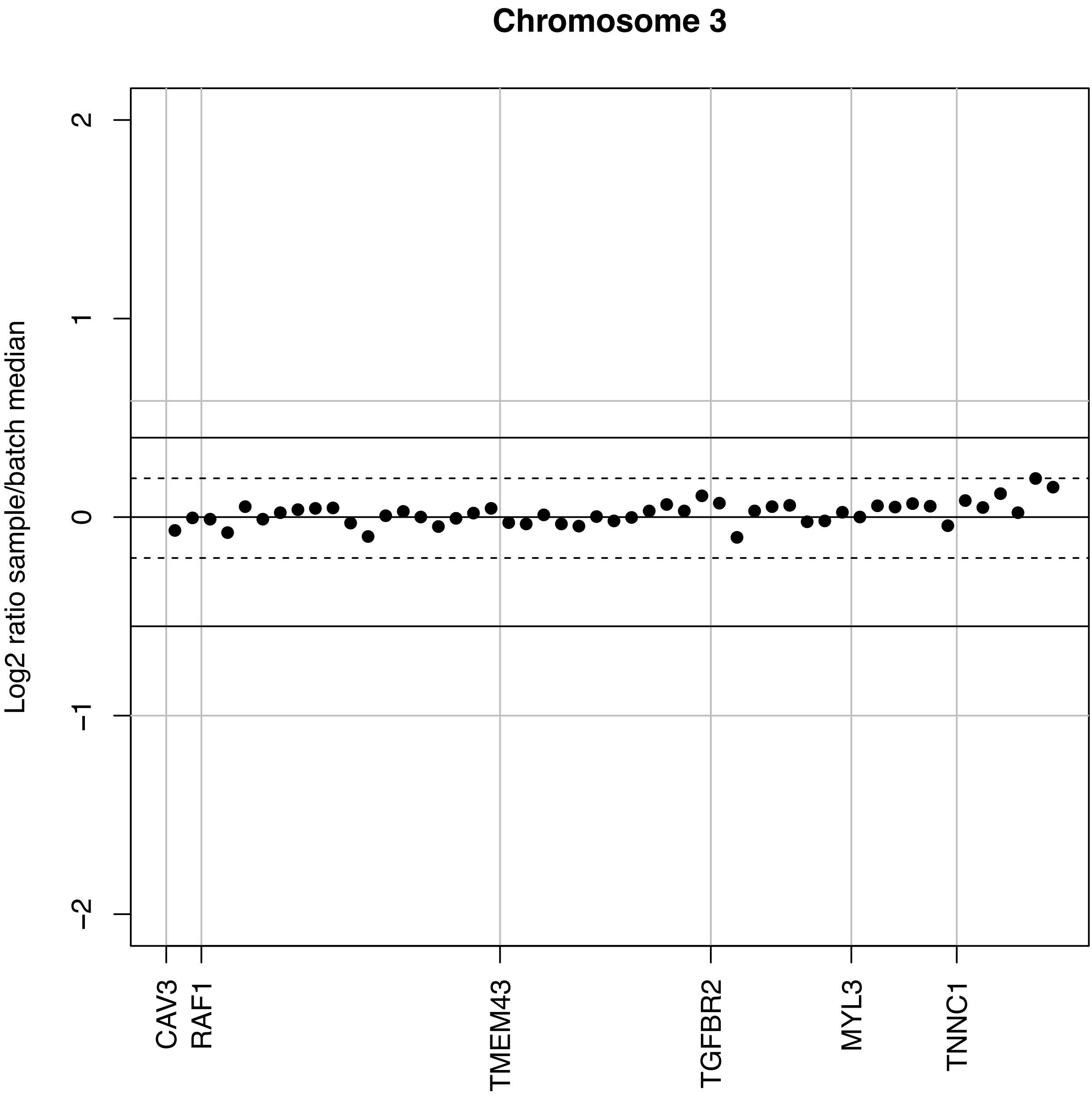

Chromosome 3
Log2 ratio sample/batch median
2
1
0
−1
−2
CAV3
RAF1
TMEM43
TGFBR2
MYL3
TNNC1

Case 19

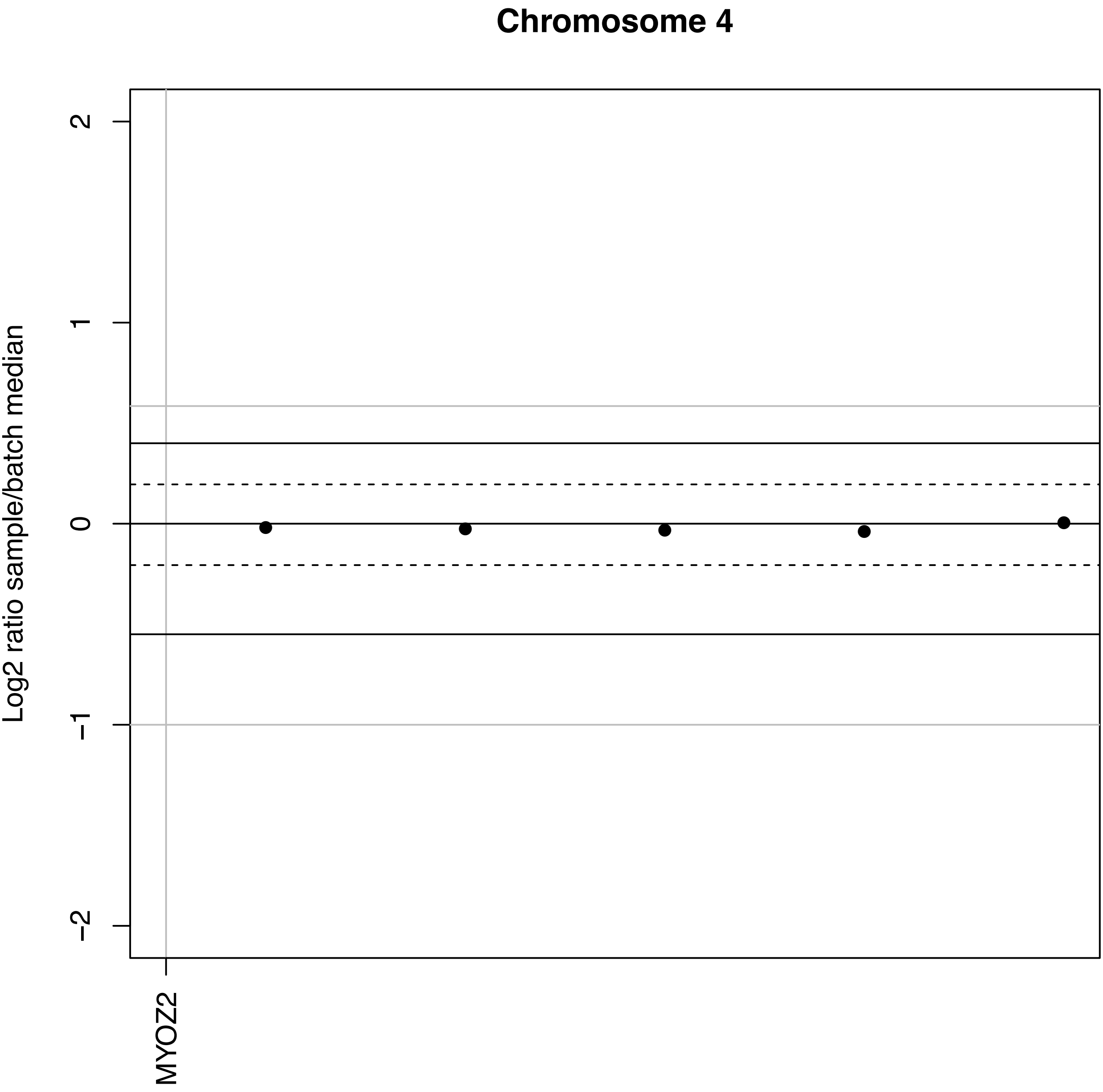

Case 19

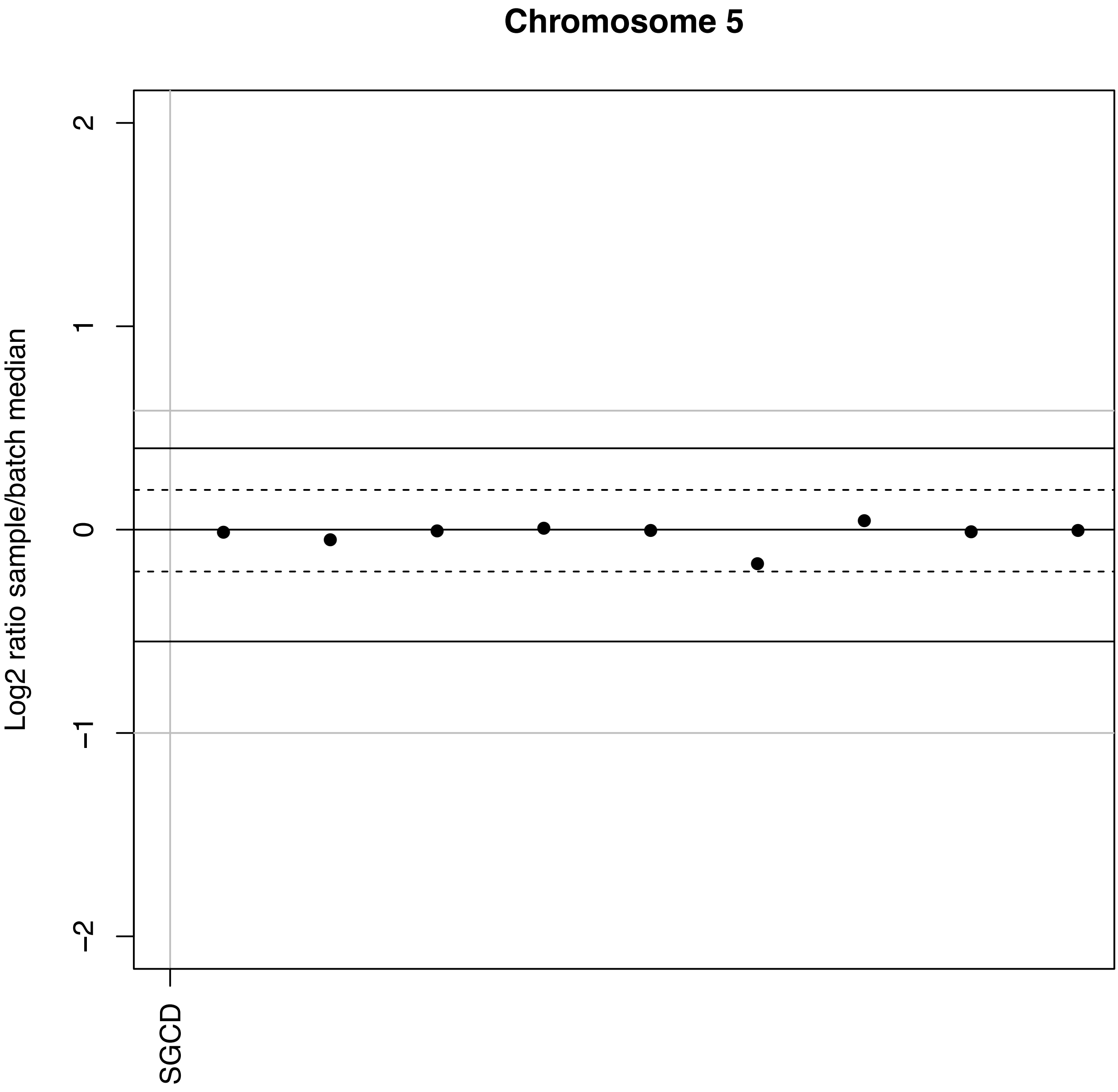

Case 19

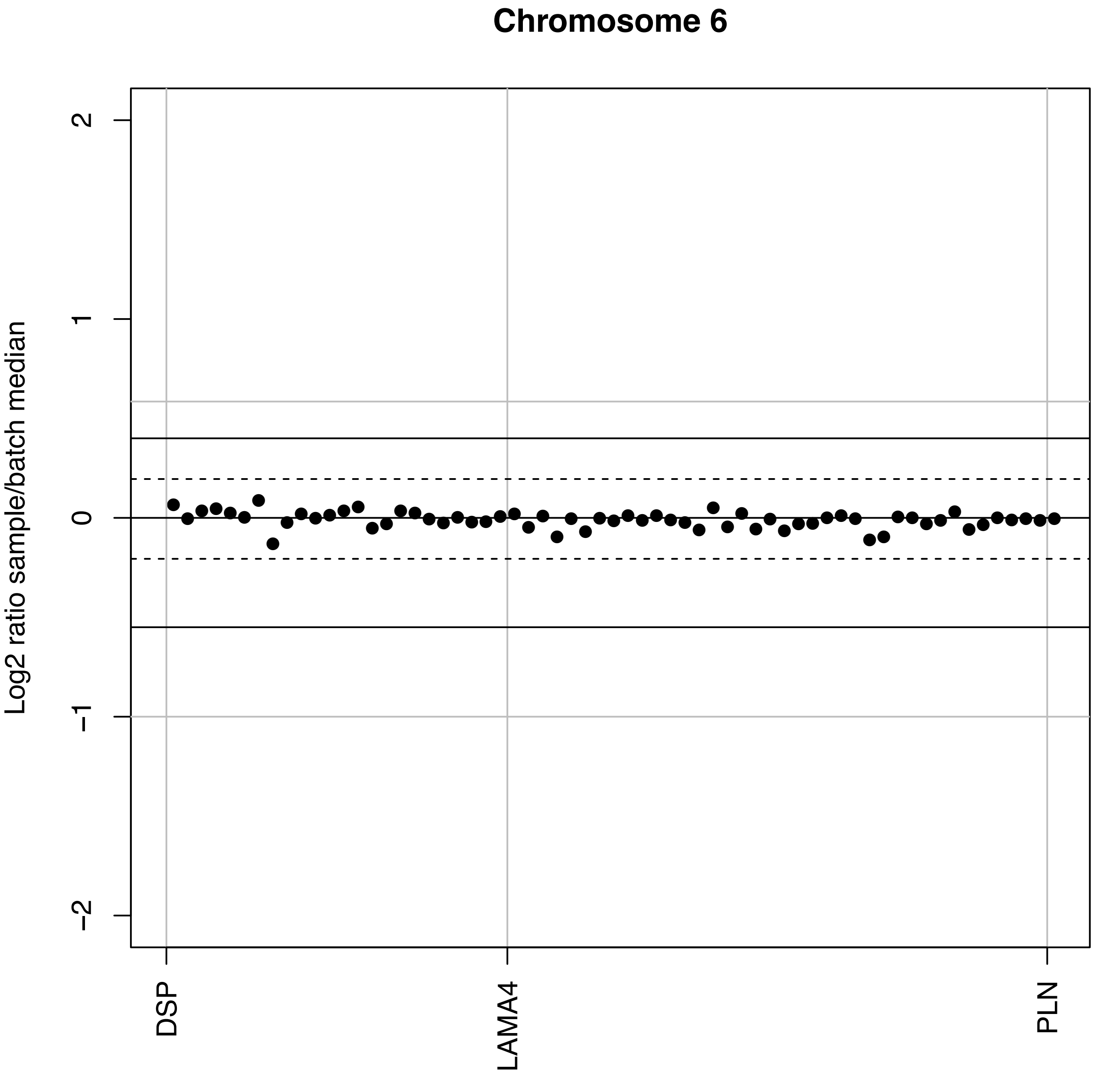

Chromosome 6
Log2 ratio sample/batch median
2
1
0
−1
−2
DSP
LAMA4
PLN

Case 19

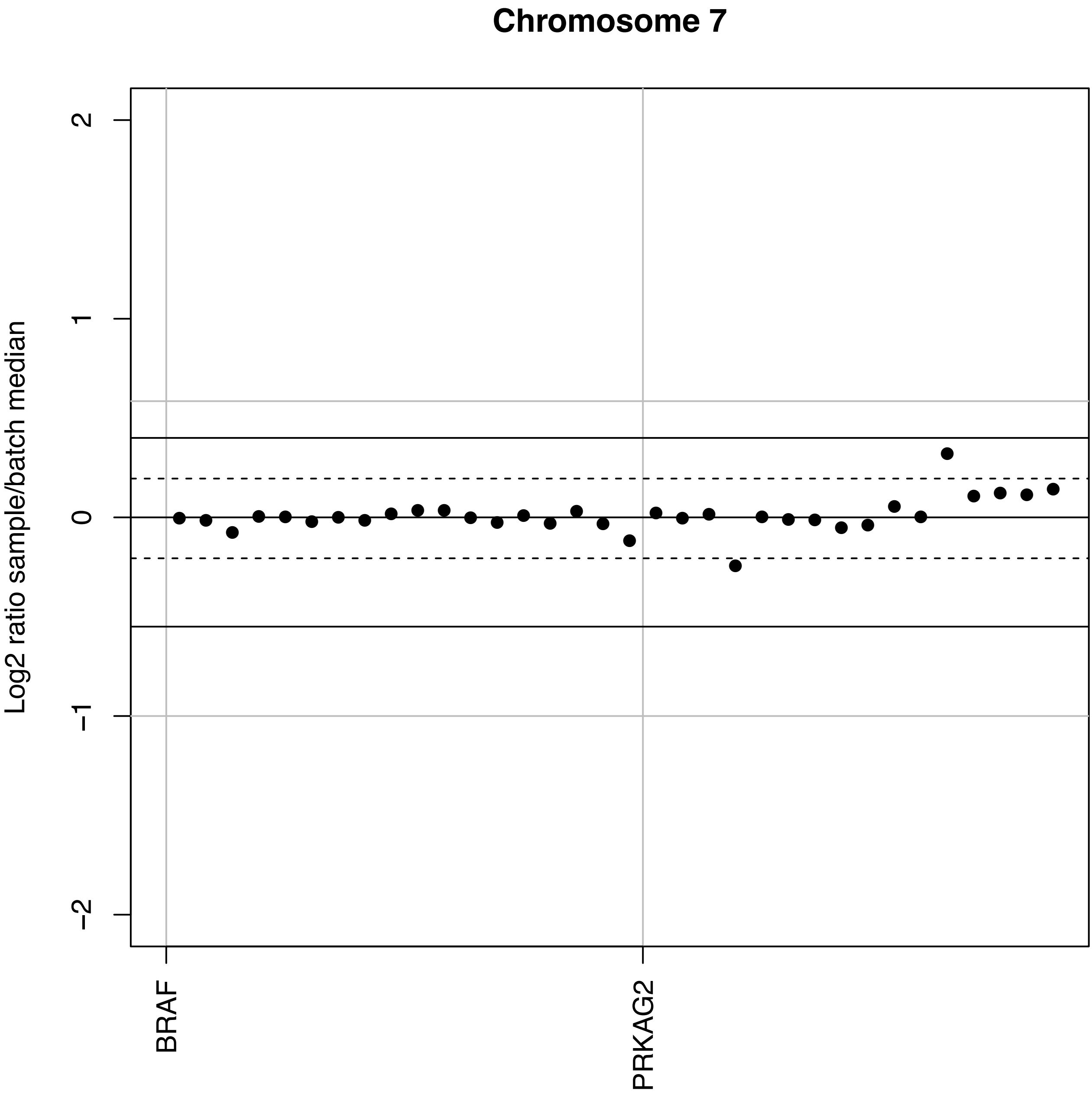
Chromosome 7
Log2 ratio sample/batch median
2
1
0
−1
−2
BRAF
PRKAG2

Case 19

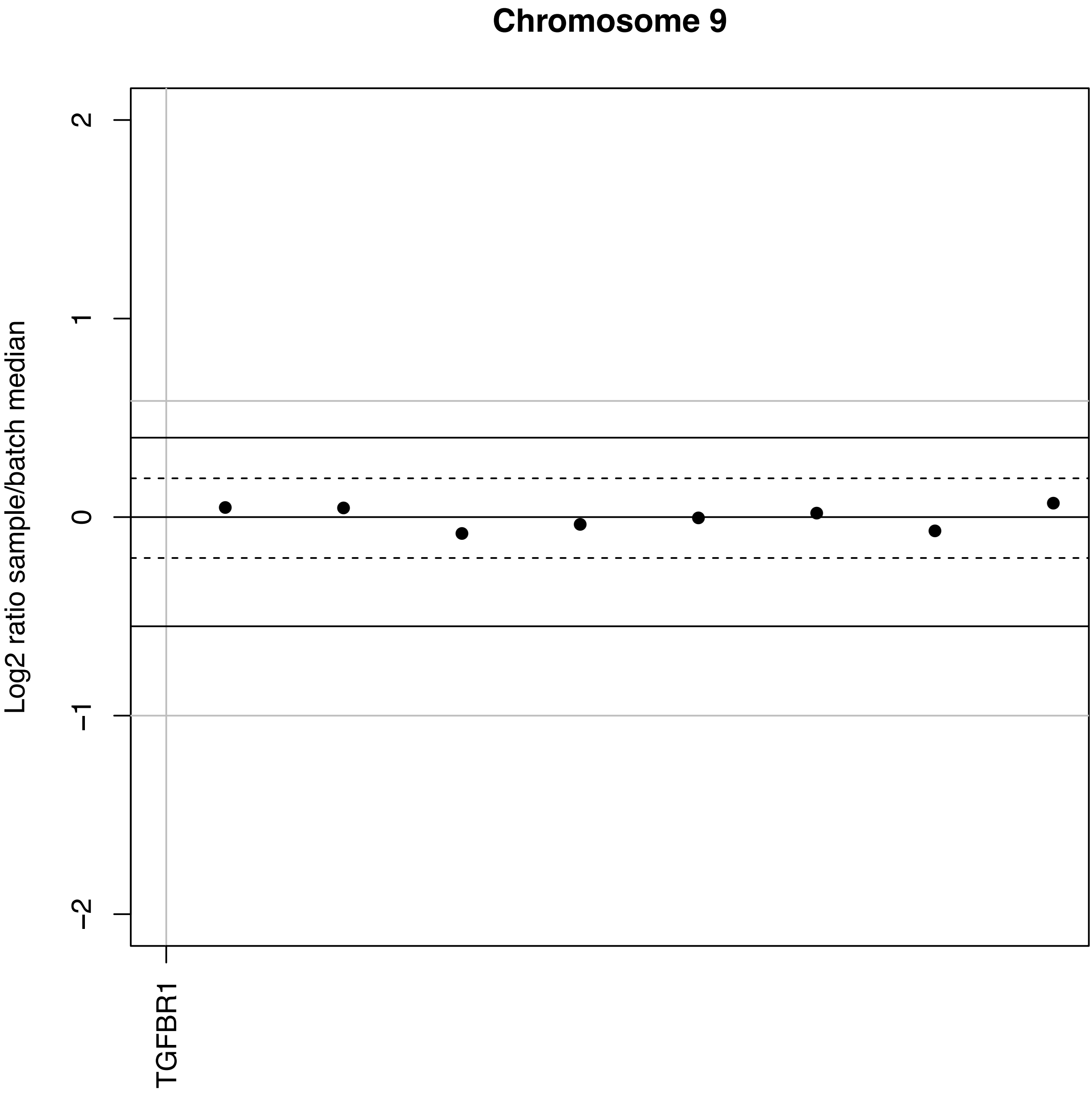

Case 19

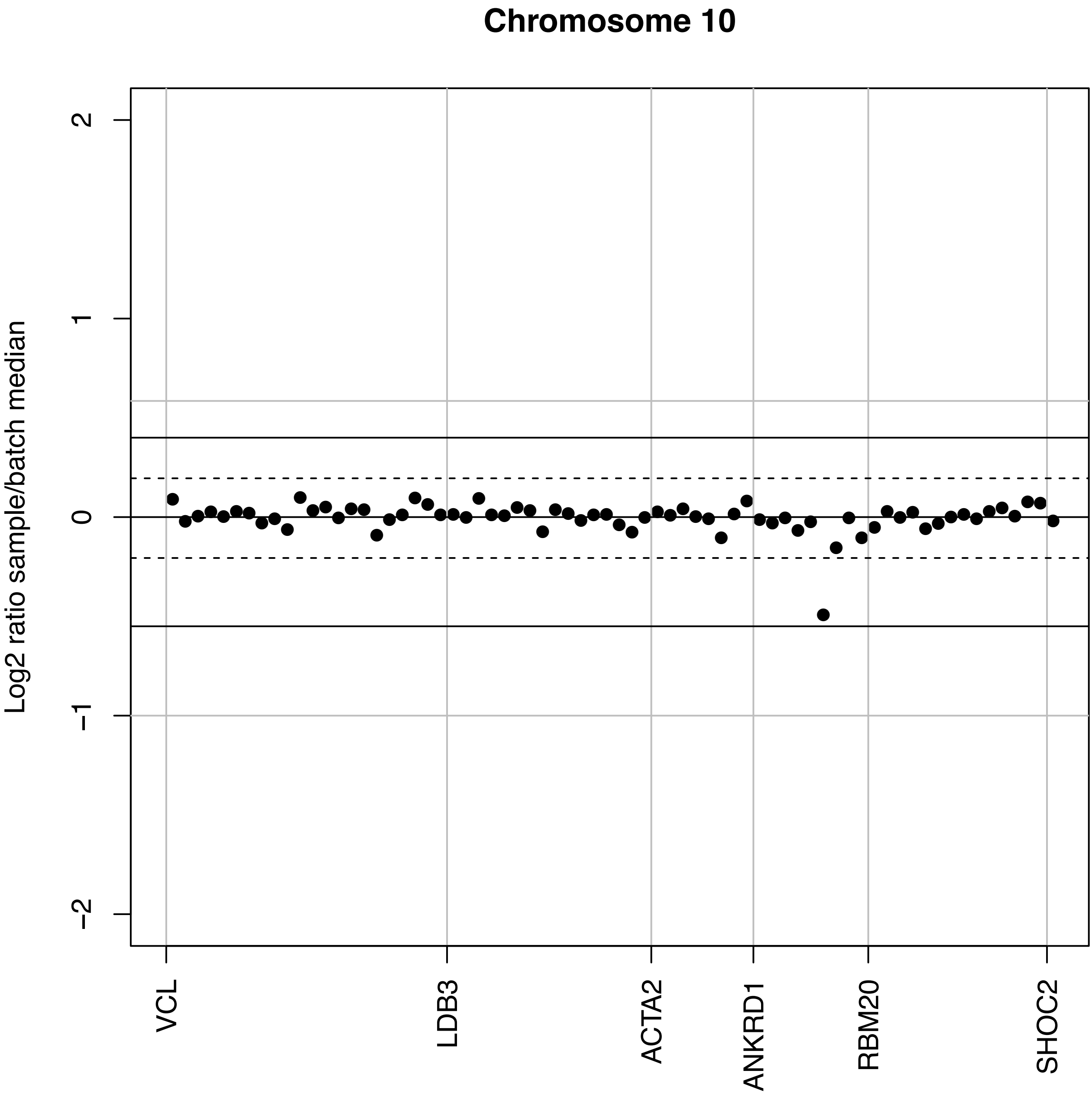

Chromosome 10
Log2 ratio sample/batch median
2
1
0
−1
−2
VCL
LDB3
ACTA2
ANKRD1
RBM20
SHOC2

Case 19

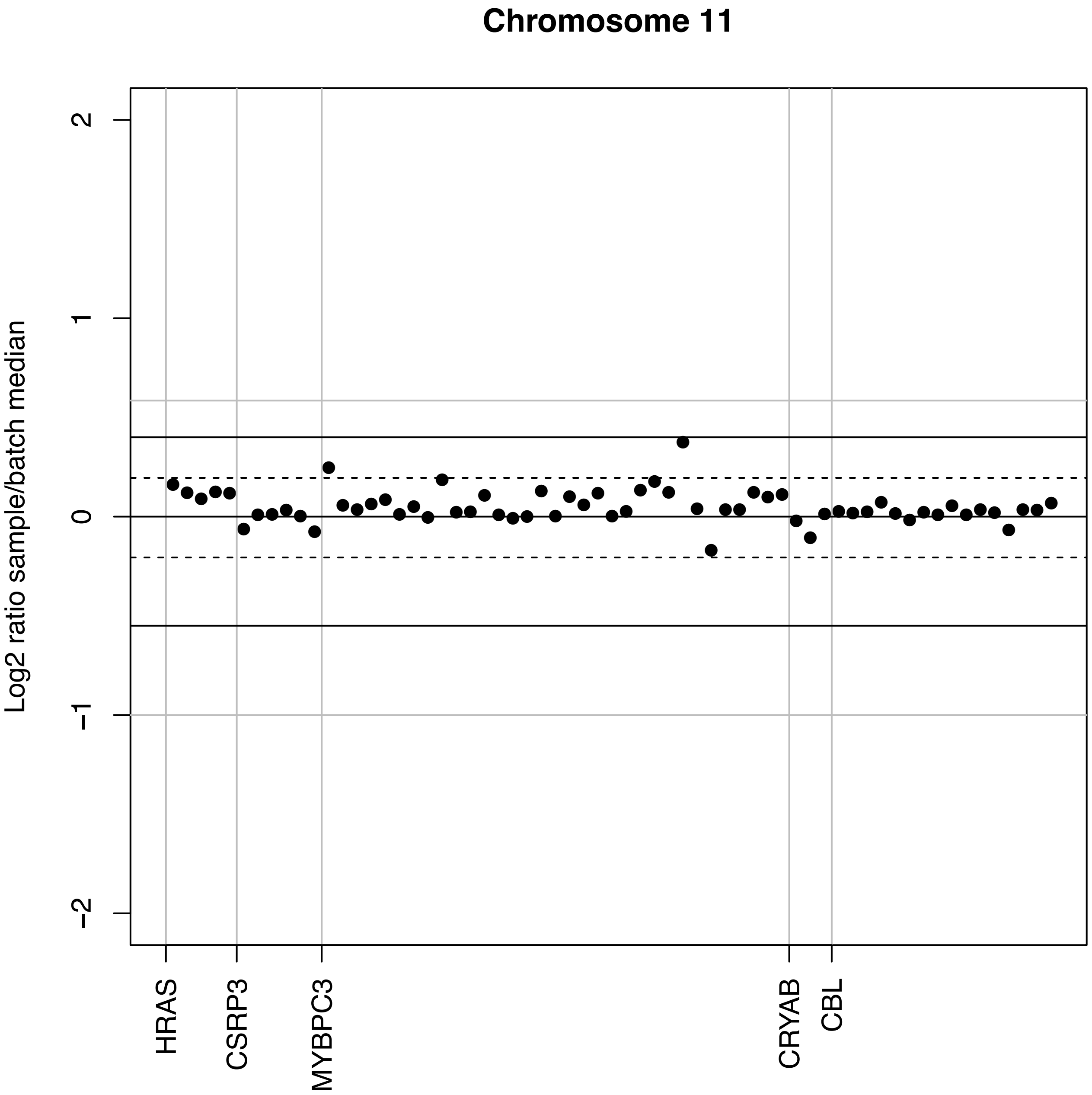

Chromosome 11
Log2 ratio sample/batch median
2
1
0
−1
−2
HRAS
CSRP3
MYBPC3
CRYAB
CBL

Case 19

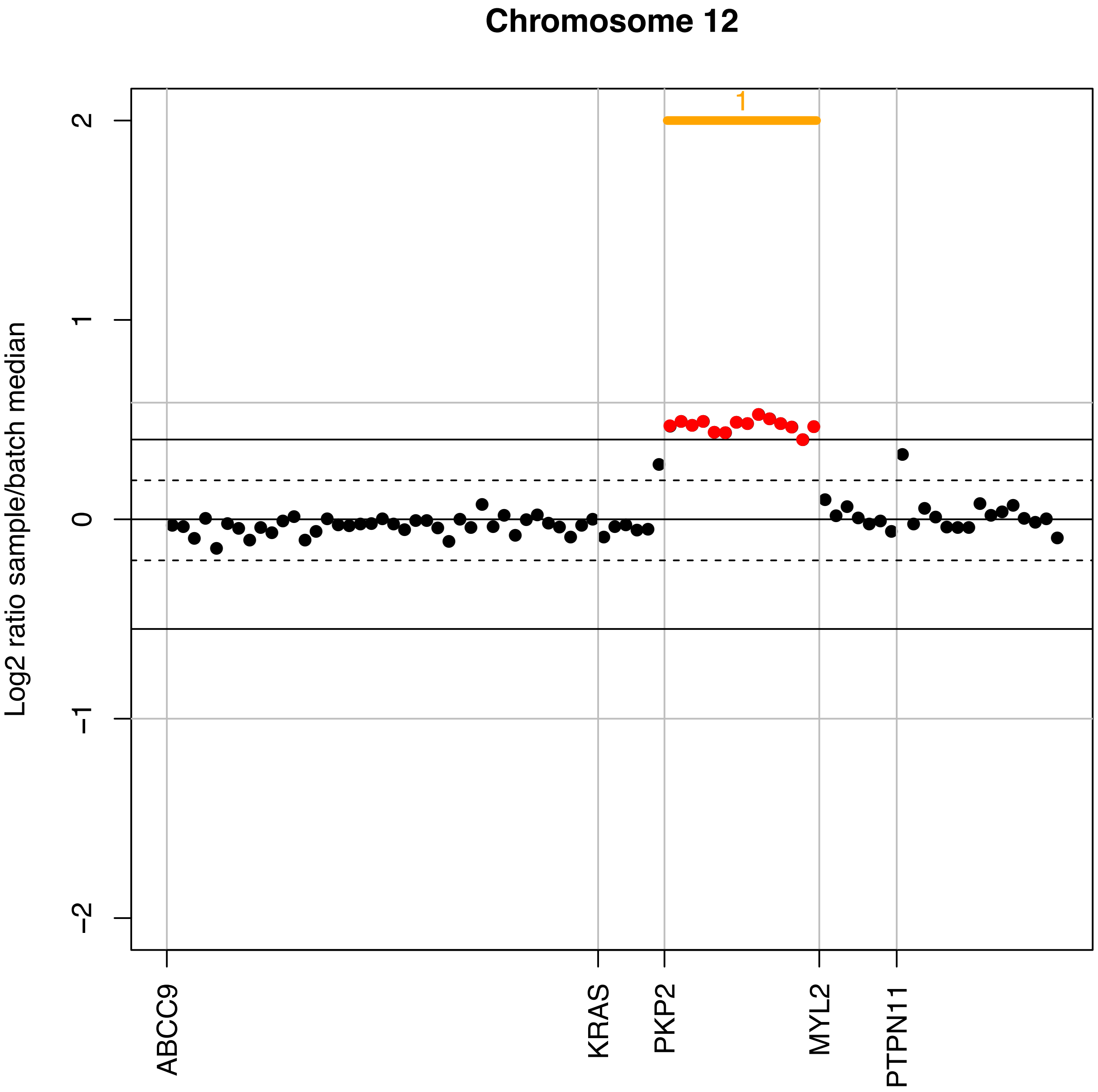
Chromosome 12
1
2
1
0
−1
−2
Log2 ratio sample/batch median
ABCC9
KRAS
PKP2
MYL2
PTPN11

Case 19

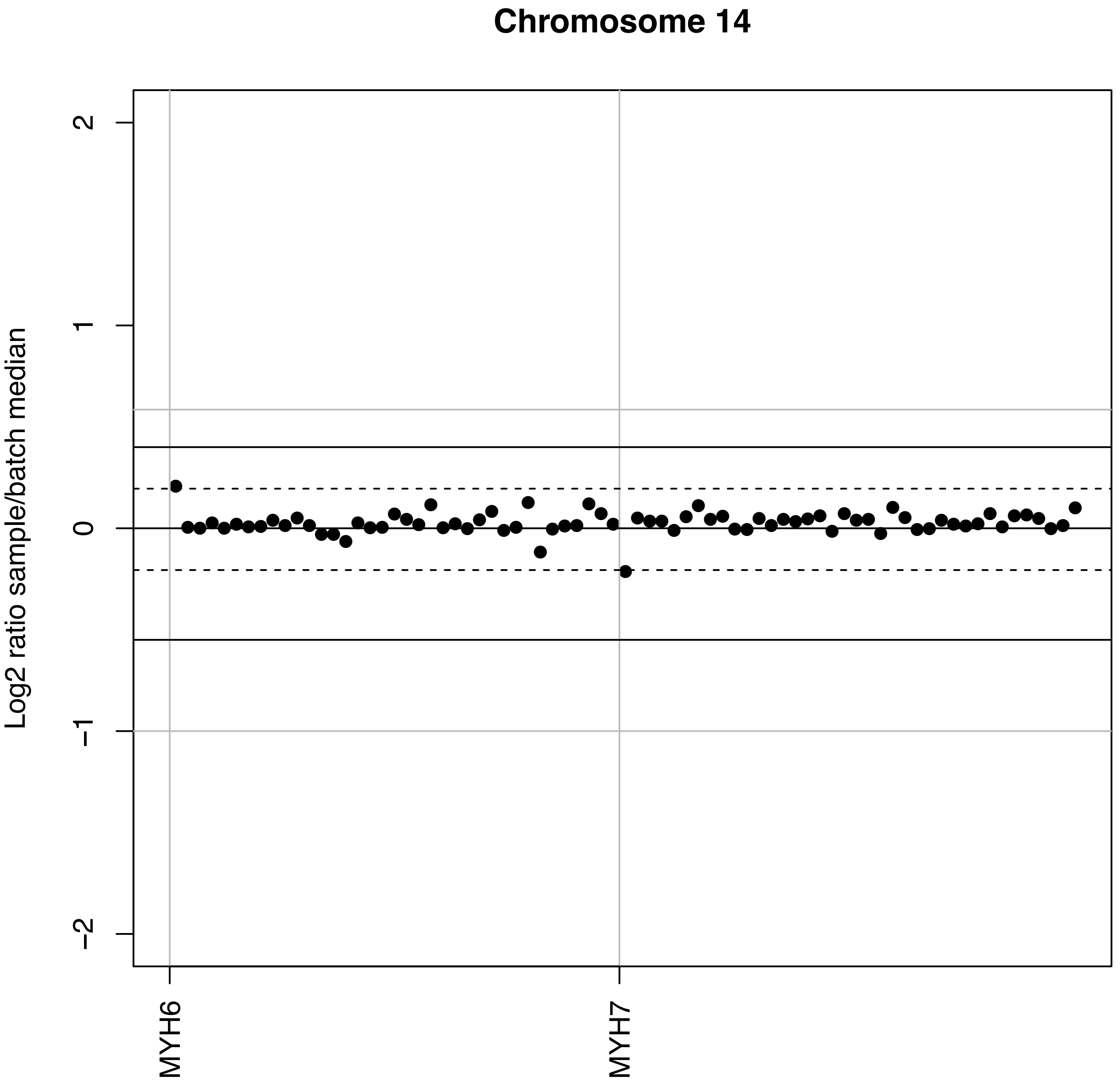

Chromosome 14
Log2 ratio sample/batch median
2
1
0
−1
−2
MYH6
MYH7

Case 19

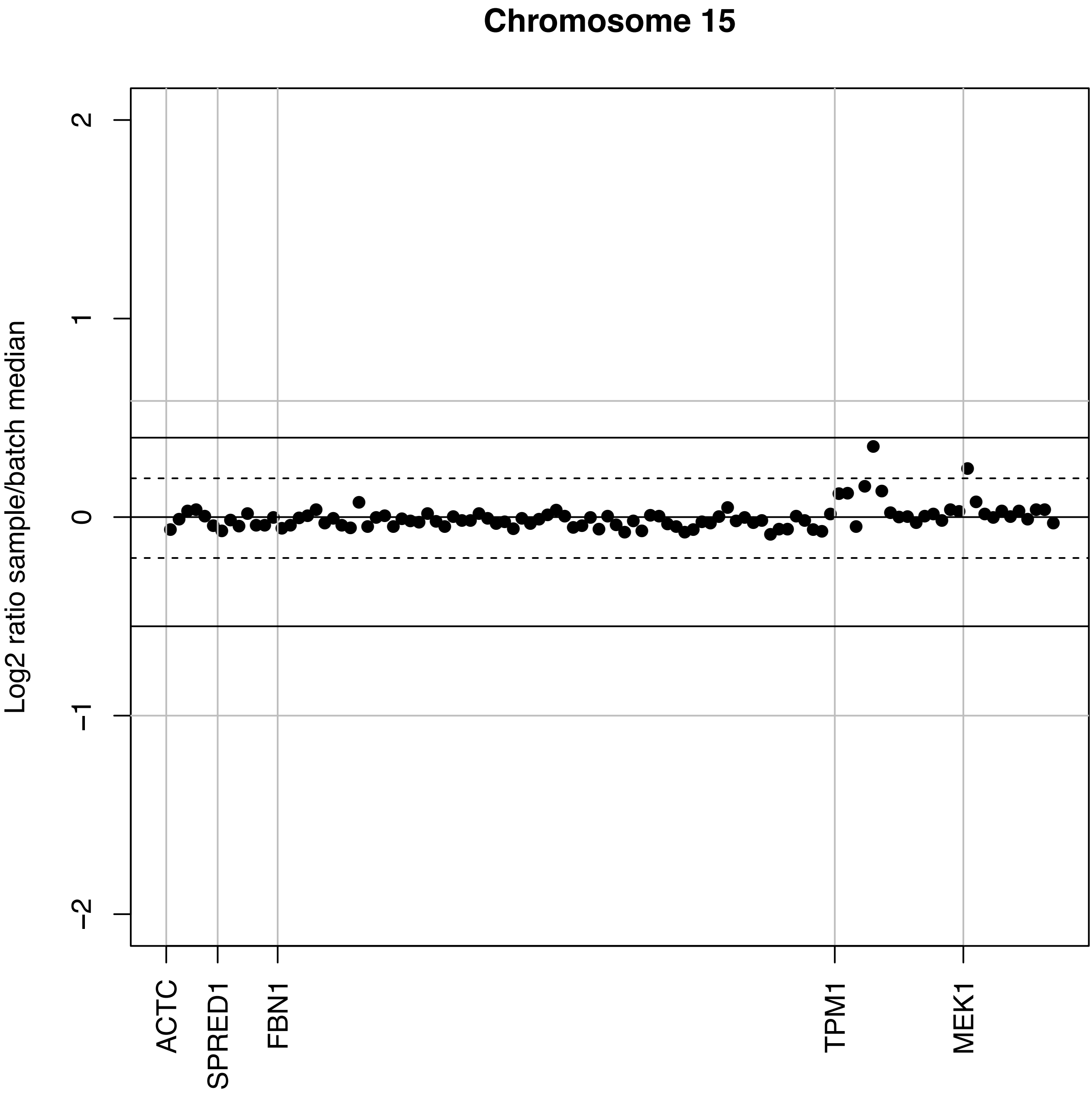
Chromosome 15
Log2 ratio sample/batch median
2
1
0
−1
−2
ACTC
SPRED1
FBN1
TPM1
MEK1

Case 19

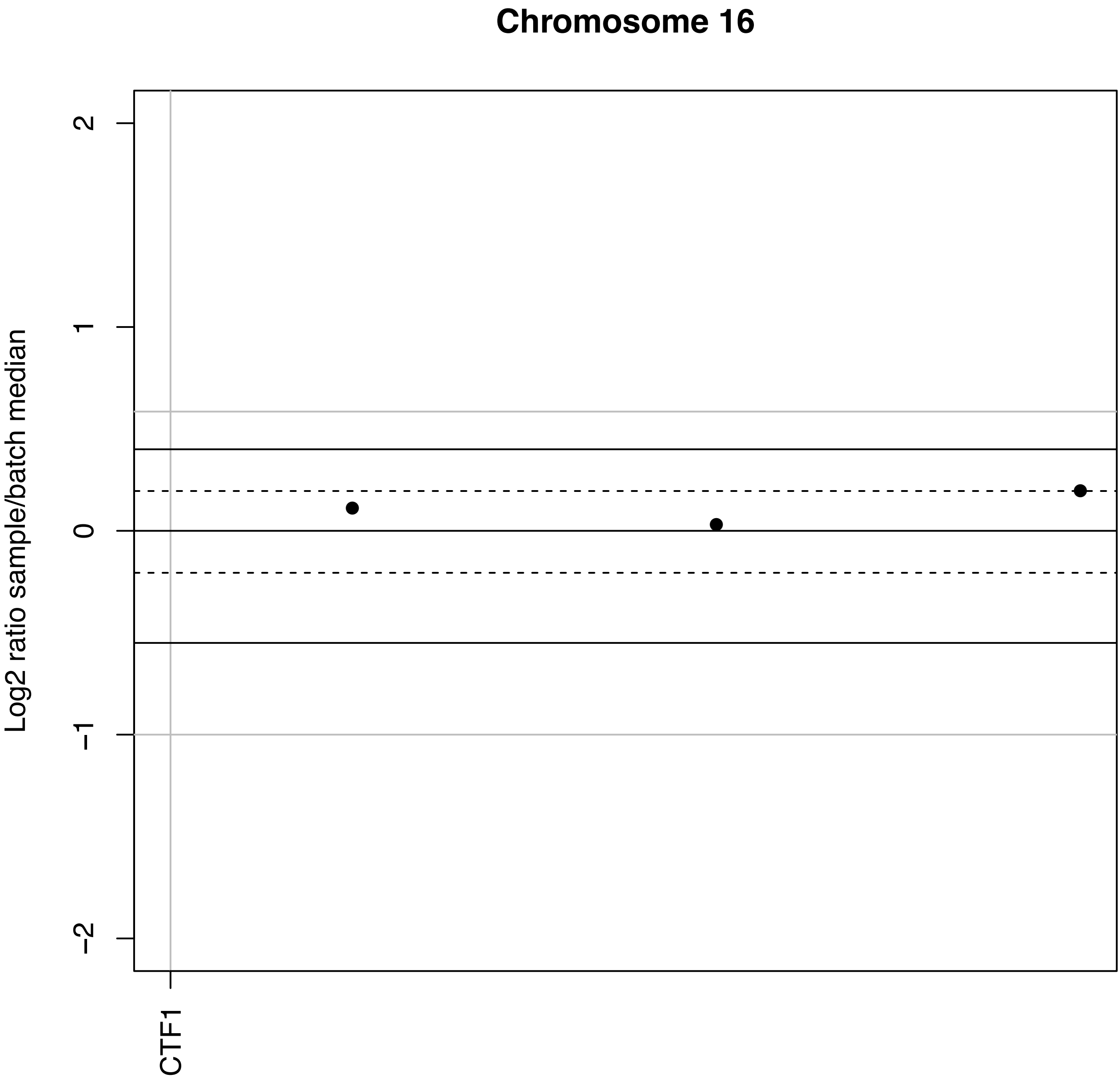

Case 19

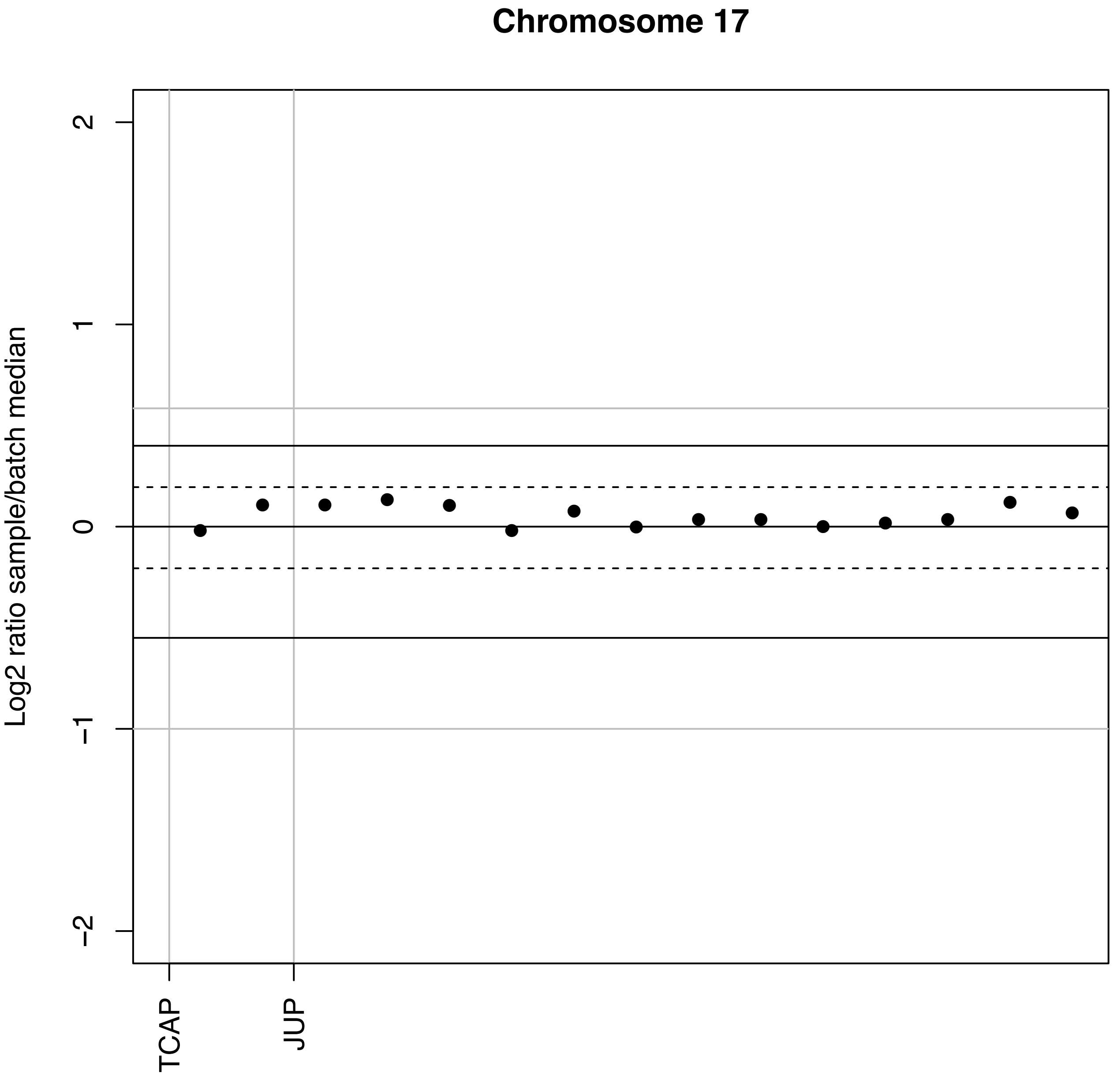

Chromosome 17
Log2 ratio sample/batch median
2
1
0
−1
−2
TCAP
JUP

Case 19

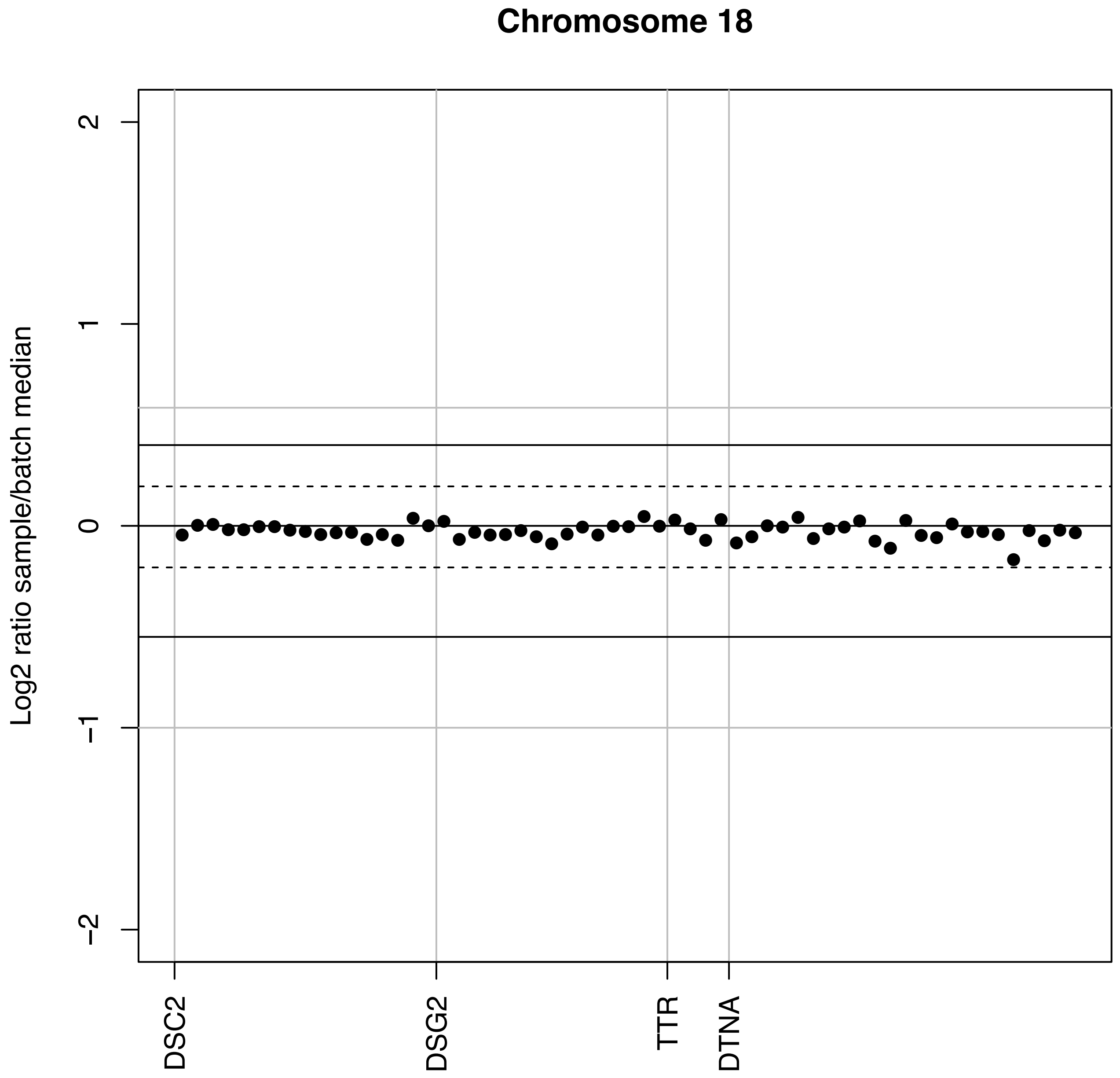
Chromosome 18
Log2 ratio sample/batch median
2
1
0
−1
−2
DSC2
DSG2
TTR
DTNA

Case 19

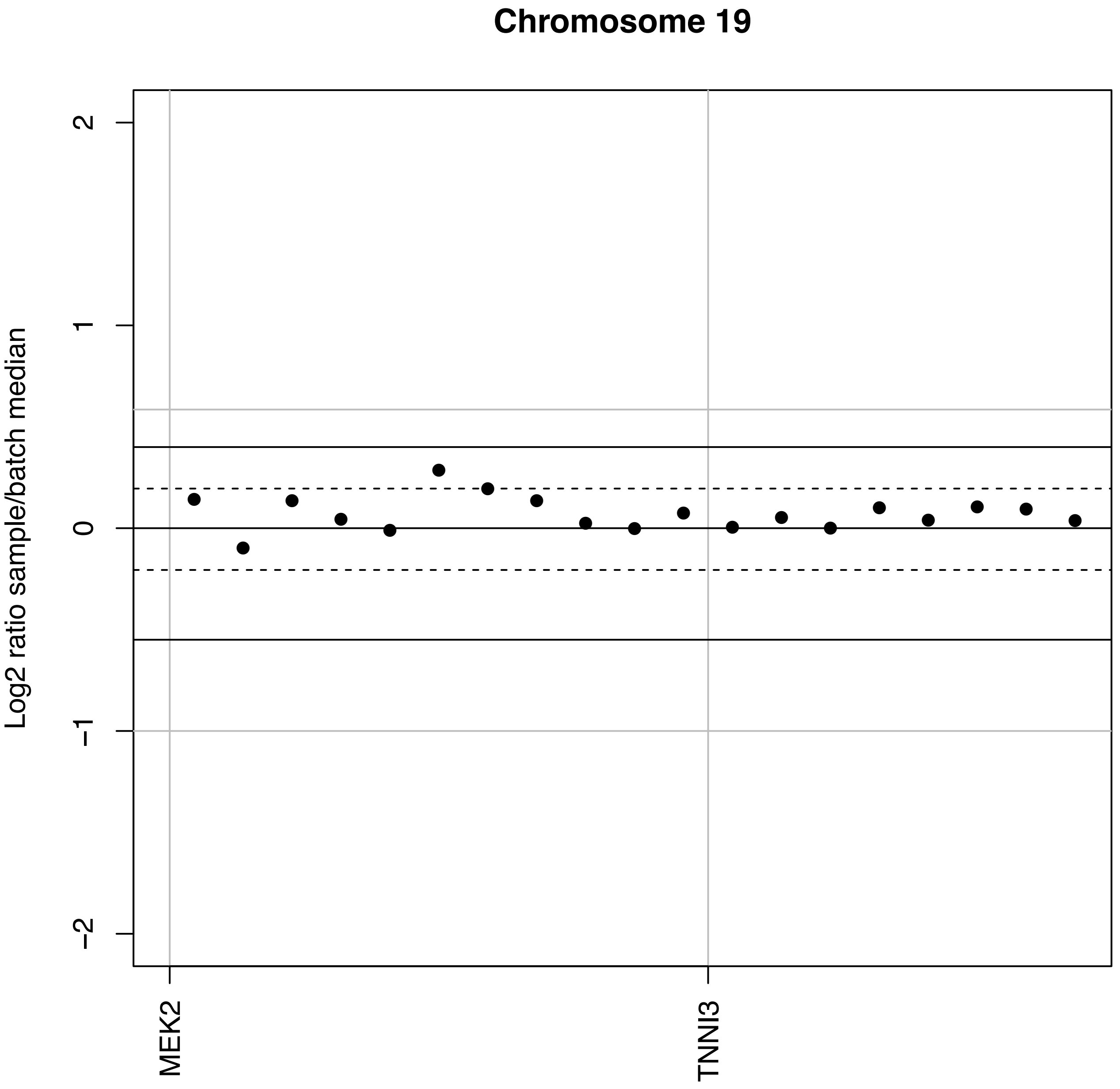
Chromosome 19
Log2 ratio sample/batch median
2
1
0
−1
−2
MEK2
TNNI3

Case 19

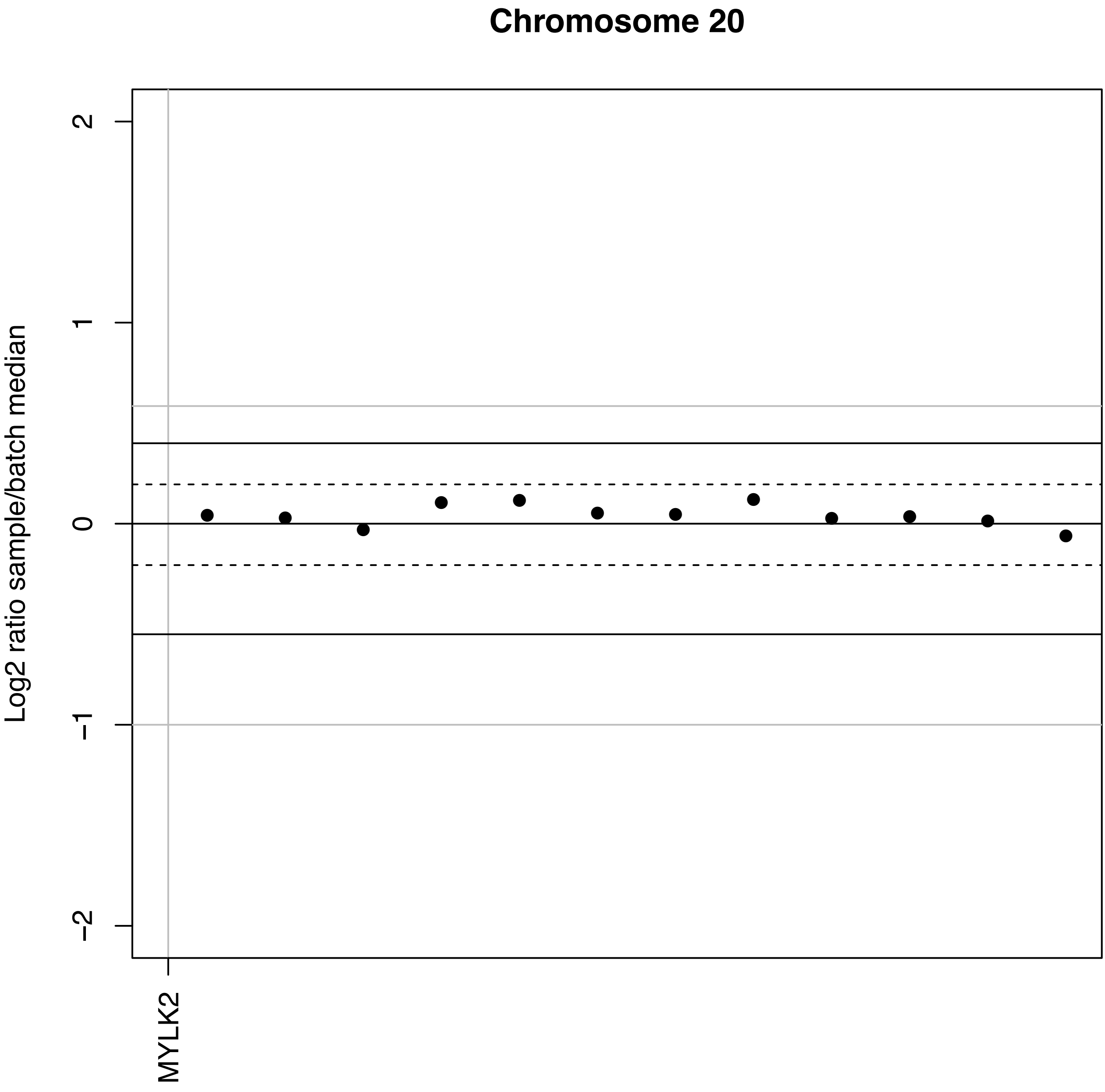

Case 19

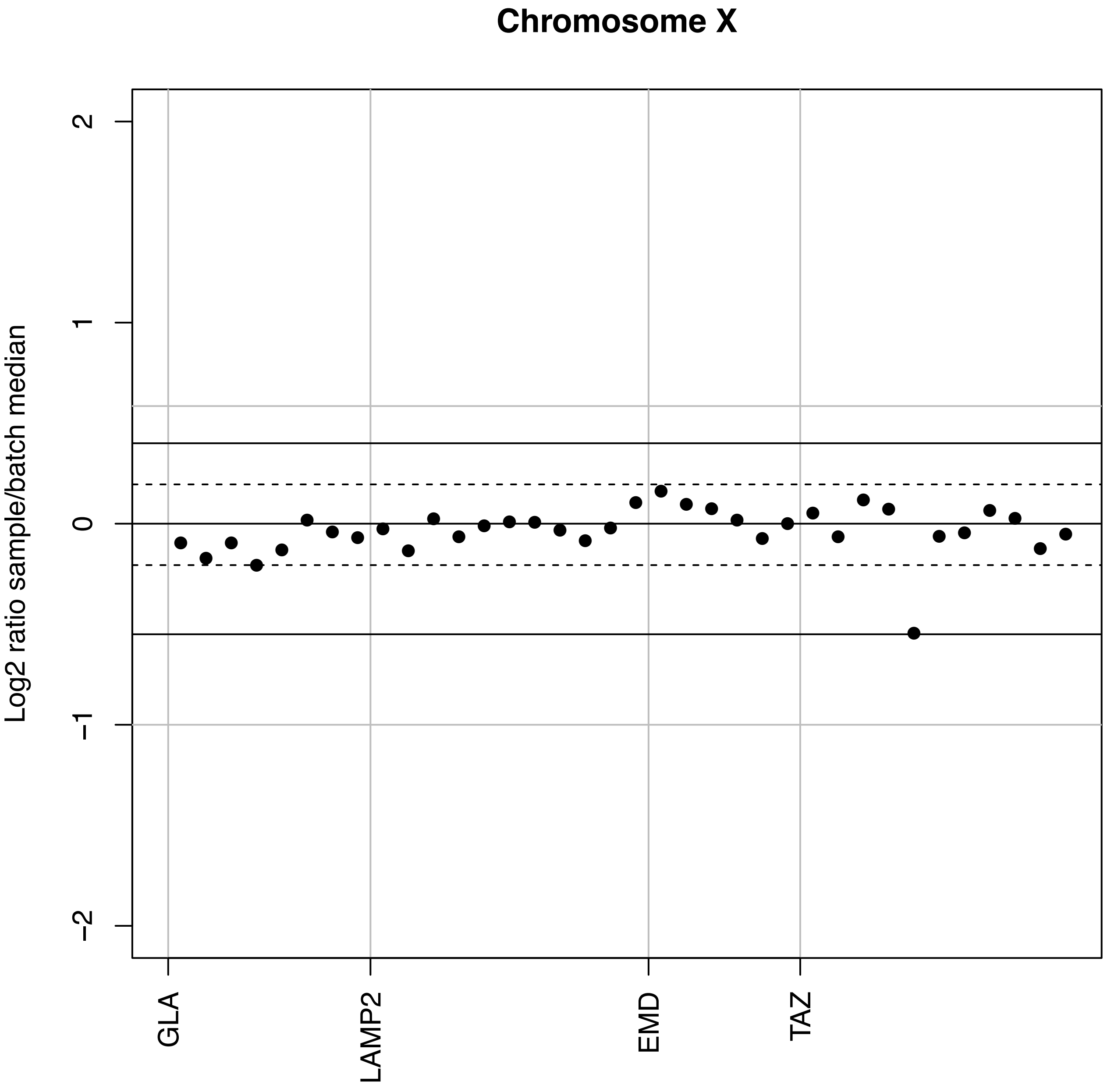
Chromosome X
Log2 ratio sample/batch median
2
1
0
−1
−2
GLA
LAMP2
EMD
TAZ
