# Supplementary material for: VisCap: inference and visualization of germ-line copy-number variants from targeted clinical sequencing data
Source: Genet Med. 2015 Dec 17;18(7):712–9. doi: 10.1038/gim.2015.156 (PMC4940431; doi:10.1038/gim.2015.156)
Supplement: Supplementary Information [file gim2015156x1.zip › Supplementary_File_3.zip!/Supplementary_File_2/Case_23.pdf]

Case 23

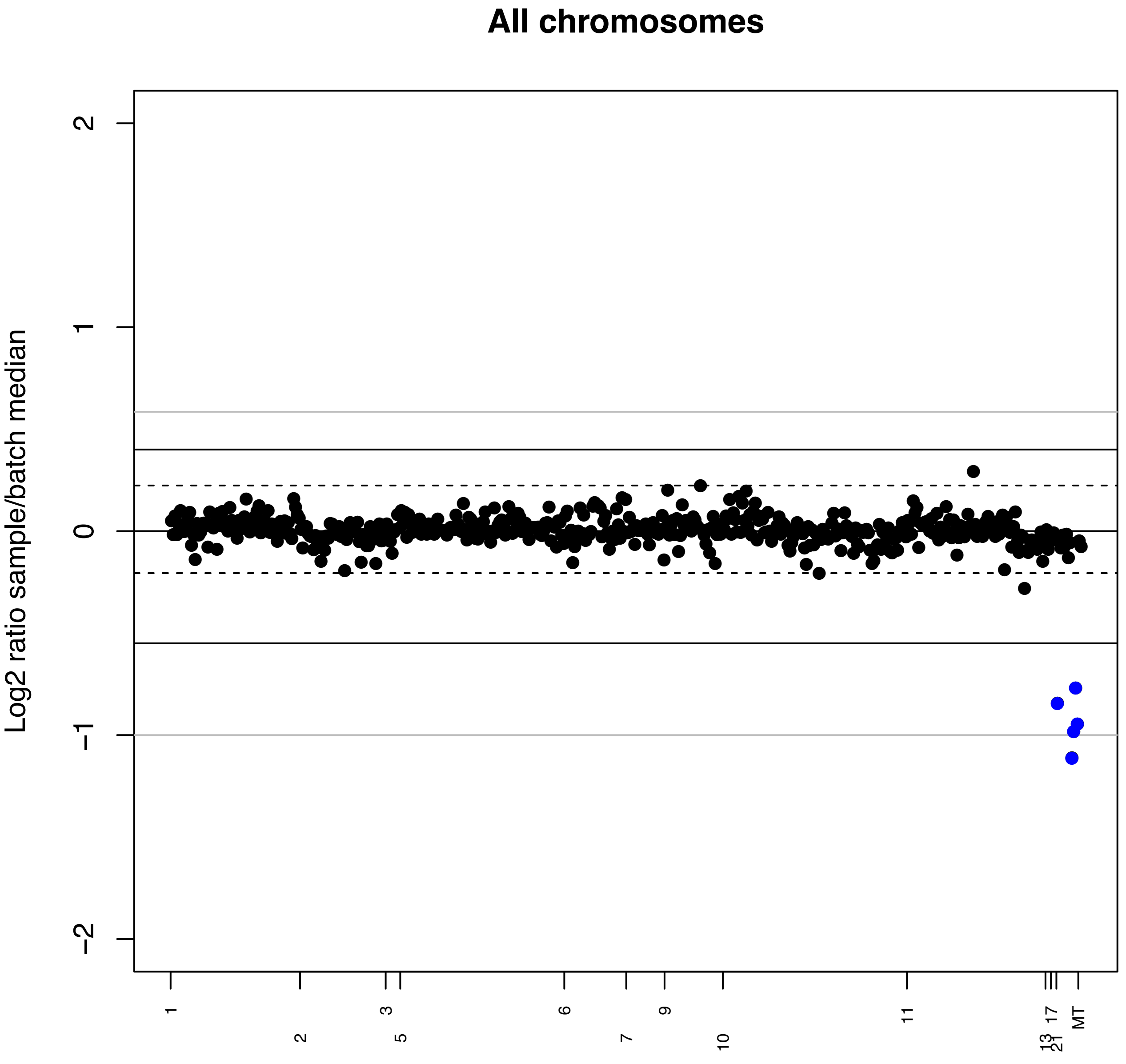
All chromosomes
Log2 ratio sample/batch median
2
1
0
−1
−2
1
2
3
5
6
7
9
10
11
13
17
21
MT

Case 23

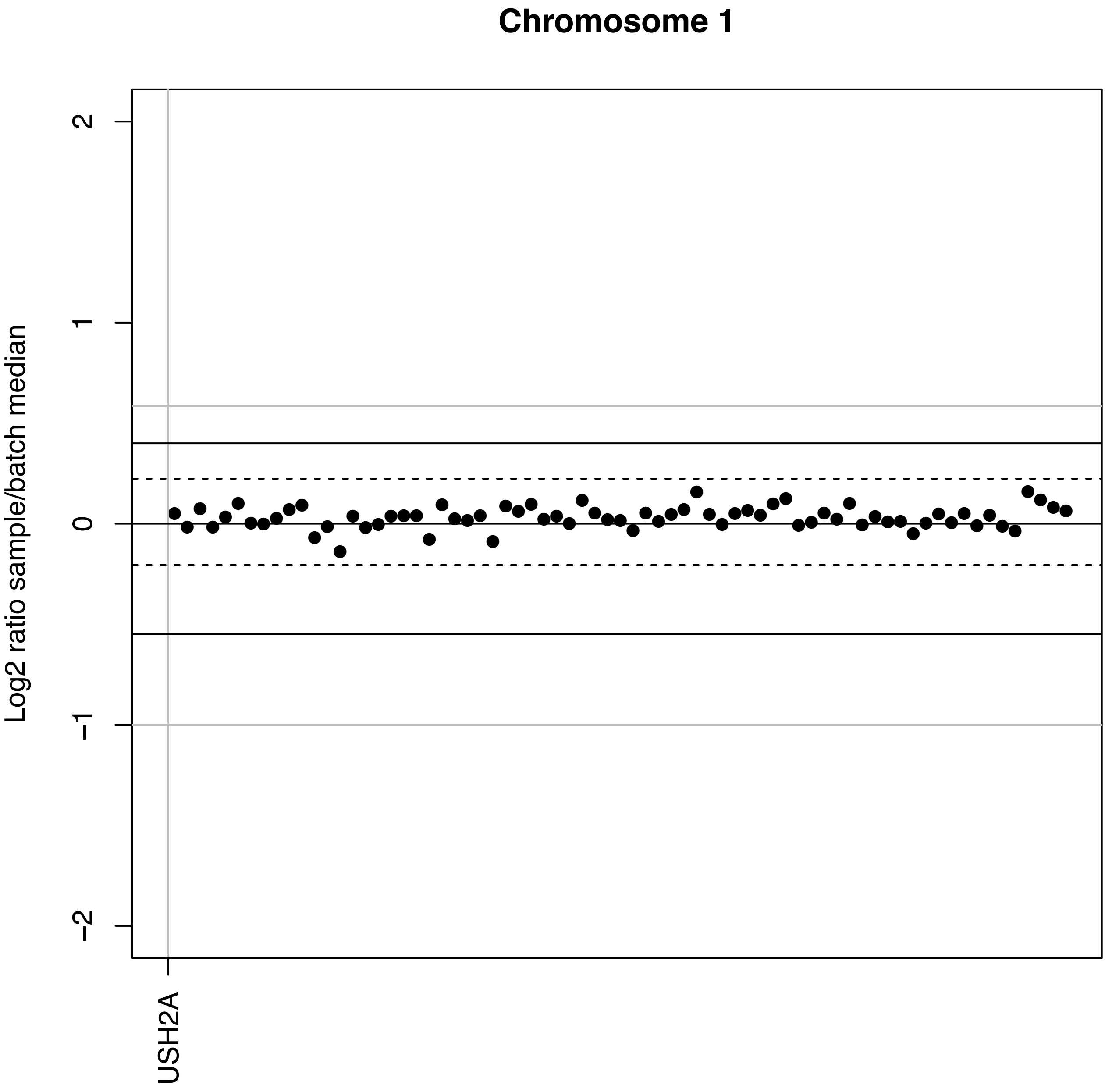

Case 23

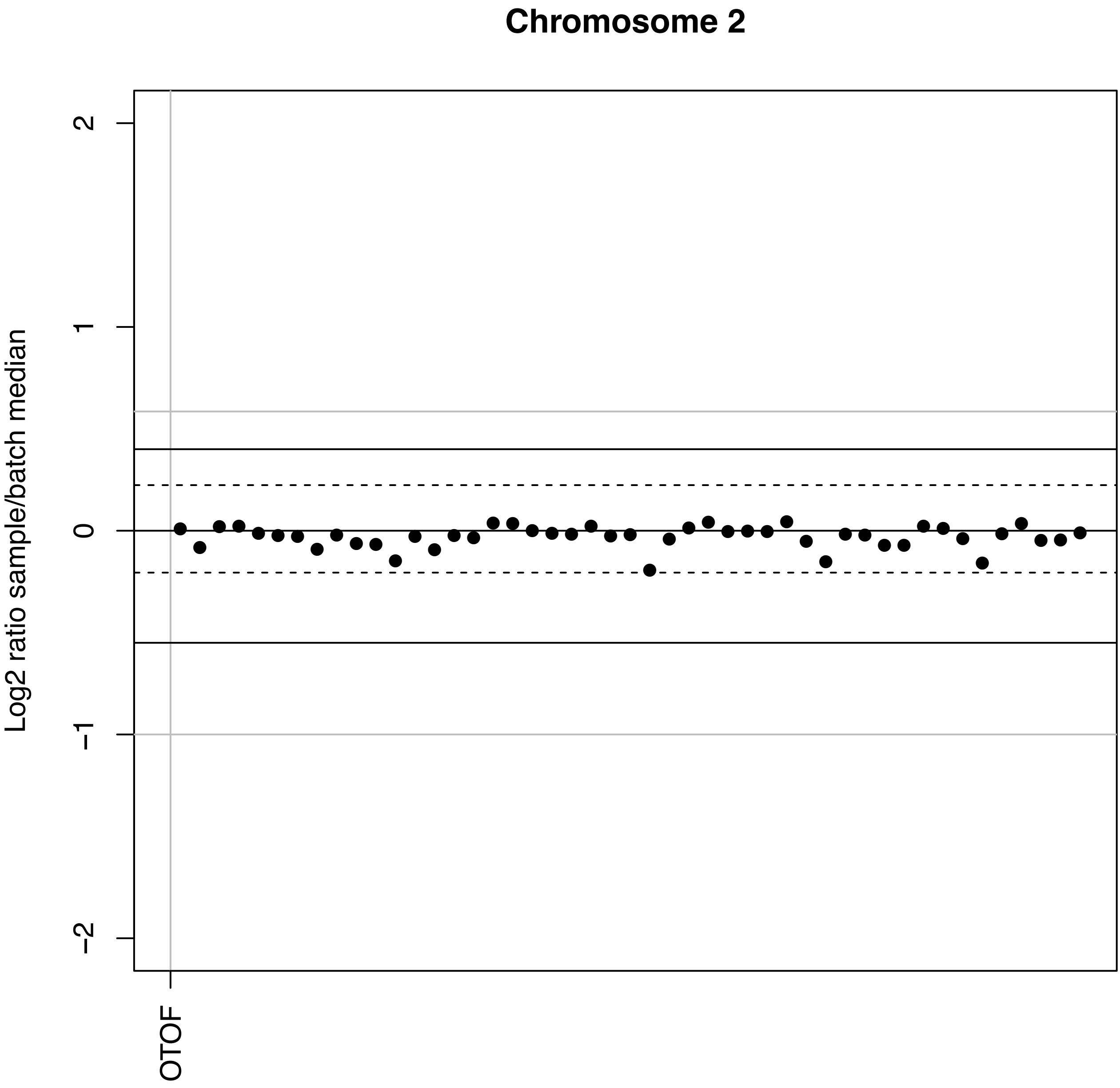

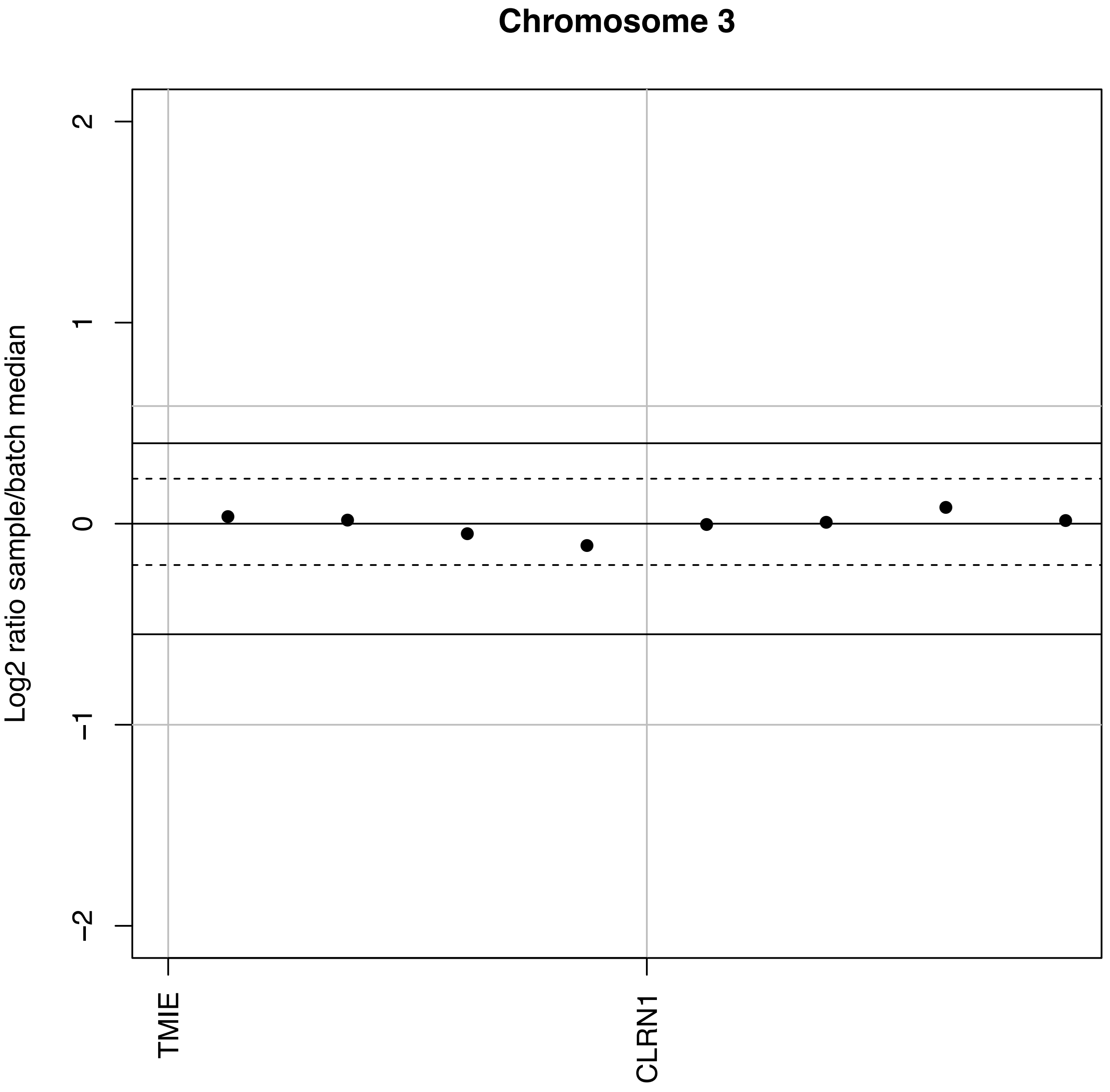
Chromosome 3
Log2 ratio sample/batch median
2
1
0
−1
−2
TMIE
CLRN1

Case 23

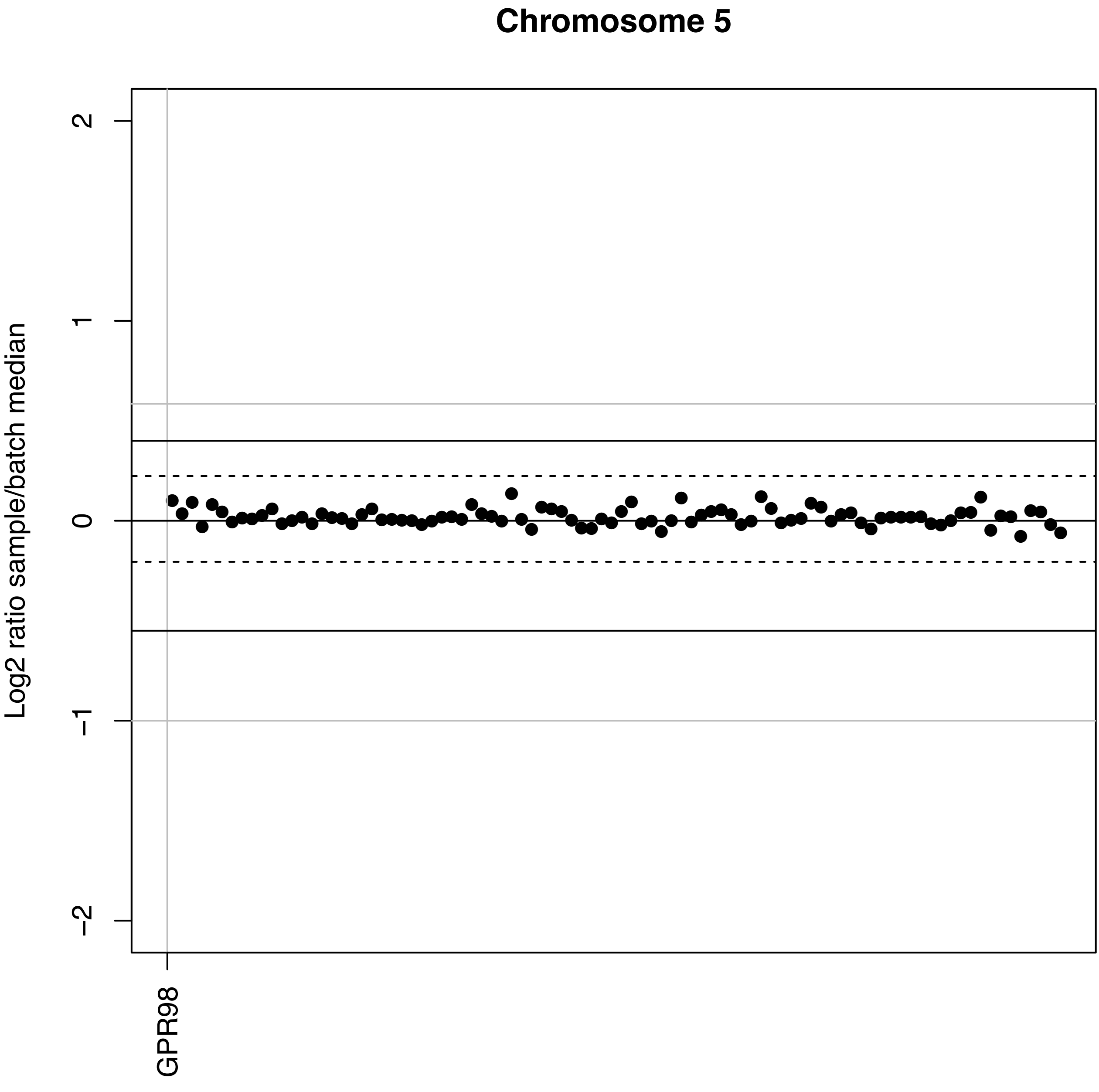

Case 23

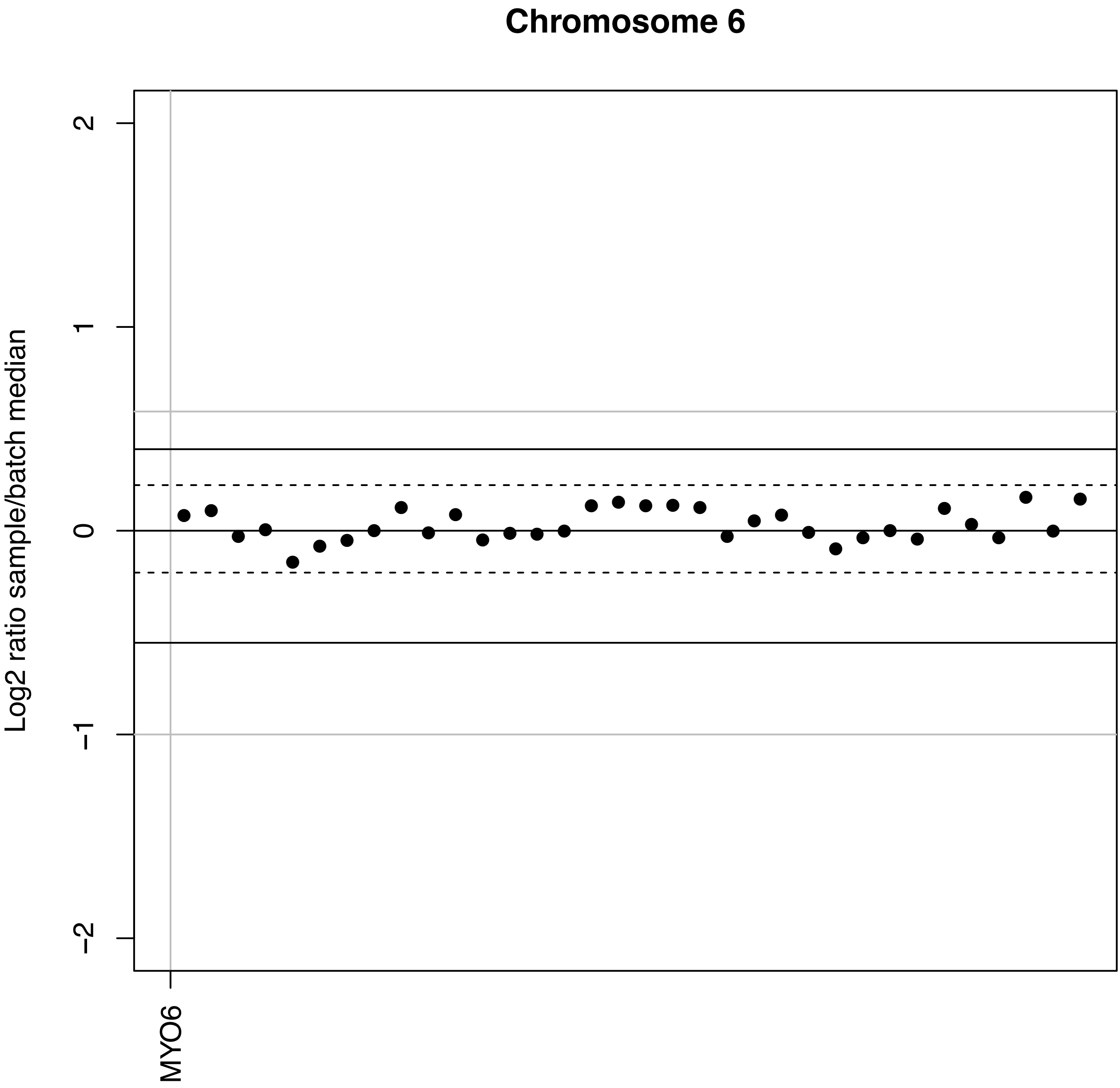

Case 23

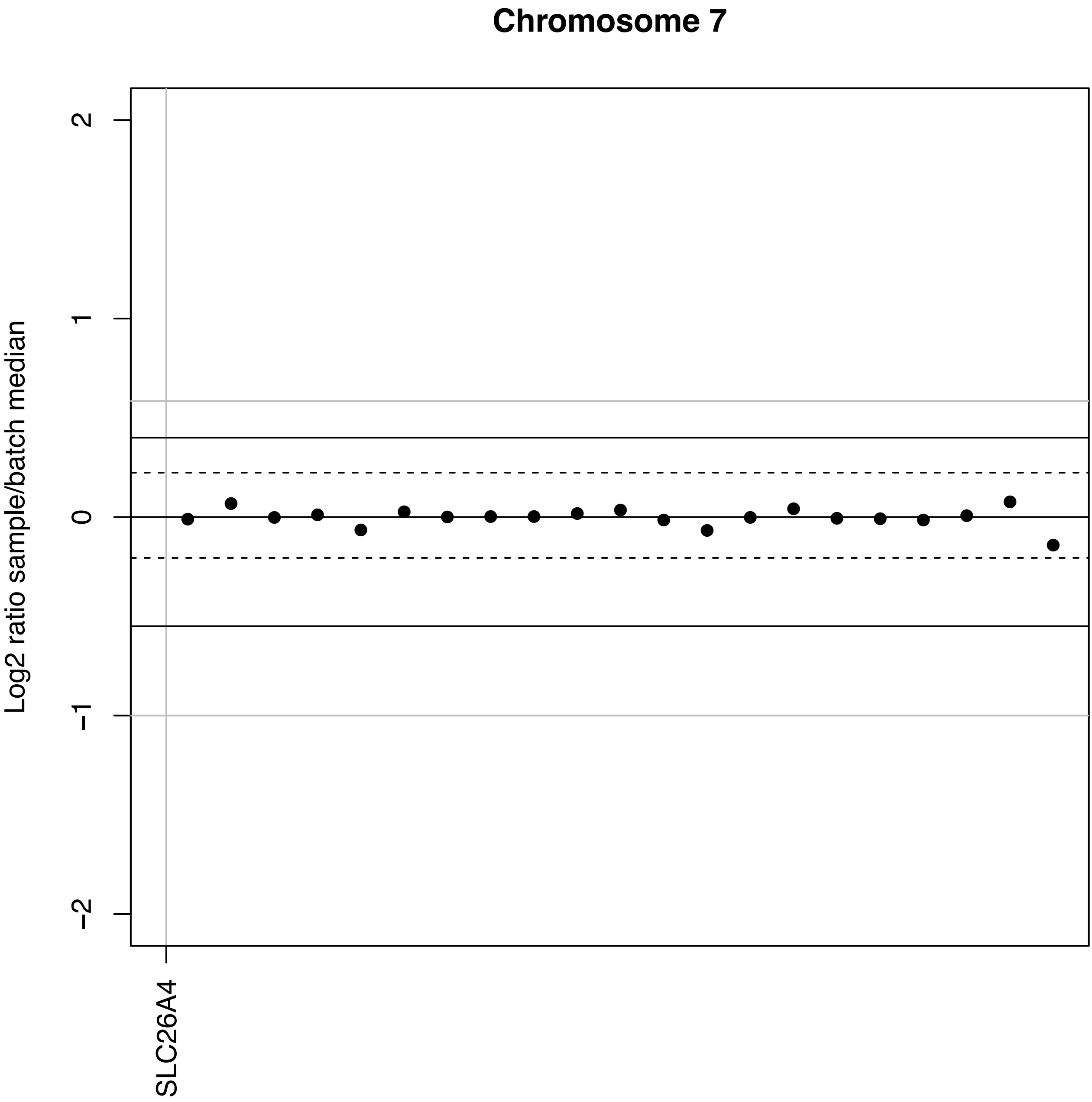

Case 23

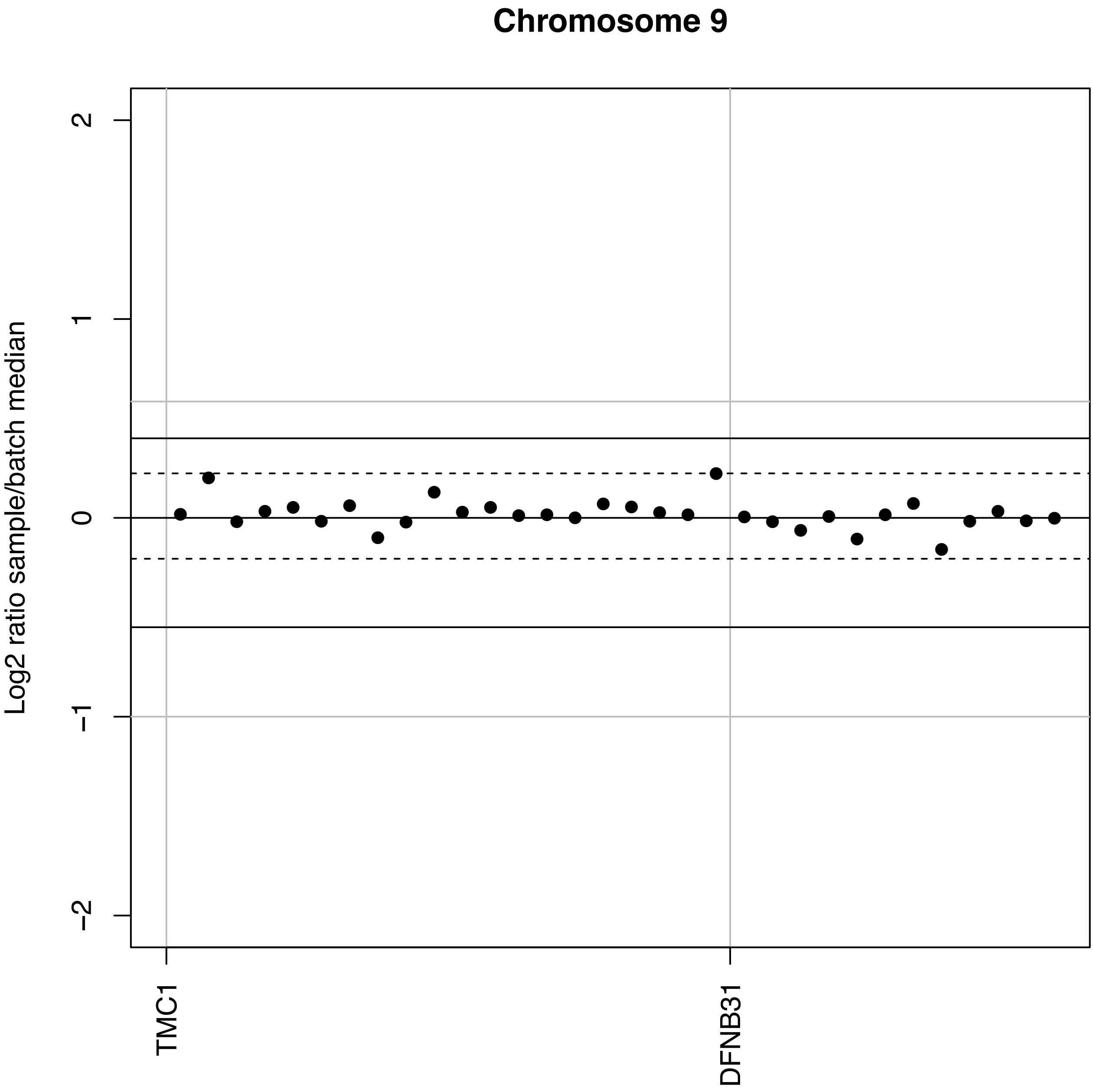

Chromosome 9
Log2 ratio sample/batch median
2
1
0
−1
−2
TMC1
DFNB31

Case 23

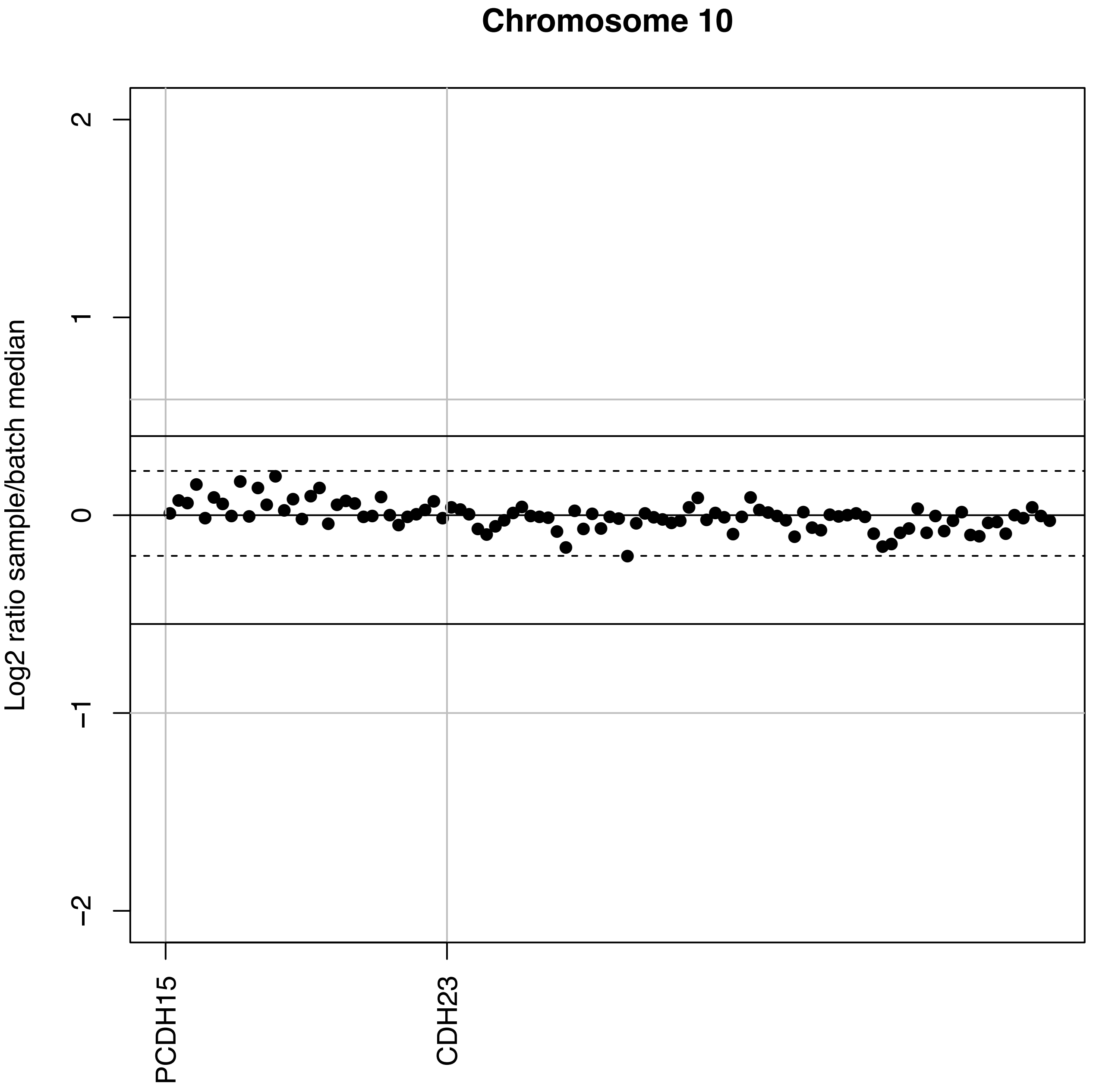

Chromosome 10
Log2 ratio sample/batch median
2
1
0
−1
−2
PCDH15
CDH23

Case 23

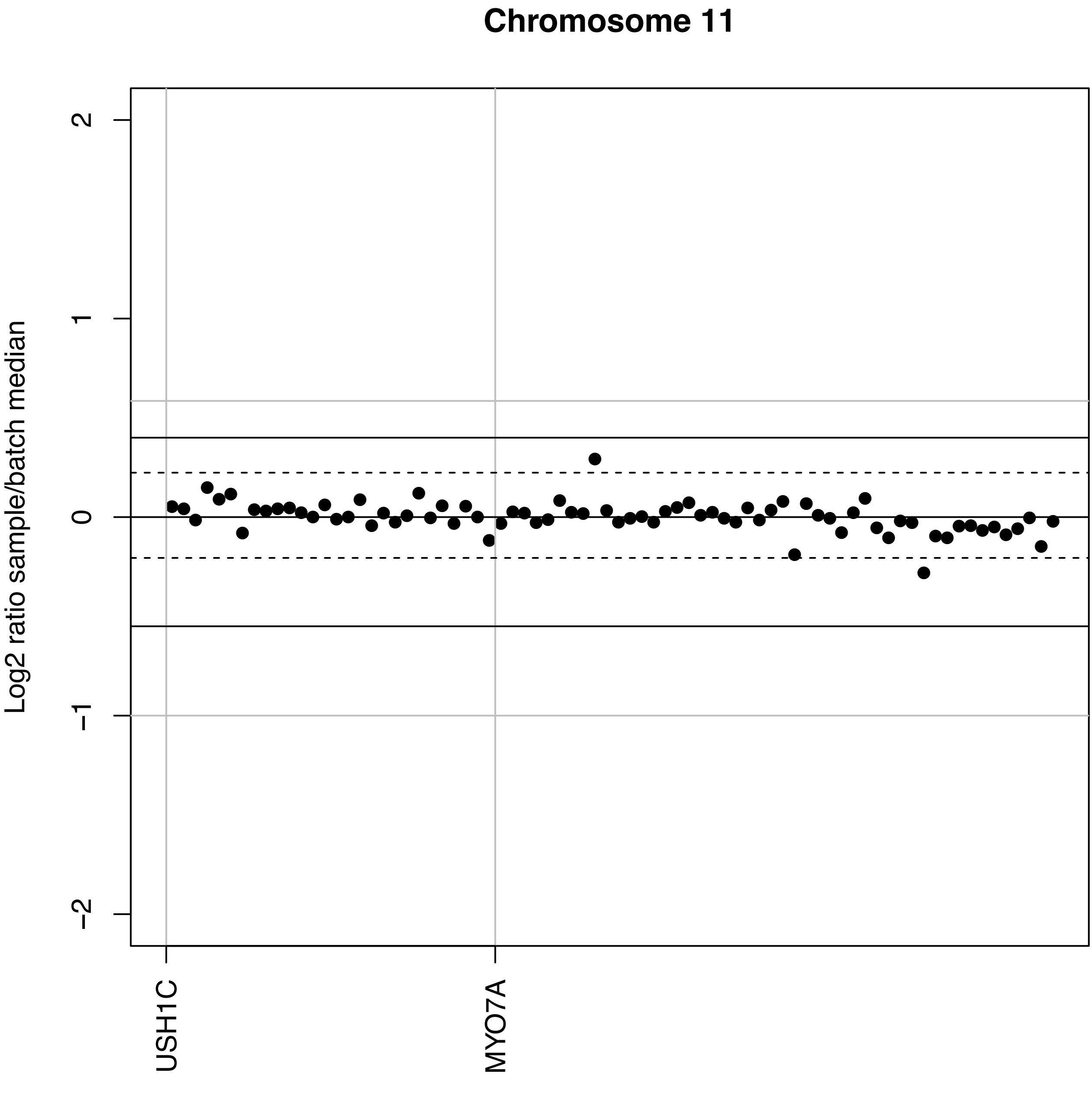

Chromosome 11
Log2 ratio sample/batch median
2
1
0
−1
−2
USH1C
MYO7A

Case 23

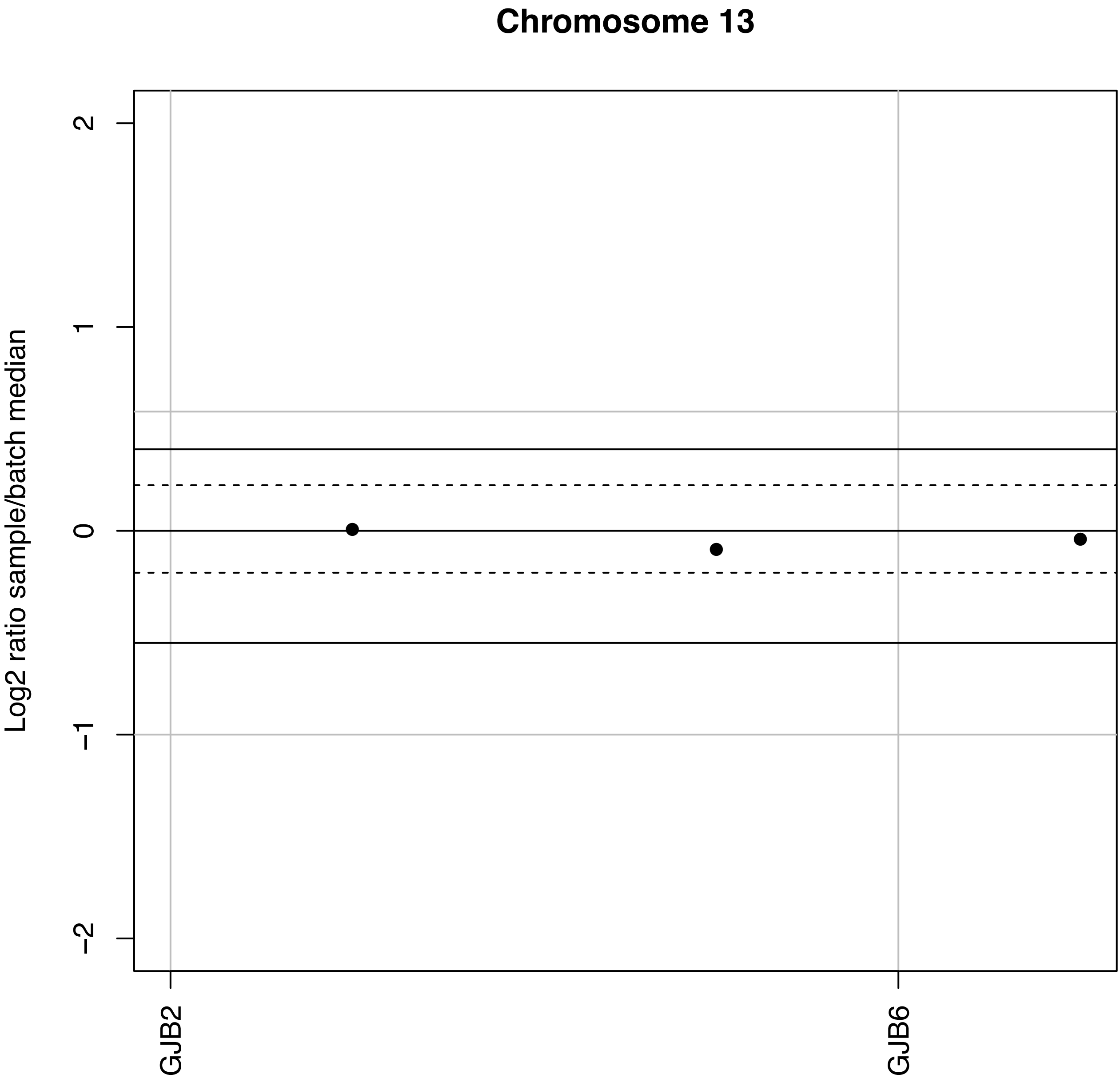
Chromosome 13
Log2 ratio sample/batch median
2
1
0
−1
−2
GJB2
GJB6

Case 23

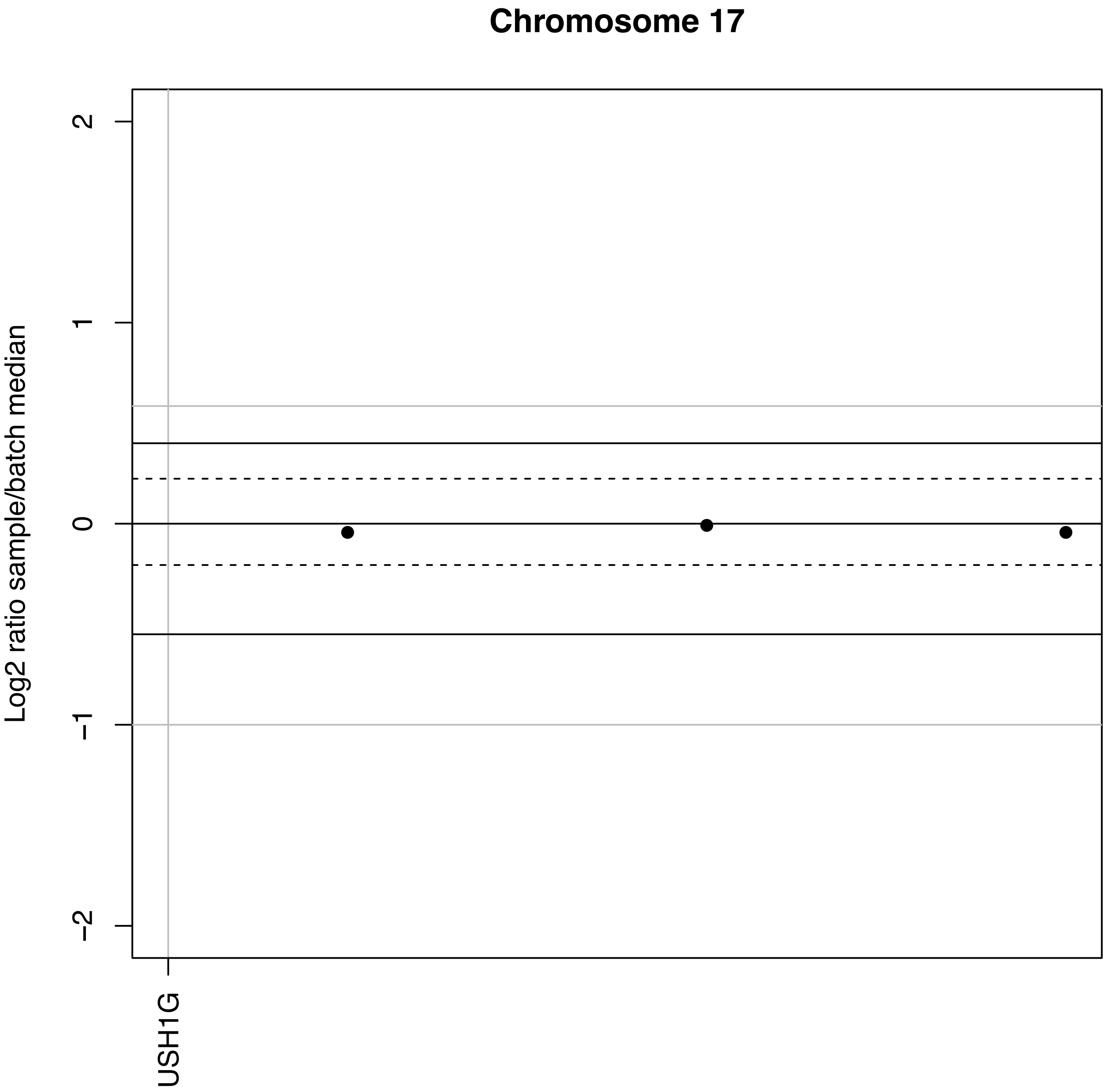

Case 23

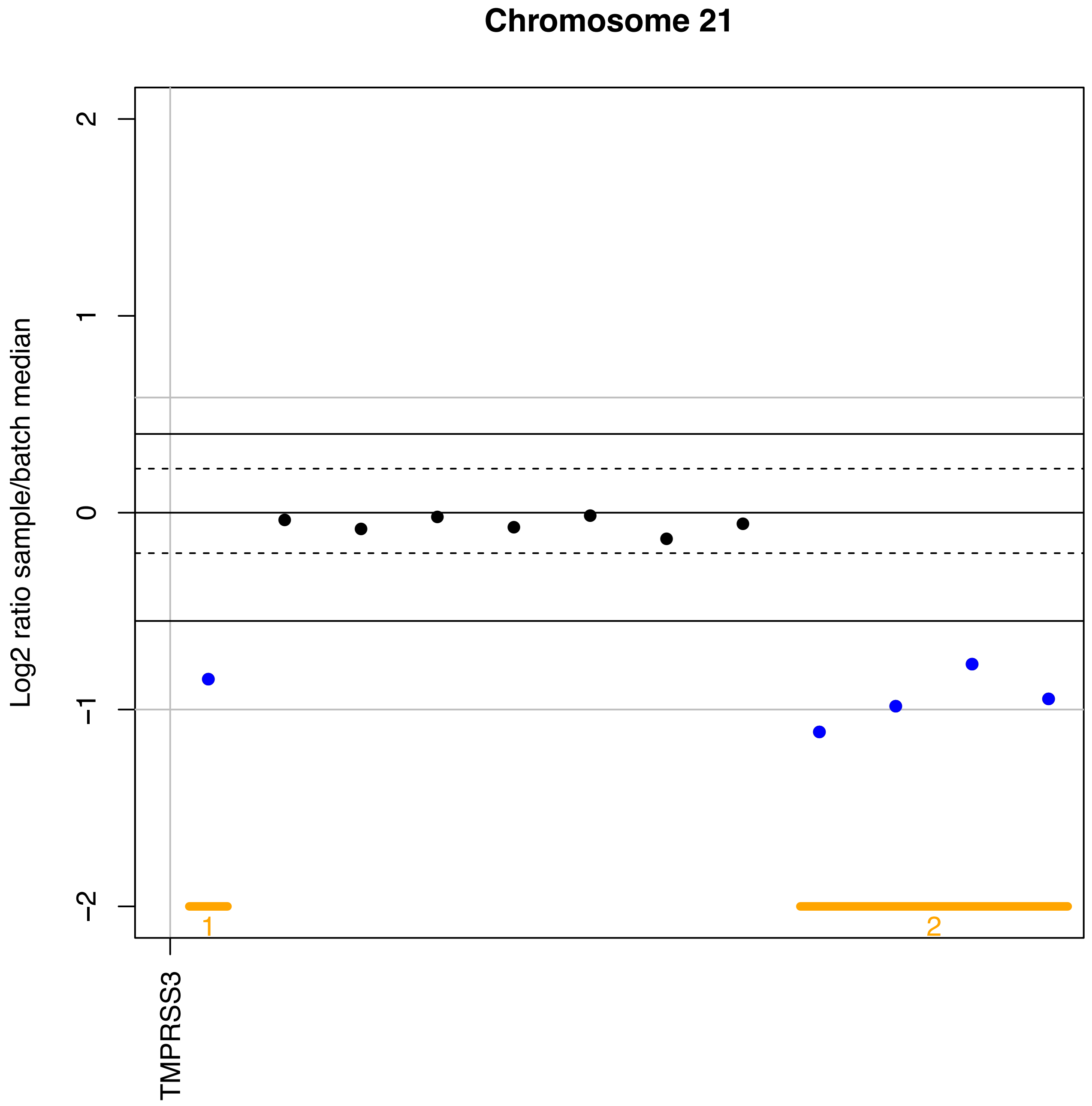

Chromosome 21
Log2 ratio sample/batch median
2
1
0
−1
−2
1
2
TMPRSS3
