# Supplementary material for: VisCap: inference and visualization of germ-line copy-number variants from targeted clinical sequencing data
Source: Genet Med. 2015 Dec 17;18(7):712–9. doi: 10.1038/gim.2015.156 (PMC4940431; doi:10.1038/gim.2015.156)
Supplement: Supplementary Information [file gim2015156x1.zip › Supplementary_File_3.zip!/Supplementary_File_2/Case_26.pdf]

Case 26

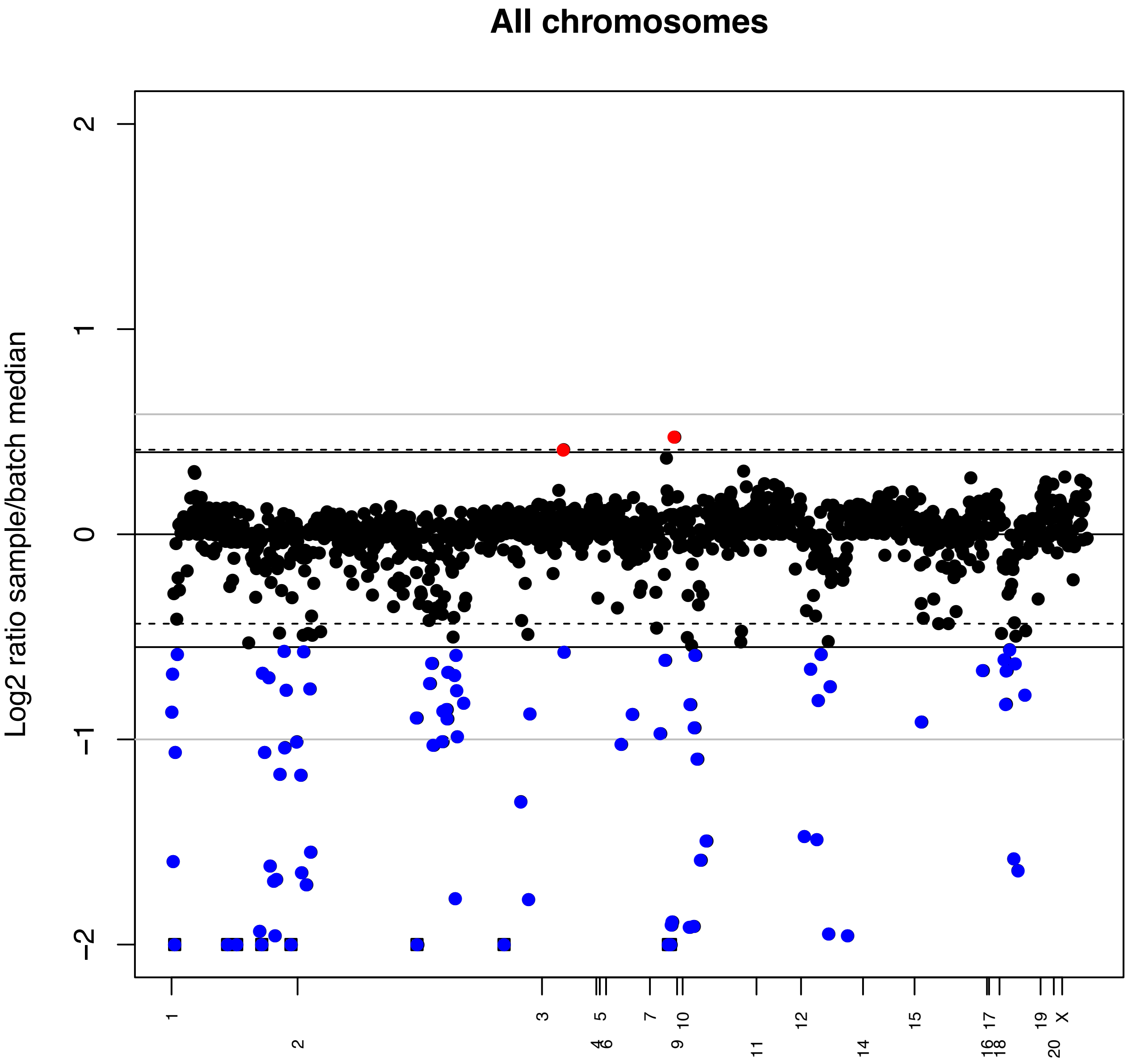

All chromosomes
Log2 ratio sample/batch median
2
1
0
−1
−2
1
2
3
4
5
6
7
9
10
11
12
14
15
16
17
18
19
20
X

Case 26

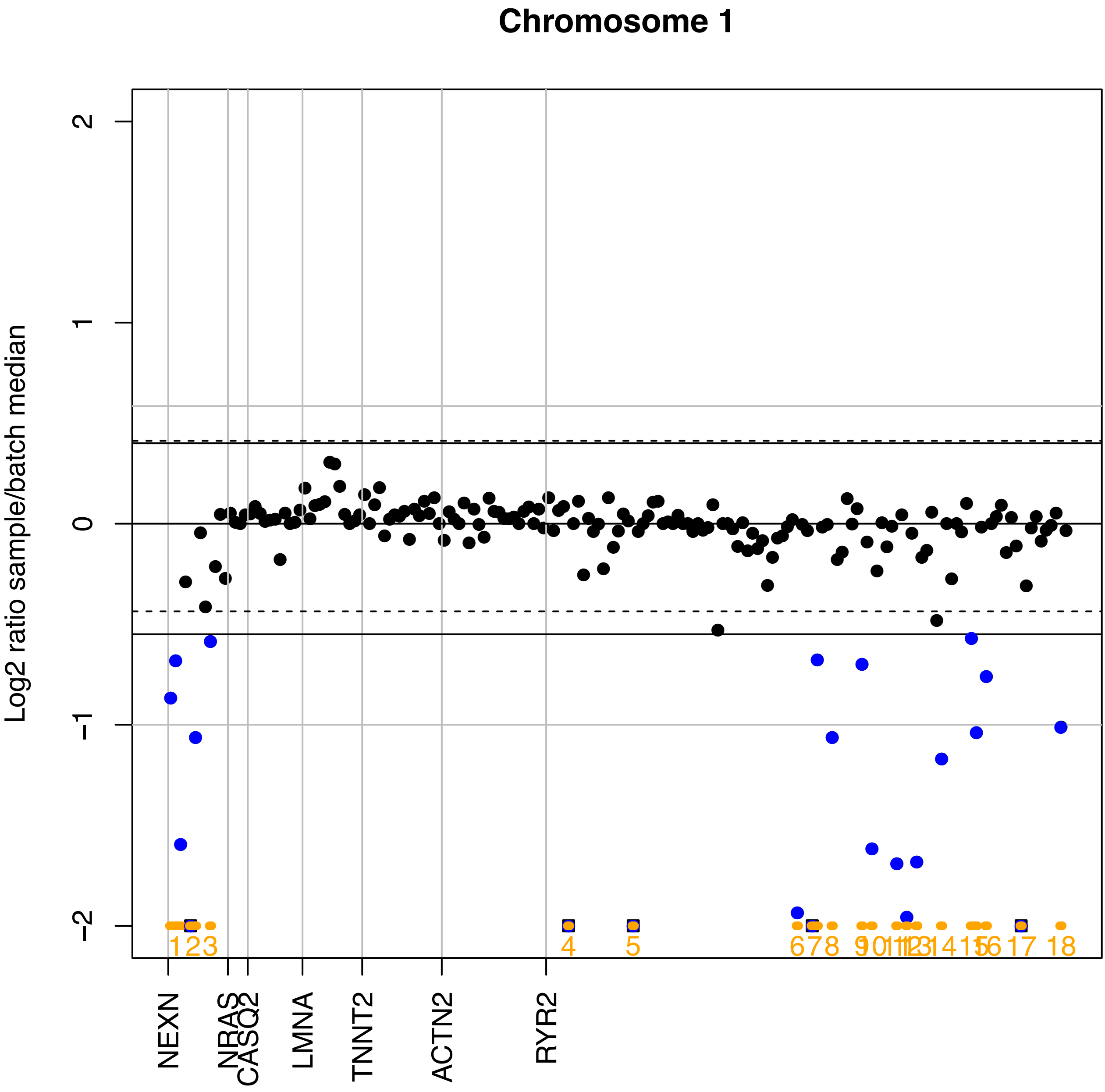
Chromosome 1
Log2 ratio sample/batch median
2
1
0
−1
−2
1 2 3
4
5
6 7 8
9 10 11 12 13 14 15 16 17 18
NEXN
NRAS
CASQ2
LMNA
TNNT2
ACTN2
RYR2

Case 26

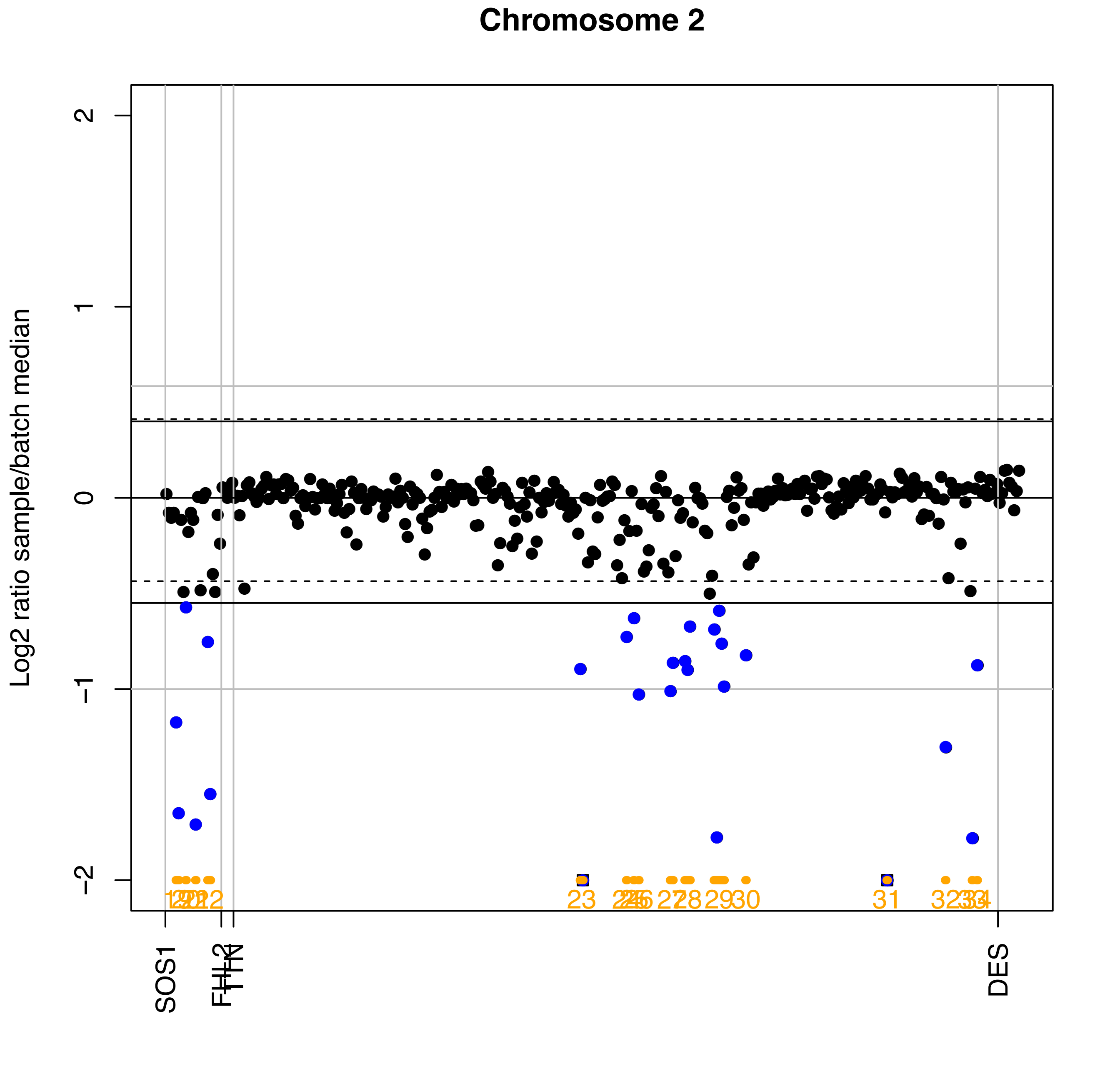

Chromosome 2
Log2 ratio sample/batch median
2
1
0
−1
−2
SOS1
FHL2
TTN
DES
23
30
31

Case 26

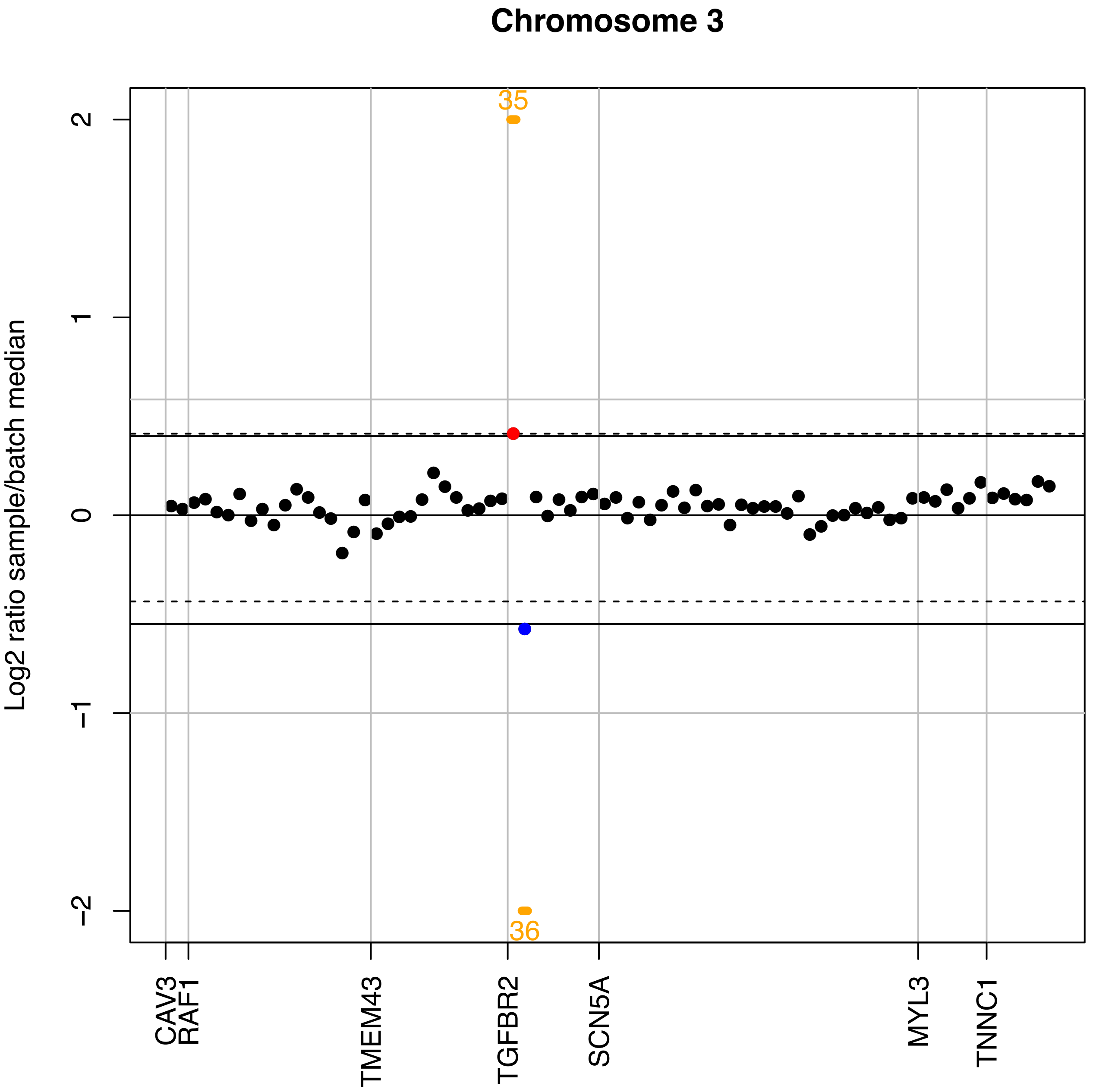

Chromosome 3
Log2 ratio sample/batch median
2
1
0
−1
−2
35
36
CAV3
RAF1
TMEM43
TGFBR2
SCN5A
MYL3
TNNC1

Case 26

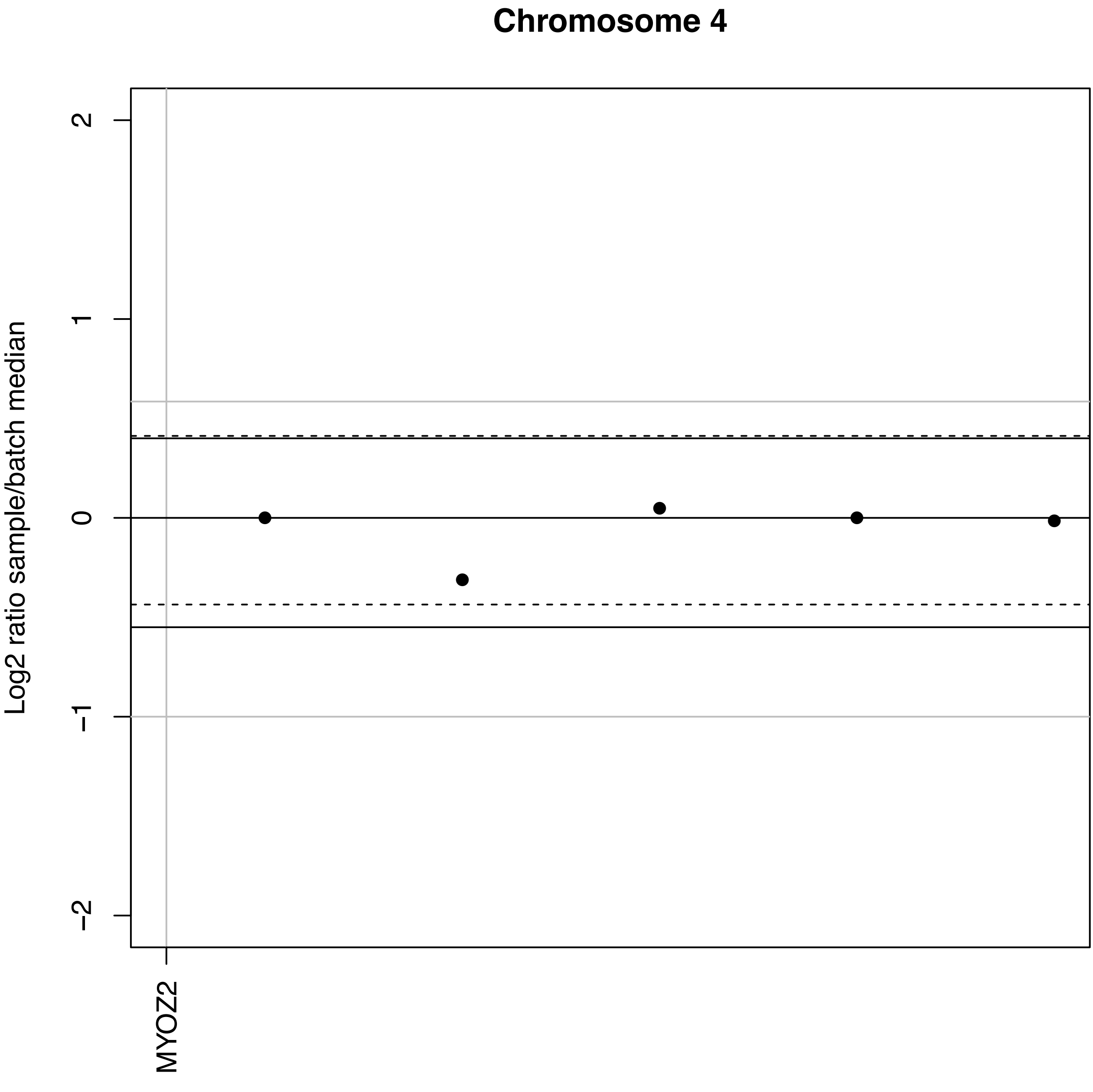

Case 26

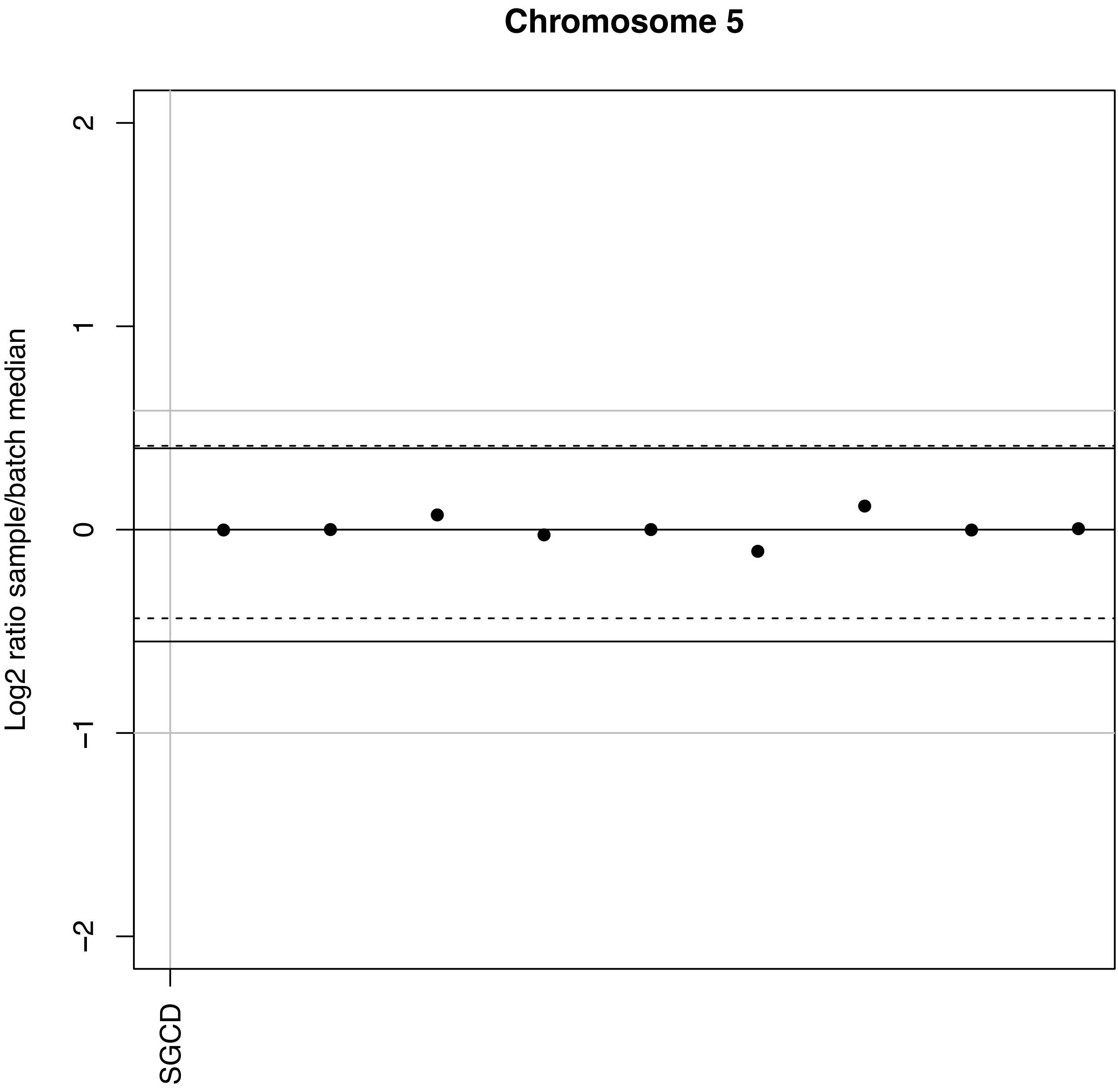

Case 26

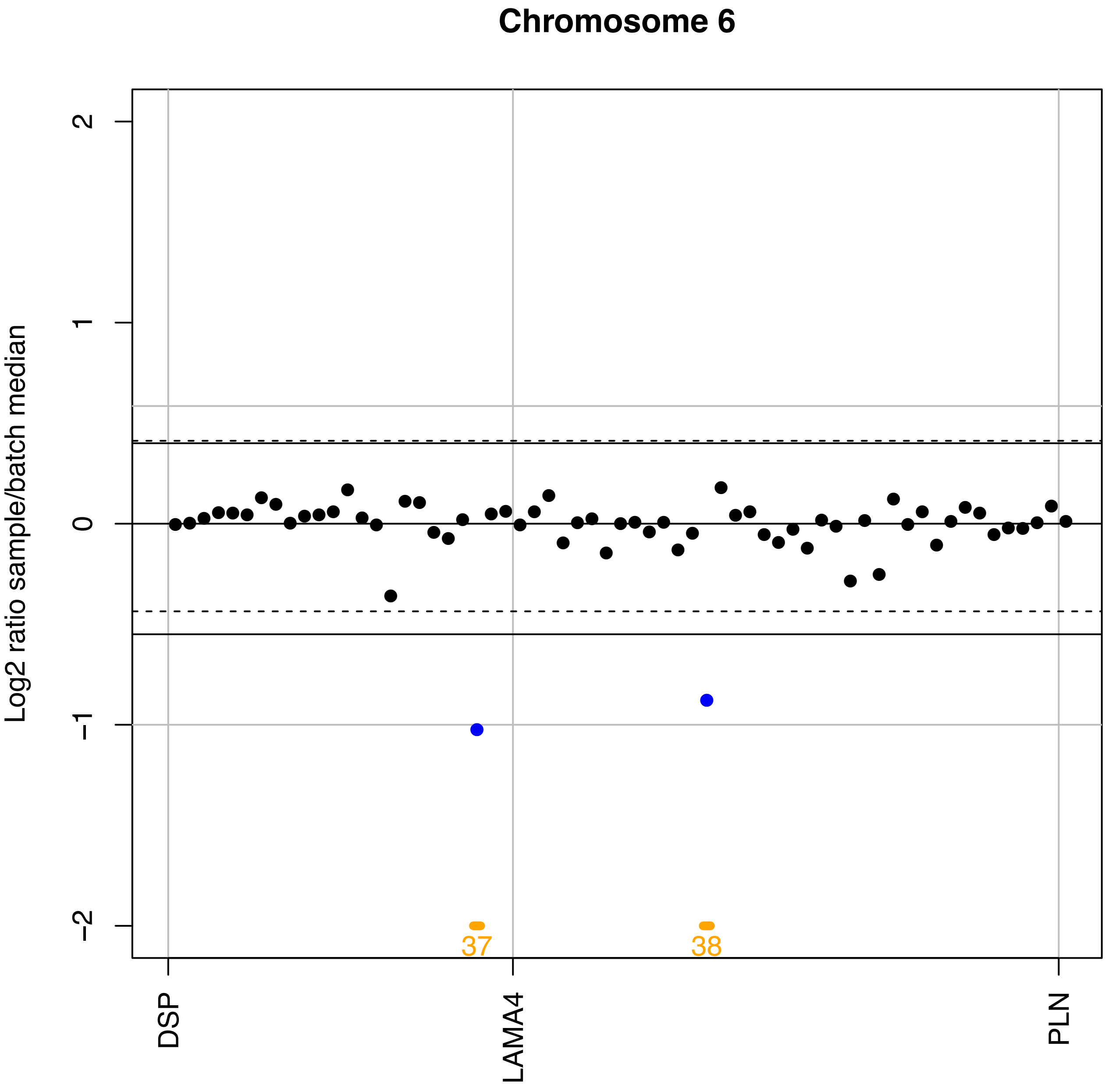

Chromosome 6
Log2 ratio sample/batch median
2
1
0
−1
−2
37
38
DSP
LAMA4
PLN

Case 26

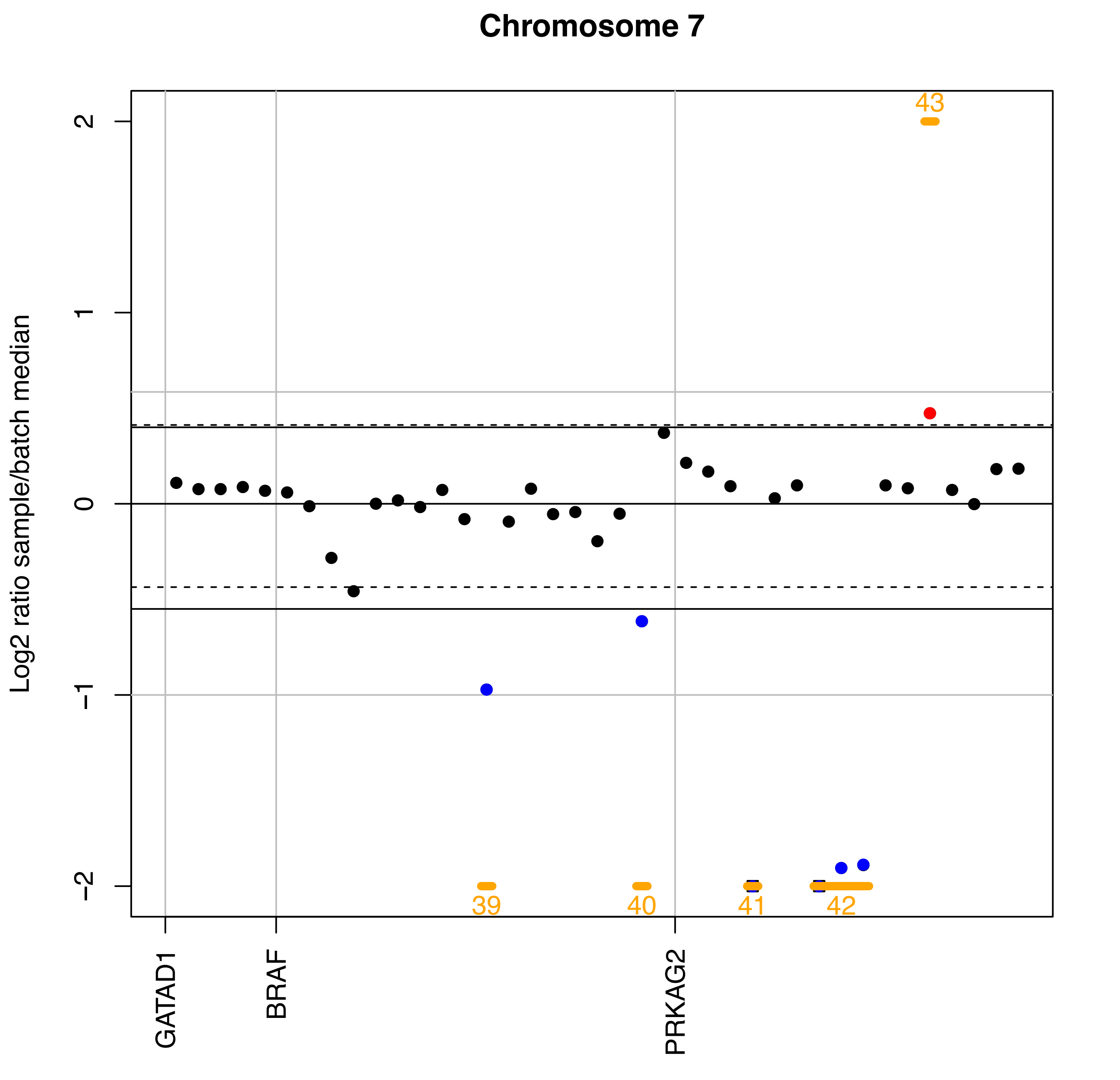
Chromosome 7
Log2 ratio sample/batch median
2
1
0
−1
−2
43
39
40
41
42
GATAD1
BRAF
PRKAG2

Case 26

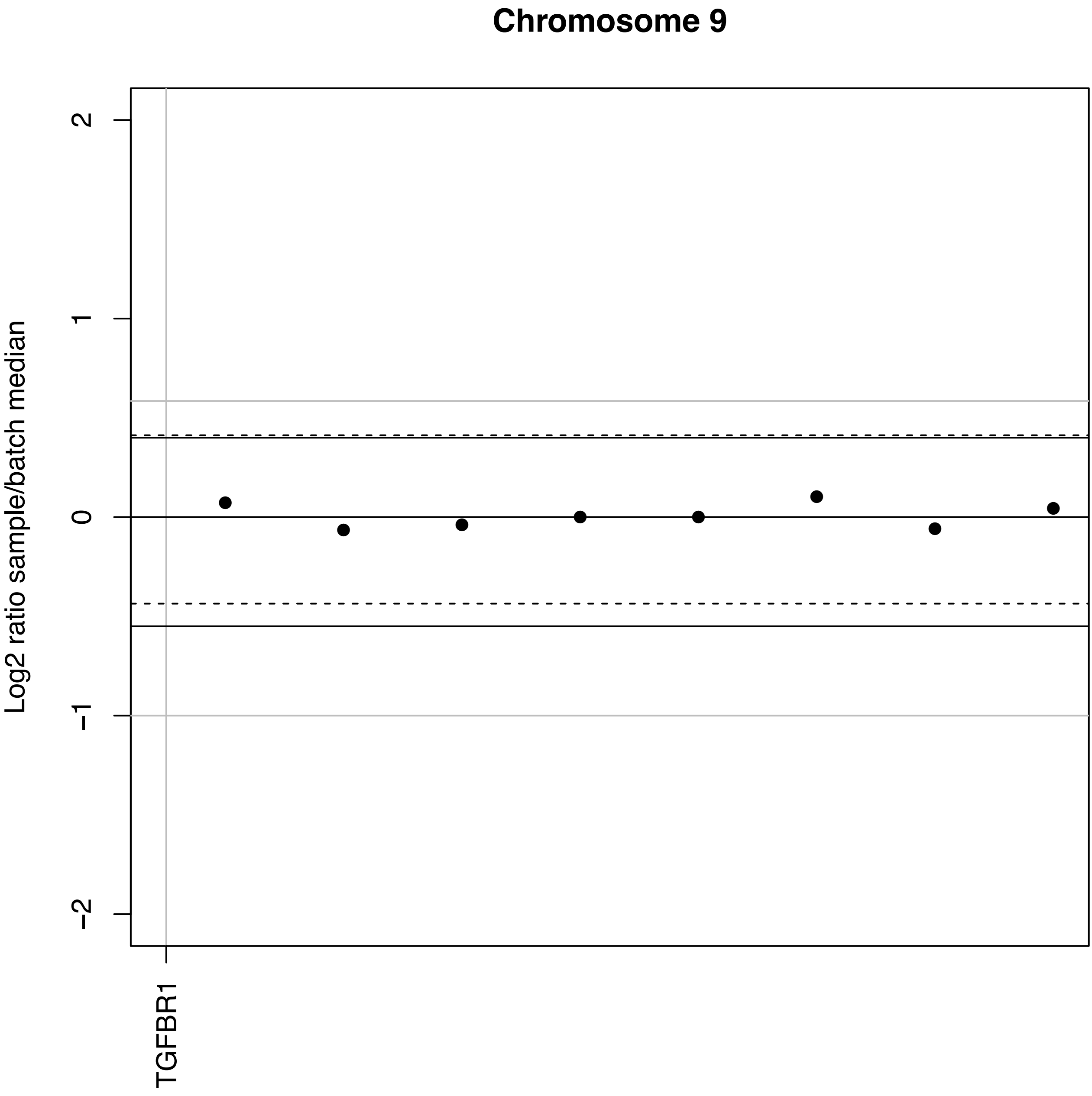

Case 26

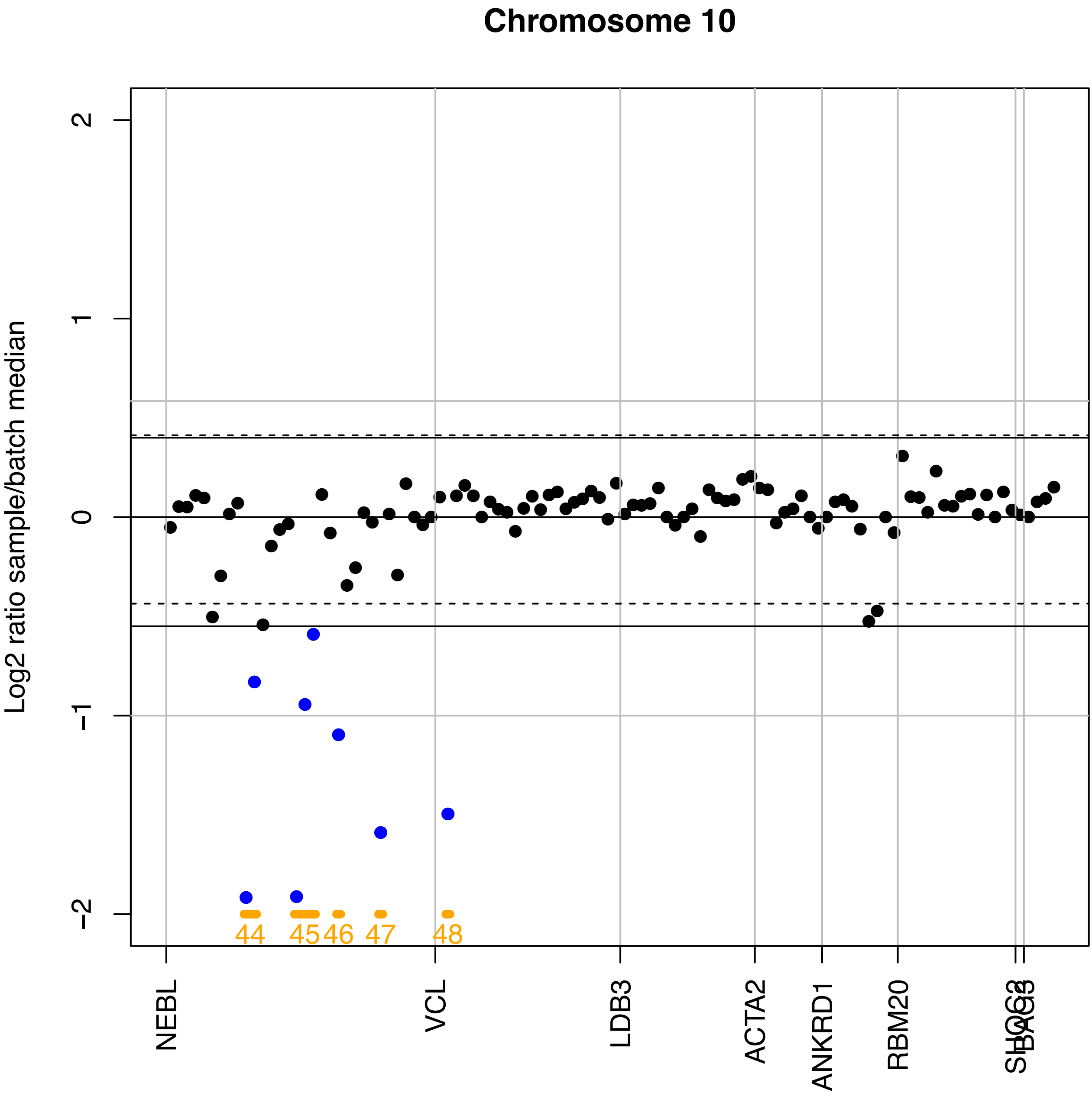
Chromosome 10
Log2 ratio sample/batch median
2
1
0
−1
−2
44
45
46
47
48
NEBL
VCL
LDB3
ACTA2
ANKRD1
RBM20

Case 26

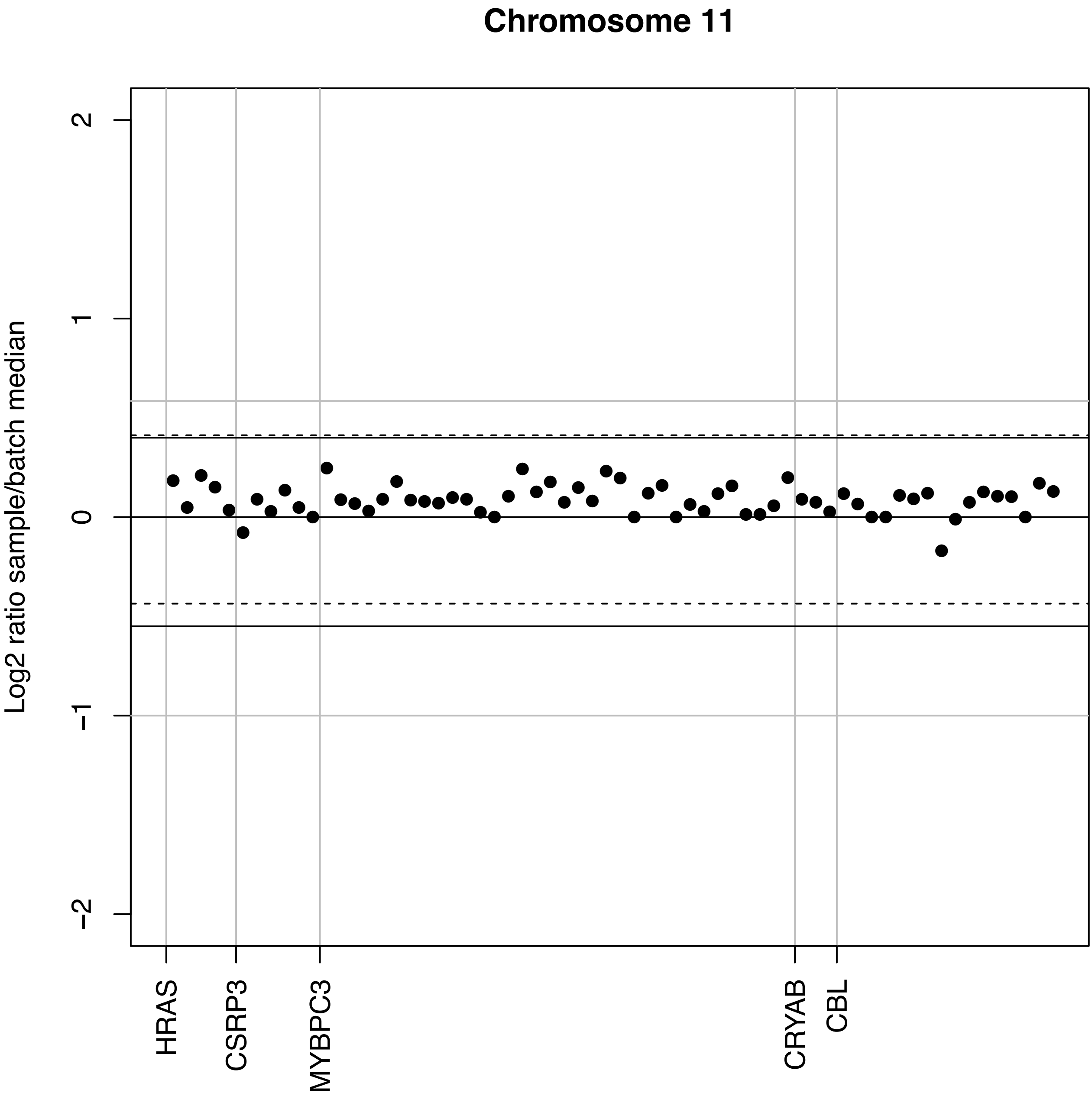

Chromosome 11
Log2 ratio sample/batch median
2
1
0
−1
−2
HRAS
CSRP3
MYBPC3
CRYAB
CBL

Case 26

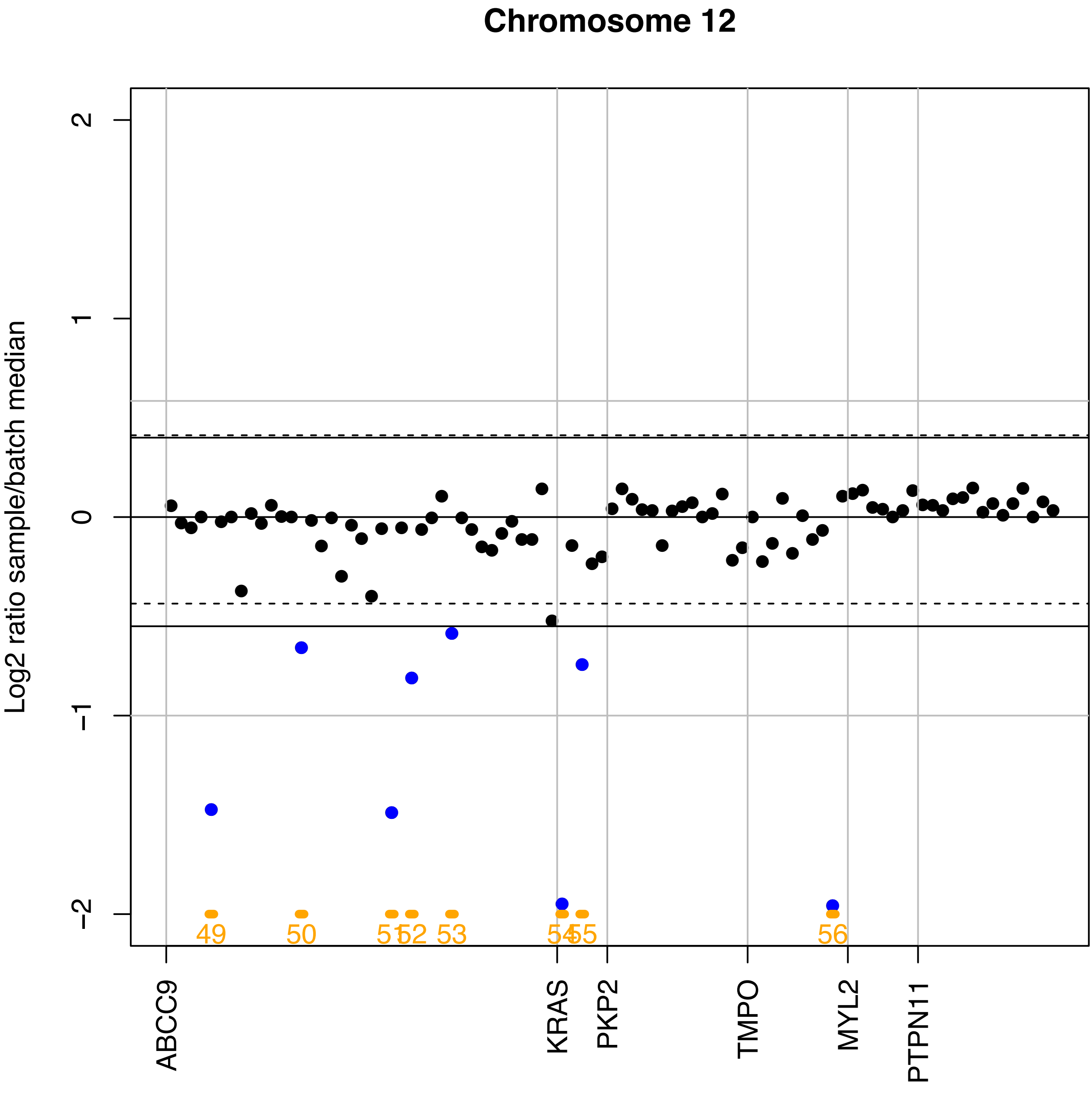

Chromosome 12
Log2 ratio sample/batch median
2
1
0
−1
−2
49
50
51
52
53
54
55
56
ABCC9
KRAS
PKP2
TMPO
MYL2
PTPN11

Case 26

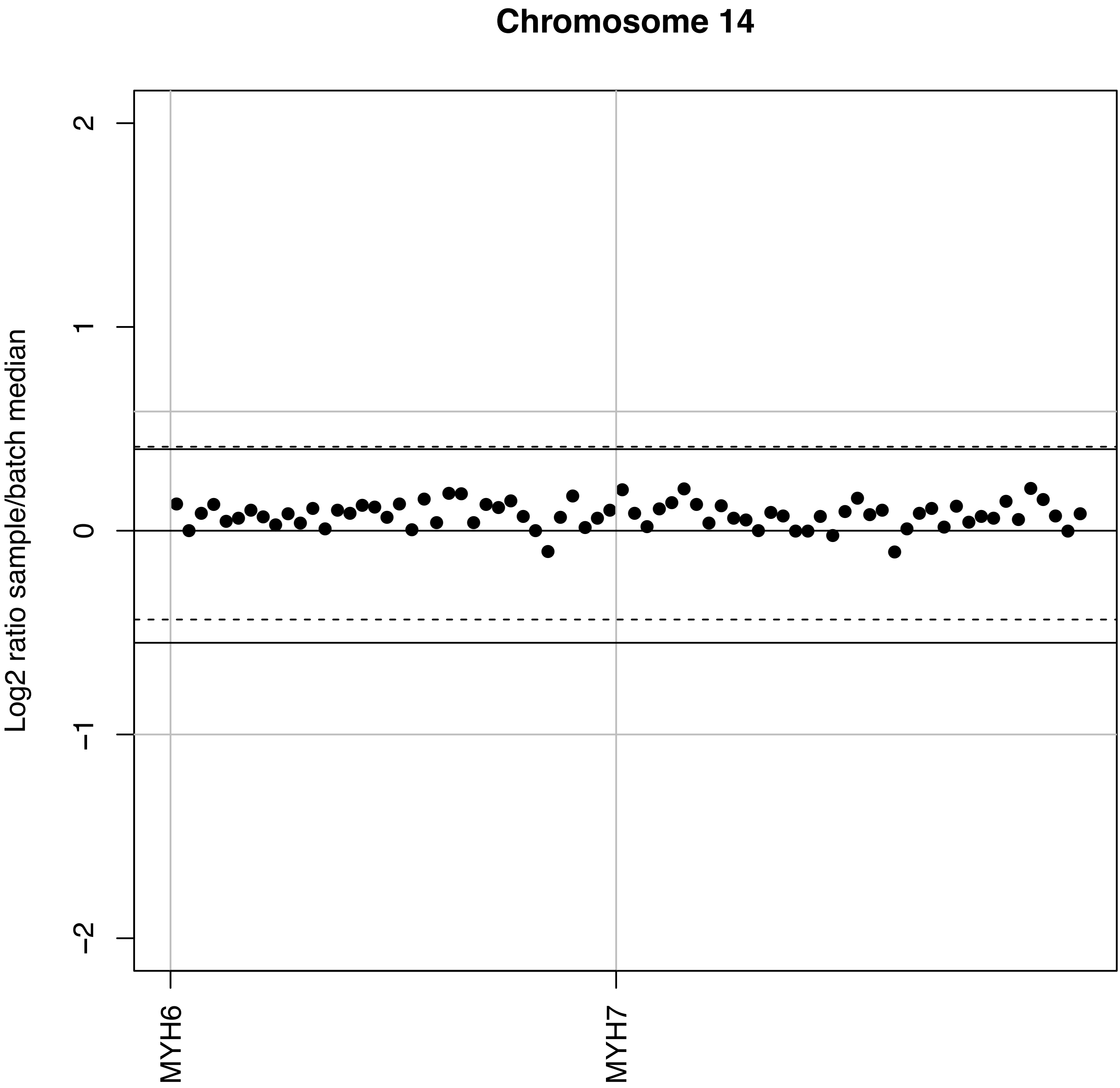

Chromosome 14
Log2 ratio sample/batch median
2
1
0
−1
−2
MYH6
MYH7

Case 26

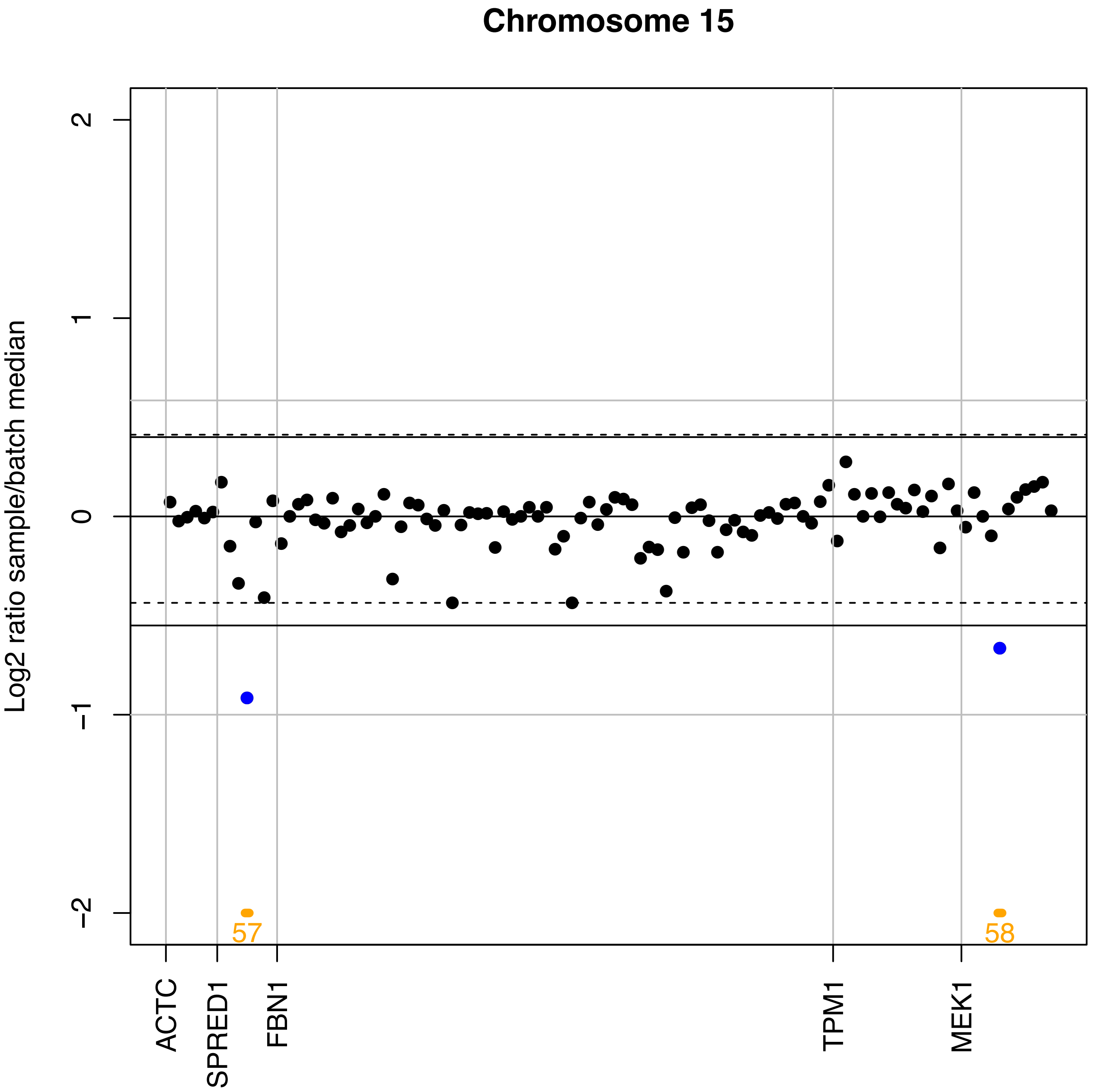

Chromosome 15
Log2 ratio sample/batch median
2
1
0
−1
−2
57
58
ACTC
SPRED1
FBN1
TPM1
MEK1

Case 26

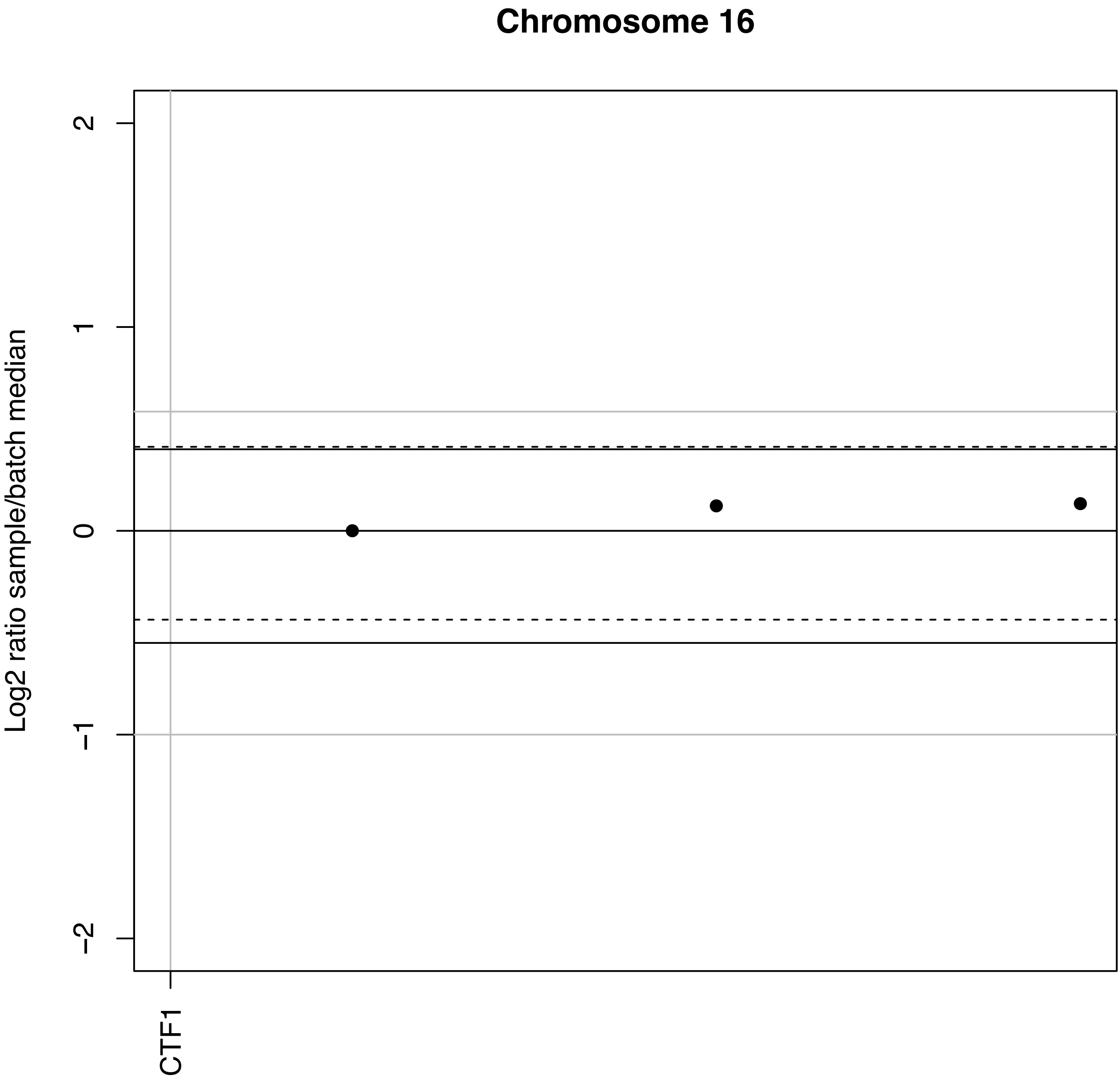

Case 26

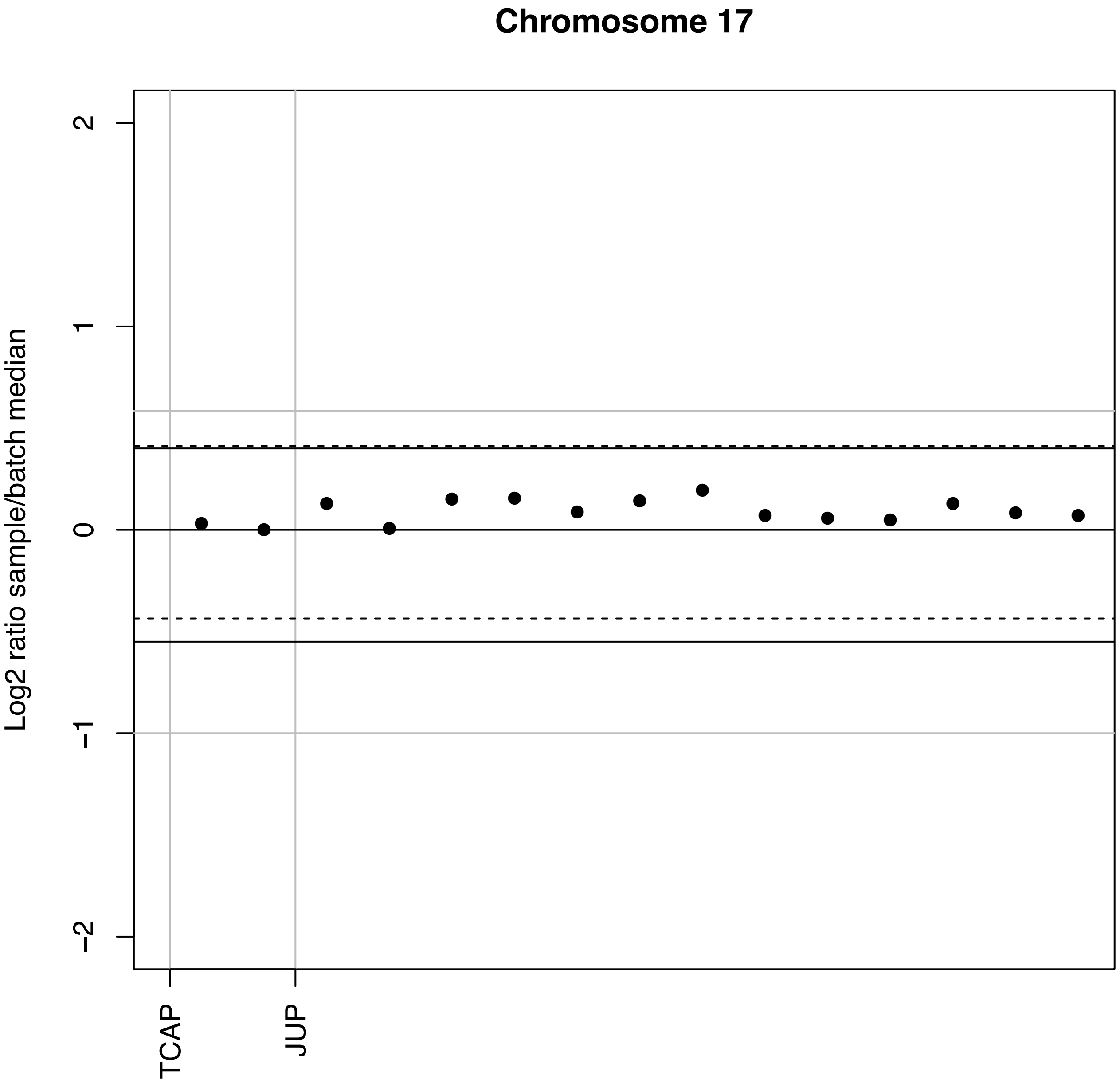
Chromosome 17
Log2 ratio sample/batch median
2
1
0
−1
−2
TCAP
JUP

Case 26

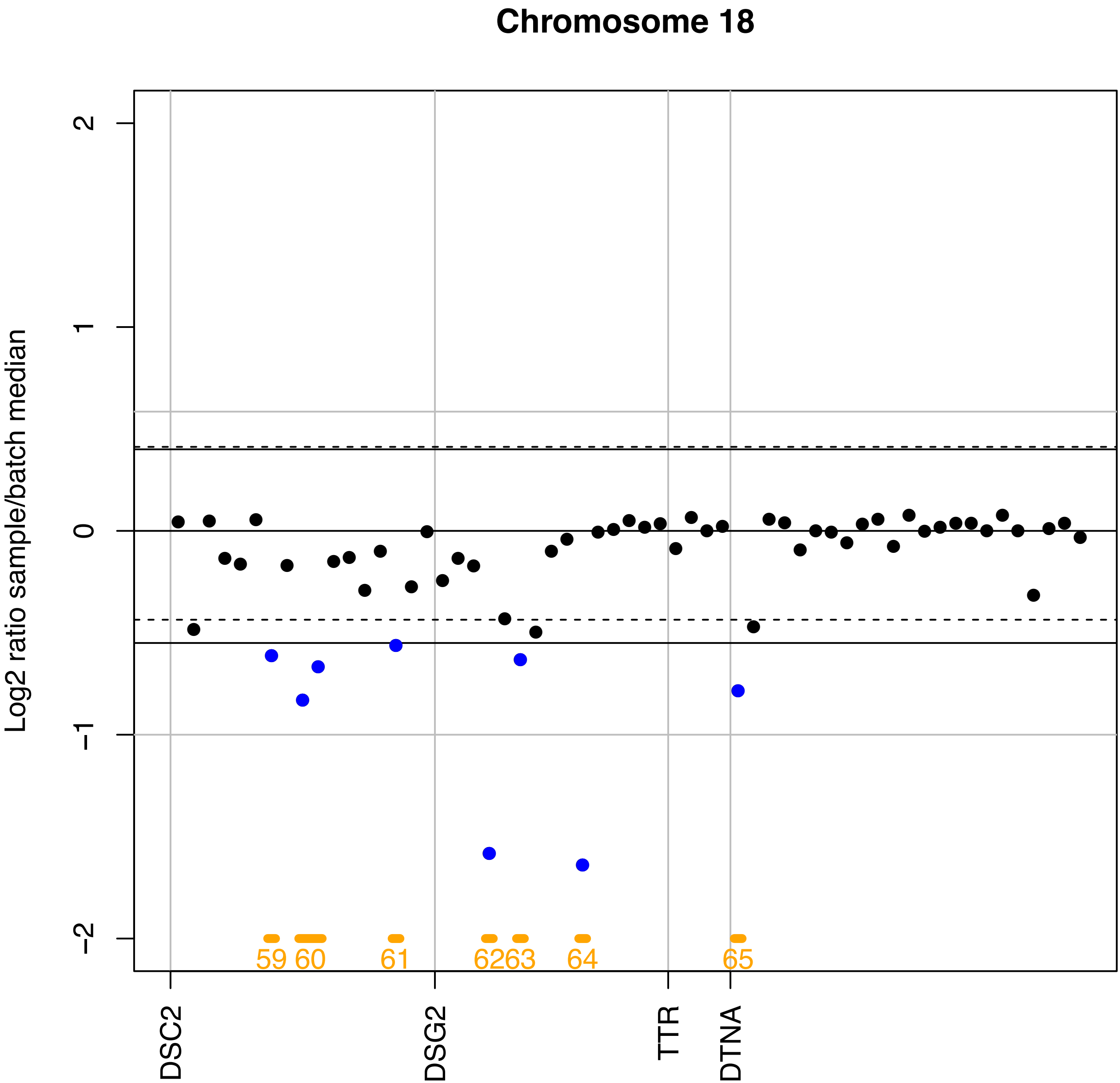

Chromosome 18
Log2 ratio sample/batch median
2
1
0
−1
−2
59
60
61
62
63
64
65
DSC2
DSG2
TTR
DTNA

Case 26

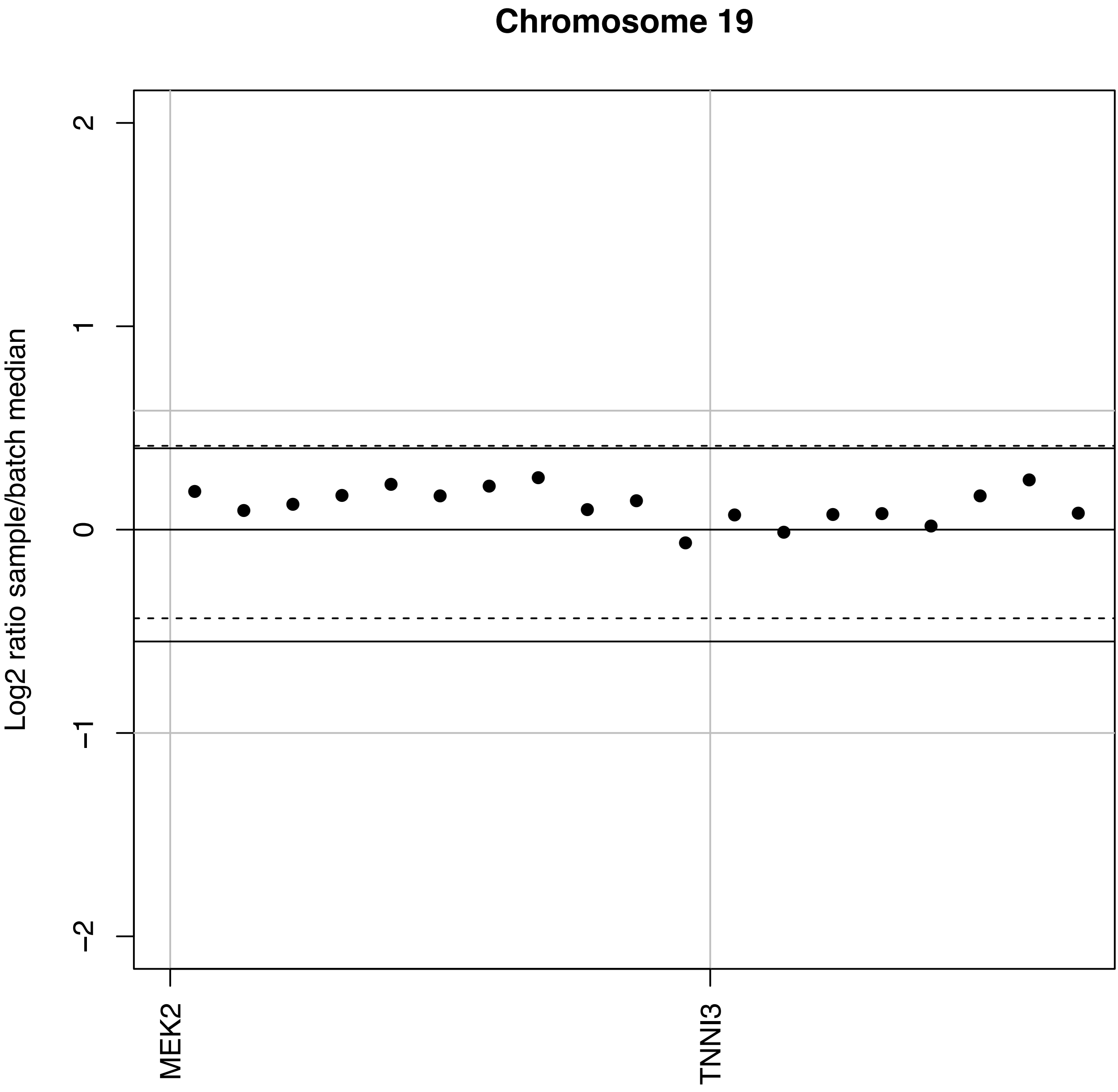
Chromosome 19
Log2 ratio sample/batch median
2
1
0
−1
−2
MEK2
TNNI3

Case 26

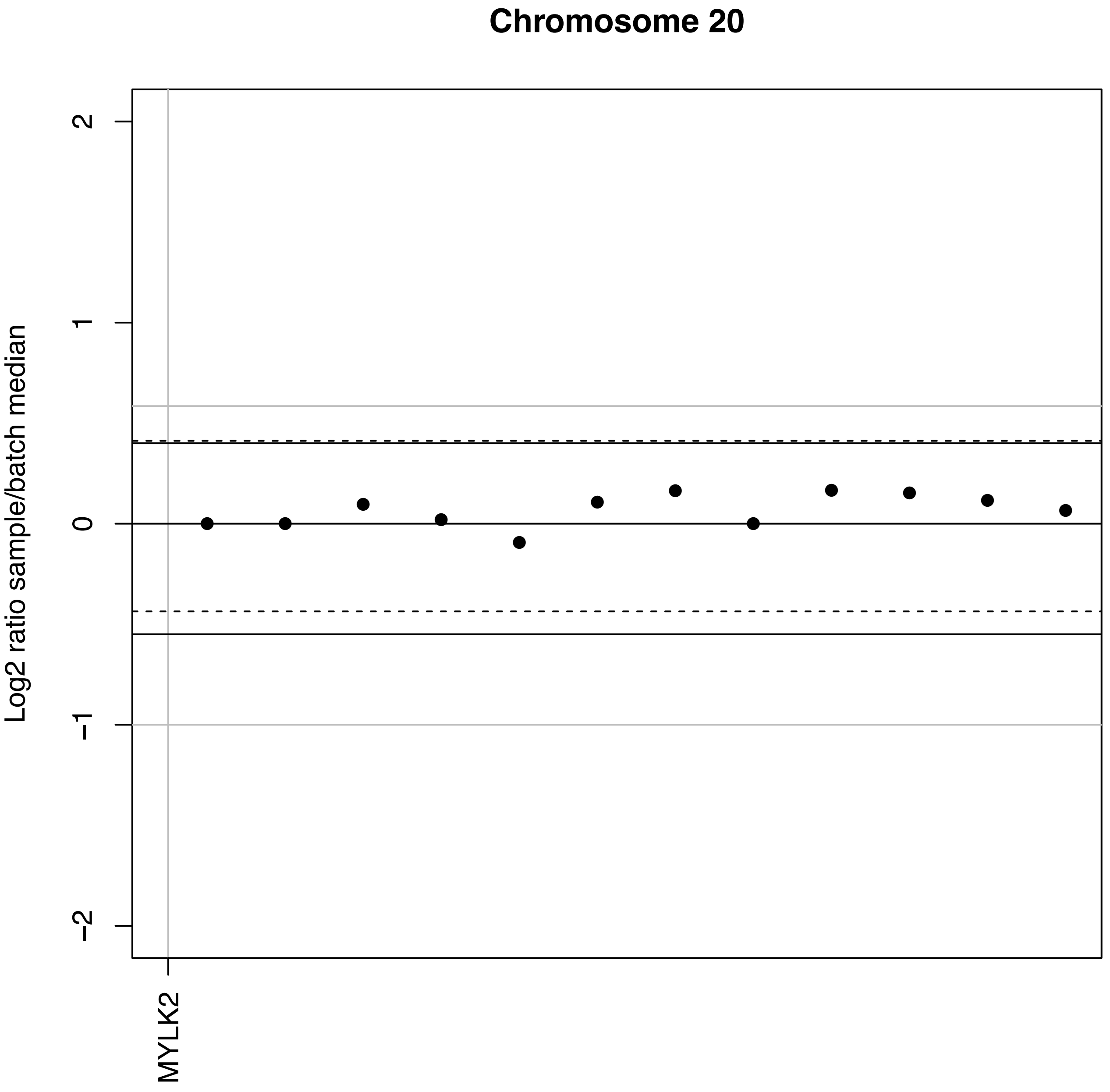

Case 26

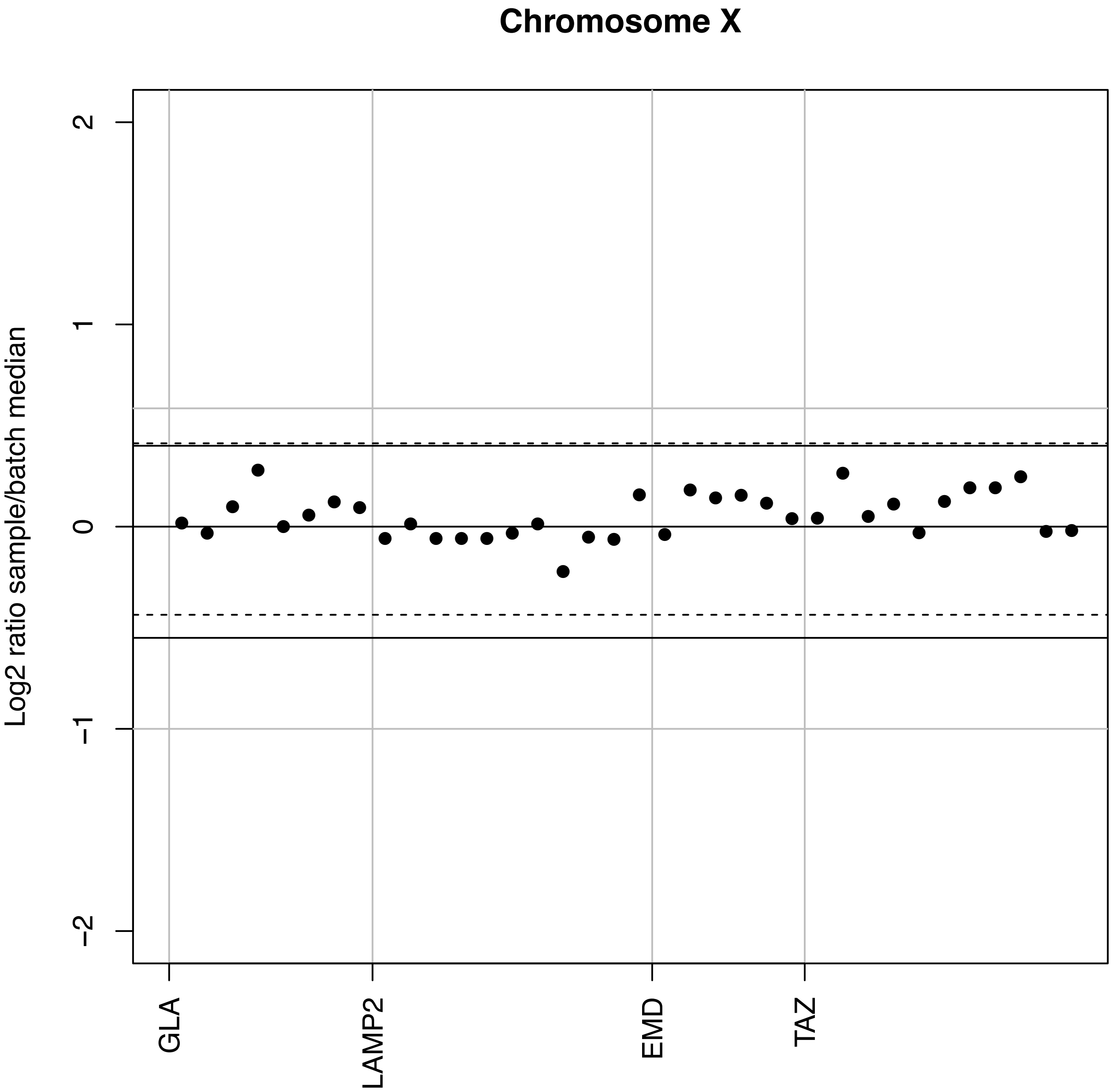

Chromosome X
Log2 ratio sample/batch median
2
1
0
−1
−2
GLA
LAMP2
EMD
TAZ
