# Supplementary material for: VisCap: inference and visualization of germ-line copy-number variants from targeted clinical sequencing data
Source: Genet Med. 2015 Dec 17;18(7):712–9. doi: 10.1038/gim.2015.156 (PMC4940431; doi:10.1038/gim.2015.156)
Supplement: Supplementary Information [file gim2015156x1.zip › Supplementary Tables S1 to S2.doc]

**SUPPLEMENTARY MATERIAL**

Supplementary Figure 1 – Receiver operating curve generated from training cohort used to select log2ratio thresholds for detecting copy number gains and losses in validation cohort

Supplementary File 1 – Software code, written in R.

Supplementary File 2 – Slide deck presentation used to train visual reviewers

Supplementary File 3 – A set of VisCap plots from samples with copy number variants used to evaluate training and effectiveness of visual scoring

Supplementary Table 1 - Description of file outputs

| **Output filename** | **Description** |
| --- | --- |
| VisCap_run_info.xls | Provides a complete summary of configuration used to run the VisCap analysis |
| QC_cnv_heatmap.pdf | Compares normalized, fractional coverage of each exon across all samples |
| QC_cnv_boxplot.pdf | Displays the distribution of log2 ratios for each sample by box-and-whisker plots |
| QC_cnv_boxplot.xls | Provides the summary metrics and pass/fail status for each sample in the batch based on the analysis of all genes in the region of interest |
| QC_chrX_pre-scale.pdf | Shows the average coverage of all data points on the X chromosome for males and females in the batch before any scale or compensation for males only having one X. All males will look deleted as compared to females. |
| QC_chrX_pre-scale.xls | Provides the pre-scale summary metrics and pass/fail status for each sample based on the analysis of X chromosome genes in the region of interest |
| QC_chrX_post-scale.pdf | Shows the average coverage of all data points on the X chromosome for males and females in the batch after any scale or compensation for males only having one X. Males and females should behave similarly. |
| QC_chrX_post-scale.xls | Provides the post-scale summary metrics and pass/fail status for each sample based on the analysis of X chromosome genes in the region of interest |
| sexes.xls | Provides computationally predicted patient genders based on pre-scale X plots |
| cnv_boxplot_outliers.xls | Contains all calls for all intervals across all samples [losses=-1, copy-neutral=0, gains=1] |
| log2_ratio_table.xls | Contains all log2 ratios for all intervals across all samples in the batch |
| *.plot.pdf  [one file per sample] | Displays the log2 ratios across all intervals overlaid with the threshold lines used for copy number variation detection.  * = input filename of the DepthOfCoverage file |
| *.cnvs.xls  [one file per sample] | Provides the metrics, genomic coordinates, and gene/exon identity for each copy number variation detected.  * = input filename of the DepthOfCoverage file |

Supplementary Table 2 – Summary of extended negative call validation using Illumina BeadArray

| **Case ID** | **VisCap** | | | | **Illumina Omni2.5-8 BeadArray** | | | |
| --- | --- | --- | --- | --- | --- | --- | --- | --- |
|  | **Panel** | **CNV call, interval range** | **Intervals within CNV** | **Median log2 ratio** | **CNV call overlapping VisCap Panel; all genes within CNV segment** | **Genomic segments (hg19)** | **Probes** | **Median log ratio** |
| R-1546 | Oto | None |  |  | None |  |  |  |
| R-1547 | PCM | None |  |  | None |  |  |  |
| R-1548 | Oto | None |  |  | None |  |  |  |
| R-1549 | Oto | None |  |  | None |  |  |  |
| R-1550 | Oto | None |  |  | None |  |  |  |
| R-1551 | Oto | None |  |  | None |  |  |  |
| R-1552 | Oto | None |  |  | None |  |  |  |
| R-1553 | Oto | None |  |  | None |  |  |  |
| R-1554 | Oto | None |  |  | None |  |  |  |
| R-1555 | PCM | None |  |  | None |  |  |  |
| R-1556 | PCM | Gain, *VCL* exons 2-8 | 7 | 0.55 | Gain; *VCL, C10orf55* | chr10:75675252-75847830 | 79 | 0.18 |
| R-1557 | PCM | Loss, *MYBPC3* exons 12-20 | 8 | -1.0 | Loss; *MYBPC3* | chr11:47361094-47367682 | 7 | -0.48 |
| R-1558 | Oto | Loss, *TMPRSS3* exons 2-5  Loss, *TMPRSS3* exon 13 | 4  1 | -4.1  -4.8 | Loss; *TMPRSS3, UBASH3A*  Loss; *TMPRSS3, TFF1* | chr21:43807803-43836288  chr21:43775347-43793000 | 50  44 | -4.1  -4.4 |
| R-1559 | PCM | Loss, *LDB3* exons 1-16 | 16 | -0.88 | Loss; *LDB3*, 49 others | chr10:81497419-89135287 | 6,174 | -0.36 |
| R-1560 | PCM | Gain, *RAF1* exons 2-12 | 11 | 0.51 | Gain; *RAF1, TMEM40* | chr3:12627035-12807420 | 108 | 0.24 |
| R-1561 | PCM | Gain, *TTN* exons 193-233  Gain, *TTN* exons 236-244 | 40  9 | 0.41  0.41 | None, but confirmed by ddPCR |  |  |  |
